# Enzyme (α-Glucosidase, α-Amylase, PTP1B & VEGFR-2) Inhibition and Cytotoxicity of Fluorinated Benzenesulfonic Ester Derivatives of the 5-Substituted 2-Hydroxy-3-nitroacetophenones

**DOI:** 10.3390/ijms252211862

**Published:** 2024-11-05

**Authors:** Temitope O. Olomola, Jackson K. Nkoana, Garland K. More, Samantha Gildenhuys, Malose J. Mphahlele

**Affiliations:** 1 Department of Chemistry, College of Science, Engineering and Technology, University of South Africa, Private Bag X06, Florida 1710, South Africa; tolomola@gmail.com (T.O.O.); nkoankj@unisa.ac.za (J.K.N.); 2 Department of Chemistry, Faculty of Science, Obafemi Awolowo University, Ile-Ife 220005, Nigeria; 3 Department of Life and Consumer Sciences, College of Agriculture and Environmental Sciences, University of South Africa, Private Bag X06, Florida 1710, South Africa

**Keywords:** sulfonic esters, characterization, enzymes (α-glucosidase; α-amylase; PTP1B & VEGFR-2), cytotoxicity, molecular docking

## Abstract

The prevalence of small multi-target drugs containing a fluorinated aromatic moiety among approved drugs in the market is due to the unique properties of this halogen atom. With the aim to develop potent antidiabetic agents, a series of phenylsulfonic esters based on the conjugation of the 5-substituted 2-hydroxy-3-nitroacetophenones **1a**–**d** with phenylsulfonyl chloride derivatives substituted with a fluorine atom or fluorine-containing (-CF_3_ or -OCF_3_) group were prepared. Their structures were characterized using a combination of spectroscopic techniques complemented with a single-crystal X-ray diffraction (XRD) analysis on a representative example. The compounds were, in turn, assayed for inhibitory effect against α-glucosidase, α-amylase, protein tyrosine phosphatase 1 B (PTP1B) and the vascular endothelial growth factor receptor-2 (VEGFR-2) all of which are associated with the pathogenesis and progression of type 2 diabetes mellitus (T2DM). The antigrowth effect of selected compounds was evaluated on the human breast (MCF-7) and lung (A549) cancer cell lines. The compounds were also evaluated for cytotoxicity against the African Green Monkey kidney (Vero) cell line. The results of an in vitro enzymatic study were augmented by molecular docking (in silico) analysis. Their ADME (absorption, distribution, metabolism and excretion) properties have been evaluated on the most active compounds against α-glucosidase and/or α-amylase to predict their drug likeness.

## 1. Introduction

The inhibition of activities of carbohydrate hydrolyzing enzymes, namely, α-glucosidase and α-amylase is an important strategy for the treatment of insulin-independent T2DM to control postprandial blood glucose levels in diabetes patients [1]. α-Amylase is present in the saliva and pancreatic juice and this enzyme digests starch into maltose, which in turn, is digested into glucose by α-glucosidase in the intestine [2]. In a state of metabolic disorder, these enzymes become abnormally expressed hence they represent important biochemical targets in antidiabetic drug development [1]. α-Amylase inhibitors retard the digestion and absorption of starch in the gastrointestinal tract. α-Glucosidase inhibitors, on the other hand, inhibit α-glucose release from complex carbohydrates present in the diet. This process, in turn, delays the absorption of glucose and results in reduced postprandial plasma glucose levels and suppression of postprandial hyperglycemia (PPHG). A moderate inhibition of pancreatic amylase activity will avoid accumulation of non-digested carbohydrates and their fermentation by bacteria in the colon, which would result in diarrhoea and flatulence [3]. The drugs that exhibit dual inhibitory activity against these carbohydrate hydrolyzing enzymes, but with a moderate inhibitory effect against α-amylase will result in a better antihyperglycemic effect and significantly reduced or no gastrointestinal side effects [4,5]. Another important enzyme of interest is PTP1B which also plays a role in the onset of T2DM and obesity [6]. This intracellular non-receptor protein tyrosine phosphatase is overexpressed in insulin-targeted tissues such as the liver, fats and muscle [7]. Insulin resistance is characteristic of T2DM and it is caused mostly by impairment in the insulin receptor (IR) signal transduction pathway [8]. PTP1B becomes upregulated under hyperglycemic conditions and, in turn, decreases the phosphorylation of IR resulting in insulin resistance [8]. Diabetes is a disease of multifactorial origin which is also characterized by an overexpression of protein kinases, which makes the protein kinase family one of the most important drug targets against this metabolic disorder [9]. The inhibitors of VEGFR-2, for example, have been found to reverse type 1 diabetes mellitus (T1DM) [10] and T2DM [11], and to prevent the clinical manifestation of both disorders. Moreover, the VEGFR-2 tyrosine kinase is a key target for the early diagnosis of diabetes retinopathy. The latter is a leading cause of blindness in the working-age (20–65 years) group in the developed countries [12,13]. VEGFR-2 is over-expressed in various tumors compared to the normal endothelial cells [14]. Scientific and clinical studies link impaired glucose tolerance with cancer [15] and the anti–α-glucosidase inhibitors have been found to exhibit positive effects in the treatment of different types of cancers [16]. The drugs that can mitigate PPHG and also inhibit cancer will offer an opportunity for development as multi-target-directed ligands (MTDLs) against T2DM.

**Figure 1:**
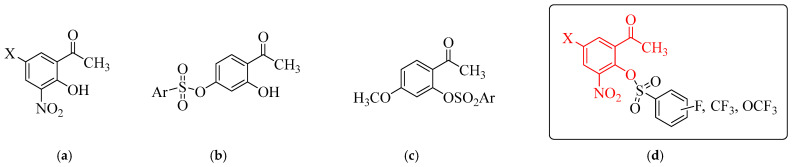
Chemical structures of hydroxyacetophenones (**a**), the sulfonic ester derivatives (**b**,**c**) and the target compounds (**d**). Red part represents *ortho*-hydroxyacetophenone scaffold.

Simple naturally occurring phenolic compounds such as the *ortho*-hydroxyacetophenones exhibit a wide range of biological properties including, antidiabetic, antimicrobial, anticancer, antimalarial, antioxidant, cardioprotective, analgesic, neuroprotective and anti-tyrosinase activities [17]. Among structurally diverse *ortho*-hydroxyacetophenones, our attention was drawn to derivatives containing a nitro group, a functional head prevalent in nitroaromatic compounds with antibacterial, antitumor, anti-ulcer, antiprotozoal, anthelmintic, anti-neurodegenerative and anxiolytic properties [17,18,19,20,21]. The strong electron withdrawing inductive and resonance effects of this functional head tend to polarize the drug molecules to interact with nucleophilic sites of proteins resulting in the inhibition of activities of a wide range of enzymes [20,21]. The 5-substituted (X = Cl or -CH_3_) 2-hydroxy-3-nitroacetophenones **a** (Figure 1), for example, previously inhibited the activities of the human carbonic anhydrase isoenzymes I and II, acetylcholinesterase and α-glucosidase at low nanomolar concentrations [22]. 2-Hydroxy-5-methyl-3-nitroacetophenone **a** (X = -CH_3_) has also been found to exhibit increased activity against the poorly differentiated lung adenocarcinoma (PC-14) cell line compared to the moderately differentiated lung adenocarcinoma (LC-2/ad) and well-differentiated bronchogenic adenocarcinoma (HLC-1) cell lines [23]. This compound was not cytotoxic towards the normal HUVEC (human umbilical vein endothelial cell) cell line and exhibited strong DPPH (2,2-diphenyl-1-picrylhydrazyl) radical scavenging activity. Molecular docking revealed that 2-hydroxy-5-methyl-3-nitroacetophenone could inhibit glutathione S-transferase, a potential drug target in cancer therapy and glutathione reductase an important antioxidant enzyme required to maintain the reduced glutathione/oxidized glutathione ratio [23]. Despite the increased biological activity associated with the hydroxy substituted acetophenones, their development as pharmaceutical products is hampered by their poor solubility and oral bioavailability [24,25]. These drawbacks are attributed to the unprotected hydroxyl group, which is also susceptible to intestinal and/or hepatic conjugation via glucuronidation or sulfation [25,26]. There is continued effort to manipulate and derivatize the structures of hydroxy substituted acetophenones with the aim to increase their biological activity, solubility and absorption or bioavailability, in turn, boost their therapeutic efficacy in vivo [24]. A weakly polar hydrophobic sulfonic ester (-OSO_2_R) grooup is present in several bioactive structures with antitumor, antidiabetic and antidepressant properties, and this group tends to trigger biological activity of sulfonic ester-based drugs and also increase their solubility [27,28,29]. The propensity of this functional head to engage in noncovalent bonding interactions with the amino acid residues such as lysine, histidine, serine and tyrosine affords sulfonic ester-based drugs their ability to inhibit the activities of various enzymes [28,30]. We have previously synthesized a series of fluorinated sulfonic esters of the generalized chemical structures **b** and **c** (Figure 1) derived from 2,4-dihydroxyacetophenone and 2-hydroxy-4-methoxyacetophenone, respectively [31]. A comparative study of their inhibitory effect with the non-fluorinated analogues confirmed the fluoro substituted phenylsulfonyl derivatives as the most active against the tested biomolecules linked to T2DM. It is envisaged that conjugation of hydroxyacetophenone scaffold with the weakly polar hydrophobic sulfonyl moiety will increase the antidiabetic activity, solubility and lipophilicity of the resultant sulfonic ester derivatives.

The unique properties associated with fluorine atom continue to be exploited in the design and development of small drug molecules of medicinal importance [32] including drugs that played a very crucial role in controlling the coronavirus disease-2019 (COVID-19) [33]. The presence of fluorinated substituents in small drug molecules has led to mechanism-based inhibitors for a wide variety of diseases and to chemotherapeutic drugs [34]. Numerous review articles [35,36,37,38,39,40,41] which underscore the medicinal importance of fluorine-containing compounds in the development of small drug molecules continue to be published. The hydrophobic and lipophilic properties of fluorine atom have been found to influence the drug conformation, membrane permeability, metabolic stability and solubility as well as the blood-brain barrier (BBB) penetration in the case of central nervous system agents [35,36,37,38,39,40,41]. This halogen atom has increased propensity to engage in multipolar contacts with the carbonyl carbon atom or the amide nitrogen atom in the backbone or side chain of enzymes or proteins, thus enabling fluorinated drugs to inhibit a wide range of enzyme targets [39,42]. Furthermore, its electron-withdrawing inductive effect facilitates the formation of intermolecular hydrogen bonds with amino acid residues, which stabilize the drug-receptor interactions [26]. The aforementioned biological properties associated with the 2-hydroxy-3-nitroacetophenones and the fluorine-containing sulfonic esters derived from the *ortho*-hydroxyacetophenones encouraged us to employ the C-5 substituted (X = F, Cl, Br or -CH_3_) 2-hydroxy-3-nitroacetophenones (**a**) as scaffolds for conjugation with fluorinated benzenesulfonyl chlorides to afford the sulfonic esters of the generalized chemical structure (**d**). The presence of the lone pair electrons and a region with positive charge on chlorine or bromine atom termed a σ-hole is envisaged to facilitate hydrogen bonding and halogen bonding interactions, respectively, to stabilize the receptor-drug interactions [43]. The hydrophobic methyl group can induce favourable conformational change of the drug molecule (magic methyl effect) to influence the drug’s biological activity, solubility, selectivity and the ADME properties as well as its toxicity [39,44]. Our continued interest in exploring the biological activity of small drug molecules as potential antidiabetic agents led us to evaluate the prepared acetophenone sulfonic ester derivatives for dual inhibitory activity against α-glucosidase and α-amylase. It is envisaged that targeting hyperglycemia along with other biomolecular targets associated with the pathogenesis and progression of T2DM could be a significant strategy for the treatment of this metabolic disorder. As a result, the derivatives that exhibited strong inhibitory effect on α-glucosidase were screened for inhibitory activity against α-amylase, PTP1B and VEGFR-2, respectively. These derivatives were, in turn, evaluated for antigrowth effect on the MCF-7 and A549 cancer cell lines to establish their anticancer activity. These compounds were also assayed for cytotoxicity against the Vero cell line to establish their selectivity in vitro. Molecular docking (in silico) study was undertaken on the most active ligands to predict their fit and orientation within the binding sites of the test enzymes. Their pharmacokinetic (ADME) properties have been evaluated to predict their drug likeness.

## 2. Results and Discussion

### 2.1. Chemical Synthesis and Characterization

**Scheme 1:**
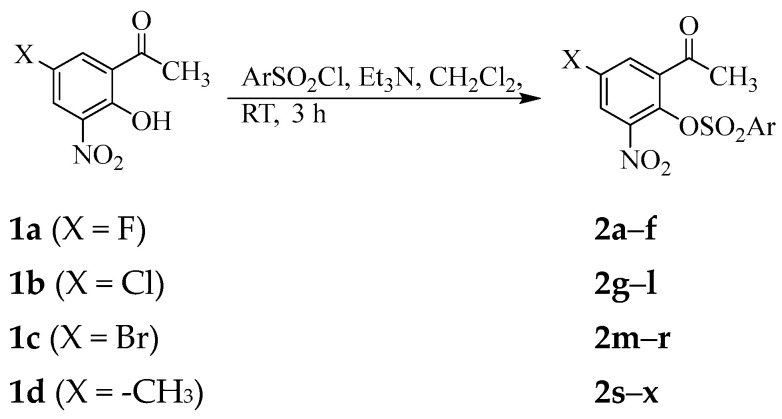
Synthesis of acetophenone sulfonic ester derivatives **2a**–**x**.

Chemists continue to adopt and/or improve the existing methodologies to synthesize various kinds of sulfonic esters with therapeutic potential. In this study, we adapted a sure-fire method towards the acetophenone sulfonic ester derivatives **2a**–**x** involving conjugation of the fluorinated phenylsulfonyl chloride precursors with the 5-substituted 2-hydroxyacetophenones as shown in Scheme 1. An initial attempt to condense the 5-fluoro-2-hydroxyacetophenone **1a** (1 equiv.) with 1.2 equiv. of 2-fluorophenylsulfonyl chloride in the presence of pyridine as a base in dichloromethane at room temperature (RT) or under reflux for 12 h resulted in the recovery of the starting material. The use of triethylamine (2 equiv.) as a base at RT to capture the released HCl, on the other hand afforded after 3 h, a product characterized using ^1^H- and ^13^C NMR spectroscopy as the corresponding acetophenone sulfonic ester derivative **2a**. The reaction conditions employing triethyl amine as a base in dichloromethane at RT were applied to the 5-substituted 2-hydroxyacetophenones **1a**–**d** with phenylsulfonyl chlorides substituted with a fluorine atom, fluoroalkyl (-CF_3_) or fluorine-containing alkoxy (-OCF_3_) group as well as a 4-methoxybenzenesulfonyl chloride. Aqueous work-up followed by recrystallization of the crude solids from ethanol afforded the corresponding sulfonic ester derivatives **2a**–**x** as pure products. The designation of substituents and substitution pattern of the acetophenone sulfonic ester derivatives **2** are presented in Table 1 and the corresponding copies of NMR spectra are enclosed as Figure S1 of the Supplementary Materials. The ^1^H NMR spectra of compounds **2** are characterized by the presence of an increased number of signals in the aromatic region. These distinguished the structures of these compounds from those of the corresponding substrates **1**. The ^1^H NMR spectra of the non-fluorinated analogues **2f**, **2l**, **2r** and **2x** (Ar = -C_6_H_4_(4-OMe)) showed an intense singlet around δ = 3.91 ppm, which corresponds to protons of the methoxy group. The multiplet signals due to carbon–fluorine coupling were clearly resolved and separated in the ^13^C NMR spectra of the fluorinated analogues. This facilitated an unambiguous assignment of these signals based on the differences in their chemical shifts and coupling constant (^1^*J*_CF_ (*ipso*) >> ^2^*J*_CF_ (*ortho*) > ^3^*J*_CF_ (*meta*) > ^4^*J*_CF_ (*para*)) values. The corresponding carbon-fluorine coupling constant values were found to fall within the range of the reported values for the fluorobenzene analogues [45]. Incorporation of a fluorinated benzene sulfonic ester moiety on the scaffold **2** was further confirmed by an additional singlet in the ^19^F NMR spectra of sub-class **2a**–**e** or a singlet in the case of the sub-series **2g**–**k**, **2m**–**q** and **2s**–**w**. Such isolated singlet ^19^F resonances are useful in fragment-based drug design and in vivo applications to probe ligand-protein binding, pharmacokinetics and drug metabolism [46,47,48].

**Figure 2:**
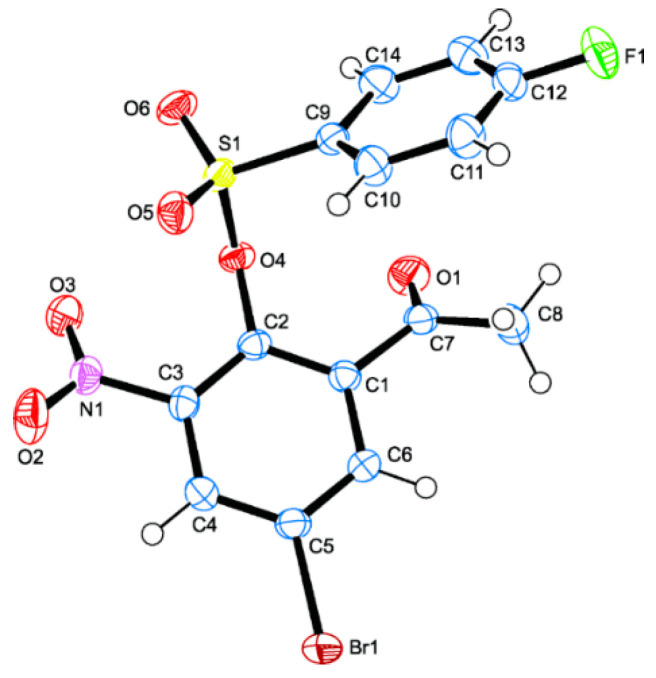
Molecular diagram of **2o** (CCDC 2380079) with displacement ellipsoids drawn at the 50% probability level. The atom numbering scheme used differs from the systematic numbering of this class of compounds.

The strength of a drug molecule to bind to the target is influenced by its ability to adopt the right conformation to fit well into the receptor binding site to inhibit or enhance biological activity [49,50,51]. The sulfonic ester group, for example, is known to control the conformation of sulfonic ester-based compounds to facilitate increased hydrophilic and hydrophobic contacts with amino acid residues in the receptors compared to the surrogate ester functional group [31,51]. Single crystals of **2o** suitable for X-ray diffraction (XRD) analysis were obtained by slow evaporation of chloroform, and the conformation of the test compounds was thus confirmed by XRD method (Figure 2). The crystal data and structure refinement of this compound are represented in Table S1 of the Supplementary Materials. Compound **2o** crystallized in a monoclinic space group *P*21c with Z = 4. The conformation of the O–C bond in the C–SO_2_–O–C fragment of the structure of **2o** adopted a *trans* and *gauche* orientation with respect to the two S=O bonds. The observed S=O bond length values of 1.4254(14) Å and 1.4208(13) Å for S(1)-O(5) and S(1)-O(6), respectively, compare favourably with values previously obtained for the analogous 4-sulfono-3-methoxycinnamaldehydes [51]. The sulfonate group has deviated from the coplanarity of the acetophenone scaffold with a torsion angle, C(2)-O(4)-S(1)-C(9), of 83.58(13)° between the two rings due to the distorted tetrahedral geometry of the sulfur atom. This deviation will probably result in increased aromatic-aromatic interactions with some of the amino acid residues in the active sites of the target enzymes.

Our interest in the exploration of small drug molecules as potential antidiabetic agents prompted us to evaluate the sulfonic esters **2a**–**x** through enzymatic assays in vitro for inhibitory effect against some of the molecular targets linked to the onset and progression of T2DM, namely, α-glucosidase, α-amylase, PTP1B and VEGFR-2. The non-fluorinated analogues **2f**, **2l**, **2r** and **2x** with Ar = -C_6_H_4_(4-OMe) were included as a comparative study to confirm if the fluorine atom or fluorine-based groups influenced the biological activity of the fluorinated sulfonate ester derivatives against these enzymes. The structure activity relationship (SAR) of the series of the sulfonic esters comprising of the 5-fluoro (**2a**–**f**), 5-chloro (**2g**–**l**), 5-bromo (**2m**–**r**) and 5-methyl (**2s**–**x**) sub-series has been rationalised by considering the substituent at the C-5 position of the acetophenone ring and the type of the substituent on the benzene sulfonyl arm.

### 2.2. Biological Activity Studies with SAR

#### 2.2.1. Inhibition of α-Glucosidase

The biological significance of the *ortho*-hydroxyacetophenones **1a**–**d** and the sulfonic ester derivatives **2a**–**x** as potential antidiabetic agents was first evaluated in vitro through an enzymatic assay against α-glucosidase from *Saccharomyces cerevisiae.* The inhibition curves used to calculate the IC_50_ values (the concentration of the drug that inhibits 50% of α-glucosidase activity) are enclosed as Figure S2. The corresponding IC_50_ values determined in the concentration range of 1–50 μM against acarbose as a reference standard are represented in Table 2. The 5-halogeno-3-nitro substituted *ortho*-hydroxyacetophenones **1a**, **1b** and **1c** exhibited reduced inhibitory effect against α-glucosidase compared to acarbose (IC_50_ = 6.4 ± 0.134 µM) with the IC_50_ values of 16.9 ± 0.064, 27.2 ± 0.195 and 11.9 ± 0.010 μM, respectively. Although 2-hydroxy-5-methyl-3-nitroacetophone **1d** has been reported to inhibit α-glucosidase activity at low nanomolar concentrations [22], this compound exhibited a moderate inhibitory effect against α-glucosidase (IC_50_ = 8.2 ± 0.015 µM) compared to acarbose in the current assay. Improved, but variable activity against this enzyme was observed for most of the corresponding phenylsulfonyl substituted derivatives **2**. Compound **2a** bearing a 2-(fluorophenyl)sulfonyl group exhibited strong activity against α-glucosidase (IC_50_ = 5.6 ± 0.038 µM) compared to the isomeric 3-(fluorophenyl)sulfonyl **2b** and 4-(fluorophenyl)sulfonyl substituted derivative **2c** with the IC_50_ values of 8.6 ± 0.025 and 27.4 ± 0.044 µM, respectively. The presence of a trifluoromethyl group at the *para* position of the benzenesulfonyl moiety of **2d** resulted in strong inhibitory effect against this enzyme comparable to acarbose with the IC_50_ values of 6.4 ± 0.012 µM and 6.4 ± 0.134 µM, respectively. The 4-(trifluoromethoxyphenyl)sulfonyl substituted derivative **2e** exhibited the highest activity against this enzyme within this series with an IC_50_ value of 3.1 ± 0.043 µM. This fluorine-containing compound is also more active against this enzyme compared to the analogue **2f** (IC_50_ = 5.3 ± 0.159 µM) substituted with a 4-(methoxyphenyl)sulfonyl group. Derivative **2g** substituted with a 5-chloro and 2-(2-fluorophenyl)sulfonyl group exhibited strong inhibitory effect against this enzyme (IC_50_ = 4.2 ± 0.054 µM) compared to the isomeric *meta*- **2h** and *para*-(fluorophenyl)sulfonyl derivative **2i** with the IC_50_ values of 12.4 ± 0.041 and 10.6 ± 0.039 µM, respectively. The analogues **2j** (IC_50_ = 5.4 ± 0.030 µM) and **2k** (IC_50_ = 3.9 ± 0.031 µM) substituted with either a trifluoromethyl or trifluoromethoxy group at the *para* position of the benzenesulfonic arm, respectively, exhibited strong inhibitory effects against this enzyme within this series. A combination of the 5-chloro and the 4-(methoxyphenyl)sulfonyl group in **2l** (IC_50_ = 8.5 ± 0.043 µM) resulted in a weaker activity against this enzyme compared to the analogue **2k**. Compounds in the series **2m**–**r** substituted with a bromine atom at the C-5 position of the acetophenone ring exhibited relatively reduced activity against α-glucosidase compared to other sub-series with the IC_50_ values in the range 7.9 ± 0.044–20.5 ± 0.058 µM. Only compounds **2m**, **2p** and **2q** substituted with a 2-(fluorophenyl)sulfonyl-, 4-(trifluorophenyl)sulfonyl- or 4-(trifluorophenyl)sulfonyl group exhibited significant and comparable inhibitory effect against this enzyme with the IC_50_ values of 7.9 ± 0.044 µM), 8.0 ± 0.060 µM and 8.7 ± 0.075 µM, respectively. A combination of the 5-bromo and 4-(methoxyphenyl)sulfonyl group on the scaffold of **2r** resulted in significantly reduced activity for this compound with an IC_50_ value of 13.2 ± 0.043 µM. The isomers substituted with a 5-methyl group on the acetophenone ring and a fluorine atom at the *ortho* (**2s**), *meta* (**2t**) or *para* (**2u**) position of the phenylsulfonyl ring exhibited reduced inhibitory effect against α-glucosidase with the IC_50_ values of 18.1 ± 0.048, 13.1 ± 0.045 and 11.2 ± 0.045 µM, respectively. A combination of the 5-methyl group and a 4-(trifluoromethylphenyl)sulfonyl group on the scaffold of the analogue **2v**, on the other hand, resulted in strong activity against α-glucosidase with an IC_50_ value of 3.0 ± 0.014 µM. A strong activity against this enzyme was also observed for the 4-(trifluoromethoxyphenyl)sulfonyl substituted derivative **2w** with an IC_50_ value of 6.0 ± 0.034 µM. However, this compound was less active compared to the analogue **2x** (IC_50_ = 3.6 ± 0.029 µM) substituted with a 4-(methoxyphenyl)sulfonyl group.

Hitherto, sulfonic esters of the generalized structure **C** shown in Figure 1 exhibited significantly reduced inhibitory effect against α-glucosidase compared to the intramolecularly hydrogen bonded analogues **B** [31]. The observed increased activity of sulfonic esters **2** is attributed to the nitro group which probably polarized the acetophenone ring and facilitated interactions with nucleophilic sites of proteins resulting in enzyme inhibition. The difference in activity of these compounds against α-glucosidase resulted from the type of the substituent and substitution pattern on the benzenesulfonyl ring and also on the C-5 position of the acetophenone scaffold. This SAR analysis revealed the 2-(fluorophenyl)sulfonyl substituted derivatives (**2a**, **2g**, **2m** and **2s**) as the most active against α-glucosidase compared to the 3-fluoro- (**2b**, **2h**, **2n** and **2t**) and 4-(fluorophenyl)sulfonyl substituted analogues (**2c**, **2i**, **2o** and **2u**). The trend in activity for the 2-fluorophenylsulfonyl substituted derivatives is **2g** (**5-Cl**, IC_50_ = 4.2 ± 0.054 µM) > **2a** (**5-F**, IC_50_ = 5.6 ± 0.038 µM) > **2m** (**5-Br**, IC_50_ = 7.9 ± 0.044 µM) > **2s** (**5-Me**, IC_50_ = 18.1 ± 0.048 µM). The influence of *ortho*-fluorination on the geometry adopted by these compounds probably facilitated favourable interactions of the fluorophenyl ring with key amino acid residues in the active sites of α-glucosidase to lead to increased inhibitory activity. A combination of 5-Br and a 4-trifluoromethylsulfonyl or a 4-trifluoromethoxysulfonyl group in **2p** or **2q** resulted in relatively reduced activity against α-glucosidase compared to the analogues substituted on the acetophenone ring with 5-F (**2d** or **2e**), 5-Cl (**2j** or **2k**) or 5-CH_3_ group (**2v** or **2w**). Among the non-fluorinated analogues (Ar = -C_6_H_4_(4-OMe)), derivatives substituted with a hydrophobic 5-fluoro (**2f**) or hydrophobic 5-methyl group (**2x**) on the acetophenone ring exhibited strong activity against α-glucosidase (IC_50_ = 5.3 ± 0.159 and 3.6 ± 0.029 µM, respectively) compared to the 5-chloro **2l** (IC_50_ = 8.5 ± 0.043 µM) and 5-bromo **2r** (IC_50_ = 13.2 ± 0.043 µM) substituted analogues. A fluorine atom is known to increase the hydrophobicity of the compound [34], in turn, facilitates its the penetration into hydrophobic protein pockets ultimately increasing the activity of the drug [52]. Despite their strong inhibitory effect of the 4-methoxyphenylsulfonic esters **2f** and **2x** against α-glucosidase, these non-fluorinated analogues were not the most active within the corresponding sub-series **2a**–**f** and **2s**–**x**, respectively. It is envisaged that the π-electron rich 4-(methoxyphenyl) group on the sulfonyl arm augmented the hydrophobic properties of the 5-fluoro or 5-methyl substituent on the acetophenone scaffold resulting in increased (alkyl and/or π-alkyl) interactions with the residues in the active site of α-glucosidase and therefore increased inhibitory effect of **2f** and **2x**, respectively. The observed results in our view suggest that a fluorine atom at the *ortho* position or a fluorine-based alkyl or alkoxy group at the *para* position of the phenylsulfonyl moiety influenced the biological activity of the test compounds against α-glucosidase more so compared to the non-fluorinated phenylsulfonyl ring. The results of this assay identified derivatives **2a**, **2d**, **2e**, **2f**, **2g**, **2j**, **2k**, **2v**, **2w** and **2x** as the most active against α-glucosidase compared to acarbose. Compounds **2e** and **2v** are the strongest anti–α-glucosidase inhibitors among these sulfonic ester derivatives with comparable IC_50_ values of 3.1 ± 0.043 µM and 3.0 ± 0.014 µM, respectively. Strong α-glucosidase inhibitors are considered the most effective antihyperglycemic agents to reduce PPHG and are recommended as the first-line drugs in the management or treatment of T2DM [53]. Strong inhibition of α-glucosidase is required to prevent the breakdown of complex carbohydrates into glucose. However, selective inhibition of α-glucosidase should be avoided due to the localized action of this enzyme in the intestine, which may result in accumulated carbohydrates delivered to the colon for bacterial digestion resulting in gastrointestinal side effects [54]. It is envisaged that significantly reduced or no gastrointestinal side effects and therefore a better antihyperglycemic effect against T2DM will be achieved with antihyperglycemic drugs exhibiting dual activity against the carbohydrate hydrolyzing enzymes, but with moderate inhibitory effect against α-amylase. This is because partial inhibition of multiple targets will maintain a balance between the normal physiological functions of the protein targets, in turn, retard or prevent the progression of the disease compared to a complete inhibition of a single biochemical target [55]. With these considerations in mind, we selected compounds **2a**, **2d**, **2e**, **2f**, **2g**, **2j**, **2k**, **2v**, **2w** and **2x** with the highest inhibitory effect from each category and evaluated them further for anti–α-amylase activity as described in the next section.

#### 2.2.2. Inhibition of α-Amylase

Compounds **2a**, **2d**, **2e**, **2f**, **2g**, **2j**, **2k**, **2v**, **2w** and **2x** were assayed through an enzymatic assay in vitro using a BioVision’s α-Amylase Inhibitor Screening kit (Catalog # K482-100) against acarbose. The inhibition curves of these derivatives against α-amylase are included as Figure S3 of the Supplementary Materials and the corresponding IC_50_ values are also represented in Table 2. Compounds **2a**, **2g**, **2j**, **2k** and **2v** exhibited significant inhibition against this enzyme compared to acarbose (IC_50_ = 5.4 ± 0.131 µM) with the IC_50_ values of 7.9 ± 0.054, 7.0 ± 0.040, 7.3 ± 0.008, 9.8 ± 0.021 and 6.0 ± 0.020 µM, respectively. The selectivity index (SI) values (SI = IC_50_(α-glucosidase)/IC_50_(α-amylase) for the fluorinated derivatives **2a**, **2g**, **2j**, **2k** and **2v** are 0.71, 0.6, 0.74, 0.40 and 0.50, respectively. The strong α-glucosidase inhibitors **2e** (IC_50_ = 3.1 ± 0.043 µM) exhibited a moderate activity against this enzyme with an IC_50_ value of 15.2 ± 0.019 µM and SI value of 0.06. It is envisaged that an administration of this strong α-glucosidase inhibitor starting with low doses will help identify the minimum dose required for adequate glycemic control of the patient, in turn, reduce gastrointestinal side effects [56]. The 5-fluoro-2-(4-(methoxyphenyl)sulfonyl) substituted derivative **2f** exhibited the highest inhibitory activity against α-amylase (IC_50_ = 3.1 ± 0.110 µM) among the test compounds with an SI value of 1.71, which is higher than that of acarbose (SI = 1.19). Acarbose is a dual inhibitor of α-amylase and α-glucosidase, with strong inhibition against the former enzyme. Substantial inhibition of starch digestion due to the strong inhibitory effect of acarbose against α-amylase leads to carbohydrate accumulating in the intestine to be delivered in the colon resulting in undesirable gastrointestinal side effects from bacterial fermentation [53]. Since these effects are dose-dependent, initial administration with a low dose of **2f** and then gradually increasing the dose to the desired amount may ameliorate these side effects. The derivative **2x** substituted with a 5-methyl- and 4-(methoxyphenyl)sulfonyl group exhibited strong inhibition of α-glucosidase activity (IC_50_ = 3.6 ± 0.029 µM), but moderate inhibitory effect on α-amylase (IC_50_ = 11.9 ± 0.022 µM) with an SI value of 0.30. Better antihyperglycemic effect against T2DM will probably be achieved with the potential dual inhibitors of both enzymes, namely, derivatives **2a**, **2e**, **2g**, **2j**, **2k**, **2v** and **2x**. These compounds exhibited strong inhibitory effect against α-glucosidase compared to α-amylase.

#### 2.2.3. Inhibition of PTP1B

With the aim to develop arylsulfonic esters with potential to simultaneously target the carbohydrate hydrolyzing enzymes (α-glucosidase and/or α-amylase) and PTP1B activities, the derivatives **2a**, **2e**, **2f**, **2g**, **2j**, **2k**, **2v** and **2x** were further screened for inhibitory activity against this enzyme using the PTP1B Inhibitor Screening Assay Kit from Bioscience against sodium vanadate (Na_3_VO_4_) as reference standards for the assay (Figure S4 of the Supplementary Materials, Table 2). Sodium vanadate is a known non-selective, reversible and competitive inhibitor of protein tyrosine phosphatases, which is capable of normalizing blood glucose level in diabetes patients [57]. The presence of a fluorine atom at the 5-position of the acetophenone ring and *ortho*-position of the phenylsulfonyl group of **2a** resulted in strong inhibitory effect against PTP1B with an IC_50_ value of 6.3 ± 0.029 µM, which is equal to that of the reference standard (IC_50_ = 6.3 ± 0.050 µM). A significantly reduced inhibitory activity was observed for the 4-(trifluoromethoxyphenyl)sulfonyl derivative **2e** compared to the 4-(methoxyphenyl)sulfonyl substituted analogue **2f** with the IC_50_ values of 23.1 ± 0.006 µM and 5.9 ± 0.018 µM, respectively. The strong activity of **2f** is envisaged to be due to the increased electron density of the 4-(methoxyphenyl)sulfonyl ring, which would probably facilitate non-covalent interactions with the amino acid residues in the catalytic pocket and/or allosteric site of PTP1B. A chlorine atom on the C-5 position of the acetophenone ring resulted in moderate inhibitory effect of **2g**, **2j** and **2k** against PTP1B with IC_50_ values of 16.9 ± 0.016, 19.6 ± 0.042 and 20.1 ± 0.065 µM, respectively. Derivative **2v** substituted with a non-lipophilic methyl group on the acetophenone ring and a 4-(trifluoromethylphenyl)sulfonyl group exhibited significant inhibitory activity against PTP1B with an IC_50_ value of 7.6 ± 0.070 µM. A significantly reduced activity against this enzyme, on the other hand, was observed for the analogue **2x** (IC_50_ = 21.5 ± 0.066 µM) substituted with a 4-(methoxyphenyl)sulfonyl group. Although the fluorocarbon and hydrocarbon segments enhance the free energy of binding by a similar mechanism, molecular recognition become increased in the presence of fluoroalkylated groups compared to hydrophilic groups [34]. The 4-(trifluoromethylphenyl)sulfonyl substituted derivative **2v** will occupy a larger hydrophobic surface area compared to the strongly lipophilic 4-(methoxyphenyl)sulfonyl substituted derivative **2x**. Inhibition of PTP1B is envisaged to improve the sensitivity of IR, in turn, cure insulin resistance-related diseases. The fluorinated benzenesulfonic esters **2a** and **2v** exhibited strong activity against α-glucosidase compared to α-amylase and also strong or significant activity against PTP1B, respectively. These compounds will probably result in synergistic effects to prevent hyperglycemia and effectively improve insulin sensitivity by prolonging the phosphorylated state of the insulin receptor. The two compounds are potential effective therapeutics for the treatment of T2DM and obesity. Despite the strong activity of **2f** against PTP1B, this compound’s strong inhibition of α-amylase compared to α-glucosidase will probably lead to substantial inhibition of starch digestion resulting in undesirable gastrointestinal side effects from bacterial fermentation.

#### 2.2.4. Inhibition of VEGFR-2

Since VEGFR-2 has also been identified as a key biochemical target for the early diagnosis of diabetes retinopathy [12,13], we also evaluated compounds **2a**, **2e**, **2f**, **2g**, **2j**, **2k**, **2v** and **2x** through an in vitro enzymatic assay for potential to inhibit the activity of VEGFR-2. The inhibition curves of these derivatives against VEGFR-2 are included as Figure S5 of the Supplementary Materials and the corresponding IC_50_ values are also represented in Table 2 using nintedanib as a reference standard for the assay are also represented in Table 2. Nintedanib is a multiple receptor tyrosine kinase inhibitor capable to retard the activities of the fibroblast growth factor receptor-1 (FGFR-1), platelet derived growth factor receptor beta (PDGFRβ) and VEGFR-2 [58]. The test compounds exhibited moderate to weak inhibitory effect on the VEGFR-2 activity compared to nintedanib (IC_50_ = 1.4 ± 0.42 µM) with the IC_50_ values in the range 16.3 ± 0.29–34.4 ± 0.26 µM. The scaffolds of strong VEGFR-2 inhibitors comprise a flat hetero aromatic ring system to occupy the ATP (adenosine triphosphate) binding or hinge region, a hydrogen bond rich region with donor–acceptor pair (e.g., an amide or urea moiety) and the distal hydrophobic region [59]. The test compounds are substituted with hydrogen bond accepting moieties on the acetophenone ring for possible binding to the hinge region and an aryl sulfonyl ring as a distal hydrophobic tail to target the allosteric site of VEGFR-2. However, these compounds lack a hydrogen bond rich region with donor–acceptor pair present on the scaffolds of VEGFR-2 kinase inhibitors. The lack of this motif on the scaffolds of the sulfonic ester derivatives **2** probably accounts for the observed reduced inhibitory activity against this enzyme. Only compound **2v** substituted with a 5-methyl and a 4-(trifluoromethylphenyl)sulfonyl moiety as a distal hydrophobic tail exhibited moderate inhibitory activity against VEGFR-2 with an IC_50_ value of 16.3 ± 0.29 µM. The 4-trifluoromethylphenyl group probably resulted in increased hydrophilic (hydrogen and/or halogen bonding) and/or hydrophobic (eg, π-π stacking, π-π T shaped, π-alkyl, alkyl) interactions with the residues in the receptor to enhance the inhibitory effect of **2v** against VEGFR-2. Compound **2v** is a potential MTDL against T2DM capable of inhibiting both carbohydrates hydrolysing enzymes and PTP1B as well as VEGFR-2 activities.

#### 2.2.5. Cytotoxicity of Compounds **2a**, **2e**, **2f**, **2g**, **2j**, **2k**, **2v** and **2x**

The multifactorial origin of T2DM and its link to cancer prompted us to screen compounds **2a**, **2e**, **2f**, **2g**, **2j**, **2k**, **2v** and **2x** for antiproliferative effect against the MCF-7 and A549 cell lines to establish their anticancer properties (Table 3). The corresponding inhibition curves of these derivatives against the MCF-7 and A549 cell lines are included as Figures S6 and S7 of the Supplementary Materials. Compounds **2a**, **2f** and **2j** exhibited moderate effect against the MCF-7 cell viability compared to the anticancer drugs, doxorubicin (IC_50_ = 0.59 ± 0.03 µM) and nintedanib (IC_50_ = 0.40 ± 0.03 µM) with IC_50_ values of 4.96 ± 0.05 µM, 4.38 ± 0.07 µM and 2.58 ± 0.02 µM, respectively. The analogues **2f**, **2j**, **2k** and **2x** exhibited significant toxicity to the A549 cells compared to doxorubicin (IC_50_ = 0.71 ± 0.09 µM) and nintedanib (IC_50_ = 0.78 ± 0.04 µM) with the IC_50_ values of 1.91 ± 0.01, 2.52 ± 0.02, 1.89 ± 0.05, and 1.01 ± 0.01 µM, respectively. A stable long-term use of MTDLs with anticancer properties for the treatment of T2DM will require drugs to exert potency against the cancer cells and bot affect viability of the normal cells. As a result, the test compounds were assayed for cytotoxicity on the Vero cells to establish their selectivity (Table 3, Figure S8). The anticancer drugs, doxorubicin and nintedanib, were toxic to the Vero cells with the IC_50_ values of 0.94 ± 0.04 and 0.24 ± 0.02 µM, respectively. A significantly reduced toxicity to the Vero cells was observed for the test compounds with high IC_50_ values in the range of 20.78 ± 0.14–56.77 ± 0.18 µM.

The experimental and in silico studies have demonstrated that the noncovalent interactions such as hydrogen and/or halogen bonds and hydrophobic (eg, π-π stacking, π-π T shaped, π-alkyl, alkyl) interactions, π-charged (anion or cation) interactions and/or salt bridges formed between the ligands and amino acid residues help to embed the ligand within the active site of the receptors to enhance or inhibit biological activity [39,43]. To augment the results from the in vitro studies, we performed molecular docking on the most active derivatives against the carbohydrate hydrolyzing enzymes, PTP1B and VEGFR-2 to reveal the site of action and possible modes of interaction with the key amino acid residues of the receptors.

### 2.3. Molecular Docking Studies on ***2a***, ***2e***, ***2f***, ***2g***, ***2j***, ***2k***, ***2v*** and ***2x***

#### 2.3.1. Molecular Docking of **2a**, **2e**, **2f**, **2g**, **2j**, **2k**, **2v** and **2x** into α-Glucosidase Active Site

**Figure 3:**
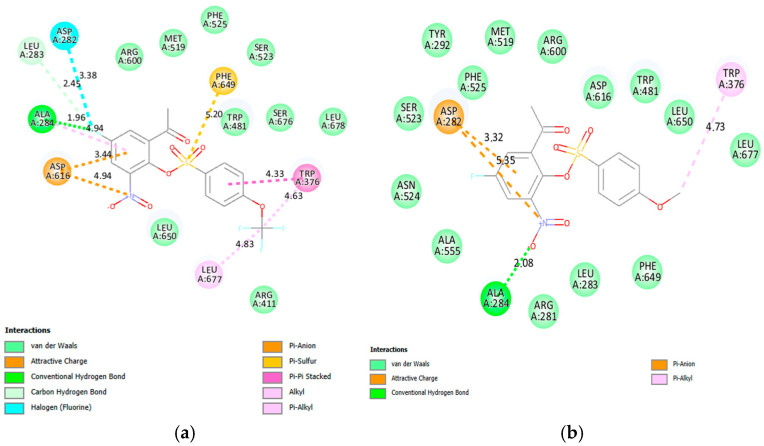
The interactions of **2e** (**a**) and **2f** (**b**) with the human lysosomal acid-α-glucosidase (PDB code 5NN8). The poses also show the bonding interaction distances and colour codes for various interactions.

The lack of the elucidated X-ray crystallographic structures for the free or inhibitor-bound yeast α-glucosidases led us to dock the test compounds into the active sites of the human lysosomal acid-α-glucosidase (PDB code 5NN8) [60] co-crystallized with acarbose. The acidic residues such as Asp282, Asp404, Asp518, Asp616, and Asp645, and the basic residues, Arg600, Arg672, and His674 comprise the catalytic site of the lysosomal human α-glucosidase enzyme [60]. Acarbose was re-docked into α-glucosidase and its hydroxyl groups formed a network of hydrogen bonds with the amino acid residues, Arg411, Arg600, Asp404, Asp616 and His674 as shown in Figure S9 of the Supplementary Materials. Attractive charge interactions exist between acarbose and the residues Asp282 and Asp518 as well as a salt bridge with Asp616. Several of the test compounds are predicted to bind with one or two of the residues. The docking poses of acarbose and compounds **2a**, **2e**, **2f**, **2g**, **2j**, **2k**, **2v** and **2x** are included in Figure S9 of the Supplementary Materials and only the poses of derivatives **2e** and **2f** are included herein as Figure 3. The docking pose of **2e** (Figure 3a) revealed halogen and hydrogen bonding interactions of the 5-fluoro atom with the residues Asp282 and Ala284, respectively. There is a weak carbon hydrogen bond between fluorine and Leu283. Hydrophilic (hydrogen and halogen bonds) interactions helped to embed this ligand within the active site of π-glucosidase to inhibit enzyme activity. The 4-(methoxyphenylsulfonyl) analogue **2f** (Figure 3b) only forms a single hydrogen bond with Ala284 involving the negatively charged oxygen of the nitro group. These in silico results in our view corroborate their in vitro inhibitory activity against α-glucosidase.

#### 2.3.2. Molecular Docking of **2a**, **2e**, **2f**, **2g**, **2j**, **2k**, **2v** and **2x** into α-Amylase Active Site

**Figure 4:**
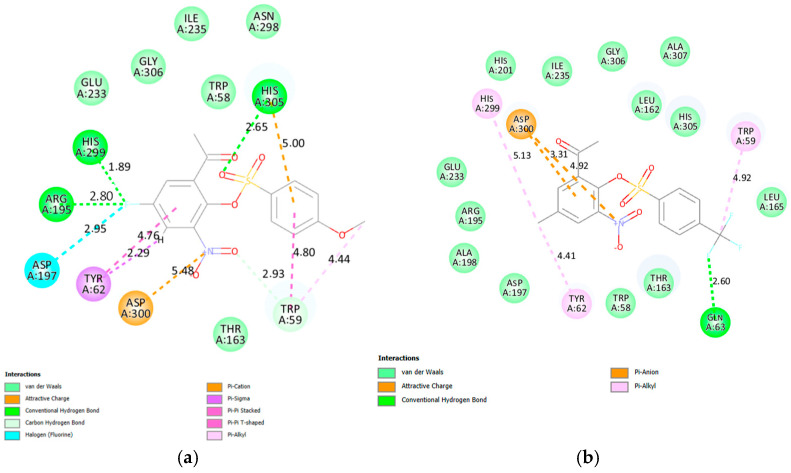
The interactions of **2f** (**a**) and **2v** (**b**) with human pancreatic α-amylase (PDB code 3BAJ).

Compounds **2a**, **2e**, **2f**, **2g**, **2j**, **2k**, **2v** and **2x** as well as acarbose were docked into the active site of α-amylase (PDB code 3BAJ) and their corresponding poses are included in Figure S10 of the Supplementary Materials. The docked structure of acarbose shown in Figure S10 shows the hydroxyl groups engaged in several hydrogen bonding interactions with the residues i.e., Tyr62, Asp197, Glu233 and Asp300 (two) in the active site of α-amylase, which are involved in the hydrolysis of starch. Acarbose also participates in a π-alkyl interactions with Trp59 and His305 as well as π-donor hydrogen bond interaction with Asp300. The residues such as Arg195, Asn298, His299, Asp300, His305, Ala307 and Asp356 are responsible for the hydrolysis of starch [61]. The docking poses of derivatives **2f** and **2v** are included as Figure 4. The 5-fluorine atom of **2f** (Figure 4a) is engaged in two hydrogen bonding interactions with His299 and Arg195 as well as a halogen bonding interaction with Asp197. His305 is involved in hydrogen bonding interaction with one of the oxygen atoms of the sulfonyl moiety. The hydrogen atom of the acetophenone ring *ortho* to the nitro group of this compound participates in a π-sigma interactions with Tyr62. The residue Thr163 forms a weak carbon hydrogen bond with the uncharged oxygen atom of the nitro group. Hydrophilic (hydrogen and halogen bond) interactions involve the 5-fluoro-3-nitroacetophenonesulfonic ester framework and not the 4-methoxyphenyl group. The observed increased inhibitory effect seems to be influenced by the hydrophilic interactions of the 5-fluoro-3-nitroacetophenone with the residues in the active site of this enzyme. The fluorine atom of the trifluoromethyl group of **2v** forms a hydrogen bond with the residue Trp59. The strong electron-withdrawing inductive and resonance effects of the nitro group is more likely to polarize these molecules to enable the acetophenone ring to engage in electrostatic interactions with the amino acid residues of these carbohydrate hydrolyzing enzymes consistent with the design strategy.

#### 2.3.3. Molecular Docking of **2a**, **2e**, **2f**, **2g**, **2j**, **2k**, **2v** and **2x** into PTP1B

**Figure 5:**
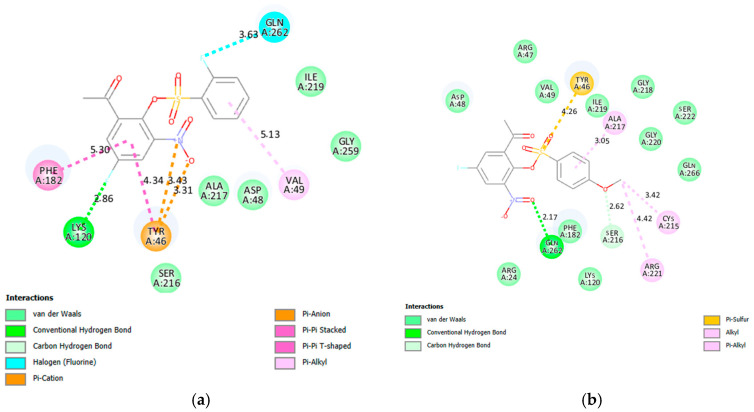
The interactions of **2a** (**a**) and **2f** (**b**) with PTP1B catalytic (PDB 2QBP).

**Figure 6:**
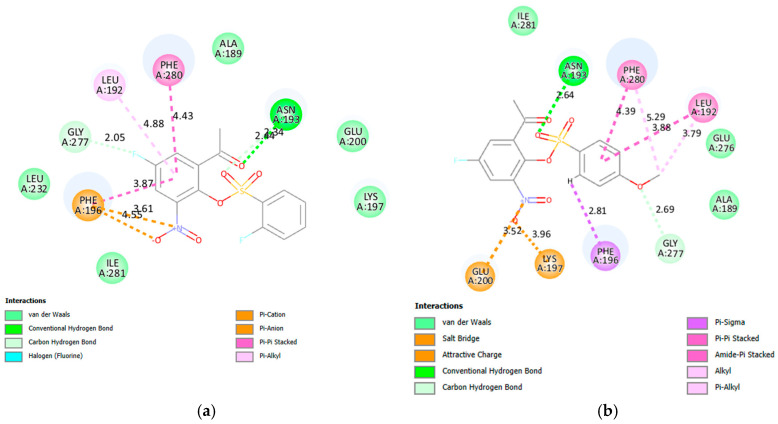
The interactions of **2a** (**a**) and **2f** (**b**) with PTP1B allosteric site (PDB 1T49).

PTP1B inhibitors may bind to the active site and/or to the allosteric site of this enzyme to inhibit its activity. It has been observed that the inhibitors that target the catalytic domain of this enzyme result in the inhibition of other PTP family members resulting in undesirable side effects [62]. Targeting the less conserved allosteric site and side pockets present at the borders of the catalytic site has been found to circumvent these challenges and reduce side effects [62]. Compounds **2a**, **2e**, **2f**, **2g**, **2j**, **2k**, **2v** and **2x** were docked into the catalytic (PDB 2QBP) and allosteric (PDB 1T49) sites and their docking poses are included in Figures S11 and S12 of the Supplementary Materials, respectively. The docking poses of **2a** and **2f** in the catalytic and allosteric sites are represented in Figure 5 and Figure 6, respectively.

(i) Docking into the Catalytic (PDB 2QBP) Site

The native ligand forms four hydrogen bonds with the residues Ala217 (2), Arg221 and Ser216 which help embed this ligand in the active site (Figure 5). Additionally, the ligand forms π-π stacked interaction with Phe182 and two π-π T-shaped interactions with Tyr46. The residues Arg221 and Phe182 are also involved in attractive charge and π-sulfur interactions with the ligand. The fluorine atoms at the C-5 position of the acetophenone ring of **2a** (Figure 5a) and one at the *ortho* position of the phenylsulfonyl group participate in hydrogen and halogen bonding interactions with Lys120 and Gln262, respectively. Hydrogen and halogen bonding interactions probably increased the residence time of this compound in the active site of PTP1B increasing the inhibition of this enzyme’s activity. A hydrogen bonding interaction is predicted between the uncharged oxygen atom of the nitro group of **2f** and the residue Gln262 (Figure 5b). The oxygen atom of the methoxy group forms a weak carbon hydrogen bond with Ser216. Hydrogen bonding interaction with Gln262 and the weak carbon hydrogen bond formed with Ser216 probably increased the activity of **2f** against PTP1B.

(ii) Docking into the Allosteric (PDB 1T49) Site

Allosteric inhibitors tend to be accommodated in the α3-α6-α7 helices of PTP1B to destabilize the network of hydrogen bonds, which are essential for the closure of the WPD loop, in turn, prohibit its closure [57,62]. Two hydrogen bonds are formed between the native ligand and the residues Leu192 and Asn193 in the allosteric site of PTP1B The π-cation and π-lone pair contacts with Lys197 and Phe196, respectively, as well as the hydrophobic (π-alkyl and π-π T-shaped) interactions with Phe280 also help to embed this ligand in the allosteric site of this enzyme. A halogen bond is predicted between fluorine atom of **2a** (Figure 6a) and the residue Glu277. The carbonyl oxygen and one of the oxygen atoms of the sulfonyl moiety of this compound form a conventional hydrogen bond and a weak carbon hydrogen bond with Asn193, respectively. The increased hydrophobic interactions with amino acid residues in the allosteric site predicted for this compound are consistent with its increased inhibitory activity against PTP1B. A conventional hydrogen bond is formed between an oxygen atom of the sulfonyl group of **2f** (Figure 6b) and Asn193. It is envisaged that the formation of a hydrogen bond with Asn193 in the helices of α3–α6 will restrict the WPD loop to its open conformation and inactivate PTP1B. These compounds are predicted to target strongly the allosteric site and some protein residues on the side pockets at the borders of the catalytic site, which make them potential PTP1B inhibitors with reduced side effects.

#### 2.3.4. Docking into VEGFR-2 Catalytic Site (PDB 4ASD)

**Figure 7:**
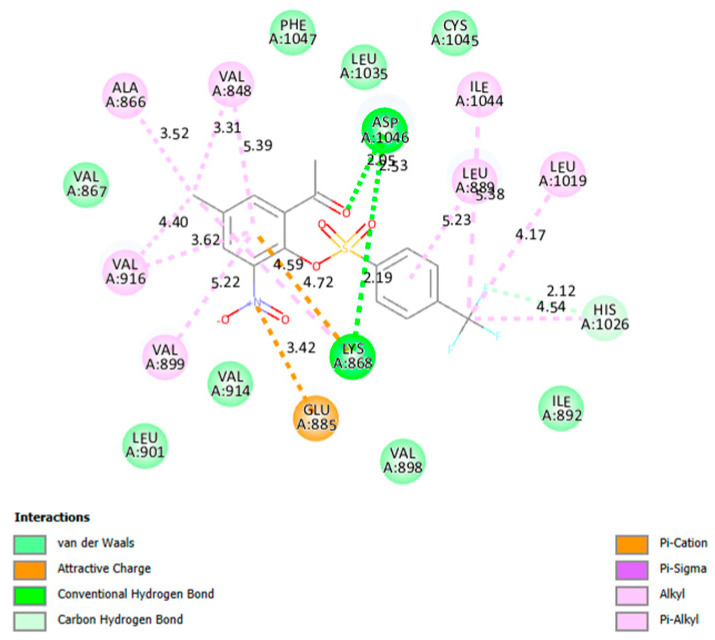
The interactions of **2v** with VEGFR-2 active site.

Compounds **2a**, **2e**, **2f**, **2g**, **2j**, **2k**, **2v** and **2x** and sorafenib were docked into the active site of VEGFR-2 (PDB 4ASD) and their poses are enclosed as Figure S13 and only the docking pose of **2v** is represented in Figure 7. The native ligand, sorafenib, docked into the active site of VEGFR-2 to engage in four hydrogen bonding interactions with Cys919, Asp1046 and Glu885 (2) as well as halogen bonding interaction with Ile1044. There are eleven π-alkyl interactions between this ligand and the residues in the active site of this enzyme including a π-π T-shaped and alkyl interactions with Phe1047 and Leu840, respectively. The hydrophobic interactions of Sorafenib with Leu889 and Leu840 and hydrophilic interactions with Glu885 and Cys919 in the active pocket of VEGFR-2 are essential to inhibit the enzyme’s catalytic activity. Compound **2v** forms two hydrogen bonds involving the residue Asp1046 and the carbonyl oxygen and one of the oxygen atoms of the sulfonyl group. This oxygen atom of the sulfonyl group is also involved in another hydrogen bonding interaction with Lys868. One of the fluorine atoms of the trifluoromethyl group forms a weak carbon hydrogen bond with His1026. The nitrogen atom of this compound is involved in an attractive charge interaction with the residue Glu885 in the active pocket of VEGFR-2. Despite these interactions, this compound is not involved in π-π T-shaped or stacking interactions, which probably account for its moderate activity against this enzyme.

### 2.4. Pharmacokinetics Properties Prediction of ***2a***, ***2e***, ***2f***, ***2g***, ***2j***, ***2k***, ***2v*** and ***2x***

The in silico drug-likeness features play an important role in assessing the quality of compounds to narrow down the list of candidates for future in vivo studies and/or preclinical testing. Additionally, ADME properties of the sulfonic esters **2a**, **2e**, **2f**, **2g**, **2j**, **2k**, **2v** and **2x** were calculated using the SwissADME (version 2024) [63] to predict their drug-likeness. The Lipinski rule of five-hydrogen bond donors ≤ 5, hydrogen bond acceptors ≤ 10, molecular mass < 500 Da and clogP < 5) was applied in the calculation [64]. This rule describes the relationship between physicochemical and pharmacokinetic properties of drugs. The corresponding data for pharmacokinetic and drug-likeness are shown in Table 4. The lipophilicity (XLogP3) values between −0.7 and 5 for the tested compounds are considered to be within the optimal limits by the bioavailability radar displayed by SwissADME. The molecular weight and the TPSA values for all the test compounds are within the optimal ranges of 150 to 500 g/mol and between 20 and 130 Å^2^, respectively. No compound had more than 7 rotatable bonds, with nine (9) rotatable bonds being the cut-off limit. Aspects of how the compounds would behave as drugs in the body are considered in terms of solubility and absorption in the gastrointestinal (GI) tract as well as the drug’s metabolism and excretion via the cytochrome P450 enzymes. Compounds **2a**, **2f**, **2g** and **2x** displayed high GI tract absorption values while **2e**, **2j**, **2k**, **2v** had low GI track absorption. Compounds **2a** and **2f** are predicted to be more soluble while all the other derivatives are moderately soluble. The cytochrome P450 enzymes act to clear drugs from the body, all the compounds displayed inhibition of most of the isoforms for this enzyme class. The calculated data for the test compounds did not violate the Lipinski’s rule of five.

## 3. Materials and Methods

### 3.1. Materials and Instrumentation

The chemicals and solvents used in this study were purchased from Merck Ltd. (Modderfontein, Johannesburg, South Africa) and were used without further purification. The melting point (mp.) values of the prepared compounds were recorded on a Stuart SMP5 melting point apparatus (Coleparmer, Stone, Staffordshire, UK). The FT-IR spectra were recorded on a Bruker VERTEX 70 FT-IR Spectrometer (Bruker Optics, Billerica, MA, USA) equipped with an ATR (diamond attenuated total reflectance) accessory. The ^1^H- and ^13^C NMR spectra, on the other hand, were measured in deuterated chloroform (CDCl_3_) at 500 MHz and 125 MHz, respectively, using Agilent 500 MHz NMR spectrometer (Agilent Technologies, Oxford, UK). The chemical shifts are quoted relative to tetramethylsilane (TMS) used as an internal reference standard (δ = 0.00 ppm) or to the residual protonated solvent peak in CDCl_3_ (δ_H_ = 7.26 ppm) and δ_C_ = 77.0 ppm. The ^19^F NMR spectra were recorded at 470 MHz and the chemical shift values are reported in parts per million (ppm) relative to CCl_3_F as an external standard.

### 3.2. Typical Procedure for the Synthesis of ***2a***–***x***

A mixture of **1** (1 equiv.) and triethylamine (2 equiv.) in dichloromethane (2.5 mL/mmol of **1**) at room temperature (RT) was treated with a benzenesulfonyl chloride derivative (1.1. equiv.). The mixture was stirred at RT for 3 h and then quenched with an ice-cold water. The mixture was extracted with dichloromethane and the combined organic phases were washed with aqueous sodium hydrogen carbonate followed by brine. The organic phase was dried over anhydrous MgSO_4_ and the salt was filtered off. The solvent was evaporated under reduced pressure on a rotary evaporator and the crude product was recrystallized from ethanol. Compounds **2a**–**x** were prepared in this manner.

#### 3.2.1. 2-Acetyl-4-fluoro-6-nitrophenyl 2-fluorobenzenesulfonate (**2a**)

Brown solid (1.74 g, 97%), mp. 108–110 °C; ν_max_ (ATR) 511, 565, 716, 756, 844, 1076, 1161, 1290, 1354, 1477, 1537, 1705, 3094 cm^−1^; ^1^H NMR: δ_H_ 2.64 (s, 3H, -CH_3_), 7.27–7.36 (m, 2H, H-3′, H-4′), 7.59 (dd, *J* = 7.4 and 3.2 Hz, 1H, H-3), 7.73–7.80 (m, 3H, H-5, H-5′, H-6′); ^13^C NMR: δ 30.0, 116.0 (d, ^2^*J*_CF_ = 27.9 Hz), 118.0 (d, ^2^*J*_CF_ = 20.6 Hz), 121.1 (d, ^2^*J*_CF_ = 24.1 Hz), 122.3 (d, ^2^*J*_CF_ = 13.1 Hz), 125.0 (d, ^4^*J*_CF_ = 4.0 Hz), 131.1, 134.5 (d, ^4^*J*_CF_ = 4.1 Hz), 138.2 (d, ^3^*J*_CF_ = 8.9 Hz), 139.2 (d, ^3^*J*_CF_ = 6.2 Hz), 144.7 (d, ^3^*J*_CF_ = 8.1 Hz), 159.5 (d, ^1^*J*_CF_ = 255.8 Hz), 159.7 (d, ^1^*J*_CF_ = 262.2 Hz), 195.1; ^19^F NMR: δ −105.4, −107.5.

#### 3.2.2. 2-Acetyl-4-fluoro-6-nitrophenyl 3-fluorobenzenesulfonate (**2b**)

Yellow solid (1.56 g, 87%), mp. 108–110 °C; ν_max_ (ATR) 501, 577, 679, 706, 714, 772, 835, 889, 1084, 1167, 1286, 1354, 1388, 1462, 1537, 1701, 3075 cm^−1^; ^1^H NMR: δ 2.61 (s, 3H, -CH_3_), 7.46–7.50 (m, 1H, H-4′), 7.54–7.57 (m, 1H, H-5′), 7.58–7.64 (overlapping signals, 2H, H-2′, H-3), 7.65–7.67 (m, 1H, H-6′), 7.76 (dd, *J* = 6.8, 3.2 Hz, 1H, H-5); ^13^C NMR: δ 29.9, 116.07 (d, ^2^*J*_CF_ = 28.0 Hz), 116.14 (d, ^2^*J*_CF_ = 25.3 Hz), 121.2 (d, ^2^*J*_CF_ = 24.1 Hz), 123.0 (d, ^2^*J*_CF_ = 21.1 Hz), 124.7 (d, ^4^*J*_CF_ = 3.5 Hz), 131.7 (d, ^3^*J*_CF_ = 7.8 Hz), 134.2 (d, ^4^*J*_CF_ = 4.2 Hz), 135.2 (d, ^3^*J*_CF_ = 7.4 Hz), 139.1 (d, ^3^*J*_CF_ = 6.2 Hz), 144.9 (d, ^3^*J*_CF_ = 8.1 Hz), 159.6 (d, ^1^*J*_CF_ = 256.2 Hz), 162.4 (d, ^1^*J*_CF_ = 254.3 Hz), 194.8; ^19^F NMR: δ −107.3, −107.5.

#### 3.2.3. 2-Acetyl-4-fluoro-6-nitrophenyl 4-fluorobenzenesulfonate (**2c**)

Brown solid (1.67 g, 93%), mp. 106–108 °C; ν_max_ (ATR) 496, 501, 576, 704, 707, 771, 835, 842, 1169, 1286, 1354, 1388, 1462, 1537, 1701, 3084 cm^−1^; ^1^H NMR: δ 2.61 (s, 3H, CH_3_), 7.27 (t, *J* = 8.4 Hz, 2H, H-3′,5′), 7.62 (dd, *J* = 3.2 and 7.0 Hz, 1H, H-3), 7.75 (dd, *J* = 3.2 and 7.0 Hz, 1H, H-5), 7.86 (dd, *J* = 4.7 and 8.9 Hz, 2H, H-2′,6′); ^13^C NMR: δ 30.0, 116.1 (d, ^2^*J*_CF_ = 28.0 Hz), 117.4 (d, ^2^*J*_CF_ = 23.0 Hz), 121.3 (d, ^2^*J*_CF_ = 24.0 Hz), 129.1 (d, ^4^*J*_CF_ = 3.2 Hz), 131.9 (d, ^3^*J*_CF_ = 10.1 Hz), 134.3 (d, ^4^*J*_CF_ = 4.1 Hz), 139.1 (d, ^3^*J*_CF_ = 6.2 Hz), 144.9 (d, ^3^*J*_CF_ = 8.1 Hz), 159.5 (d, ^1^*J*_CF_ = 255.8 Hz), 166.9 (d, ^1^*J*_CF_ = 260.3 Hz), 195.1; ^19^F NMR: δ −99.0 (s), −107.4.

#### 3.2.4. 2-Acetyl-4-fluoro-6-nitrophenyl 4-(trifluoromethyl)benzenesulfonate (**2d**)

Orange solid (1.88 g, 92%), mp. 89–91 °C; ν_max_ (ATR) 525, 590, 718, 779, 843, 1063, 1124, 1321, 1396, 1537, 1701, 3084 cm^−1^; ^1^H NMR: δ 2.63 (s, 3H, -CH_3_), 7.63 (dd, *J* = 3.3 and 7.1 Hz, 1H, H-3), 7.76 (dd, *J* = 3.3 and 7.1 Hz, 1H, H-5), 7.88 (d, *J* = 7.6 Hz, 2H, H-3′,5′), 8.03 (d, *J* = 7.6 Hz, 2H, H-2′,6′); ^13^C NMR: δ 29.9, 116.1 (d, ^2^*J*_CF_ = 28.0 Hz), 121.2 (d, ^2^*J*_CF_ = 24.0 Hz), 122.8 (q, ^1^*J*_CF_ = 273.5 Hz), 126.9 (q, ^3^*J*_CF_ = 3.7 Hz), 129.4, 133.8 (d, ^4^*J*_CF_ = 4.2 Hz), 137.0, 137.1 (q, ^2^*J*_CF_ = 33.5 Hz), 139.2 (d, ^3^*J*_CF_ = 6.2 Hz), 144.9 (d, ^3^*J*_CF_ = 8.0 Hz), 159.7 (d, ^1^*J*_CF_ = 256.4 Hz), 194.7; ^19^F NMR: δ −63.6, −107.1.

#### 3.2.5. 2-Acetyl-4-fluoro-6-nitrophenyl 4-(trifluoromethoxy)benzenesulfonate (**2e**)

Yellow solid (1.82 g, 89%), mp. > 350 °C; ν_max_ (ATR) 525, 592, 677, 714, 777, 833, 1086, 1163, 1184, 1387, 1537, 1699, 3046 cm^−1^; ^1^H NMR (500 MHz, CDCl_3_): δ 2.62 (s, 3H, -CH_3_), 7.41 (d, *J* = 8.9 Hz, 2H, H-3′,5′), 7.63 (dd, *J* = 3.2 and 7.1 Hz, 1H, H-3), 7.75 (dd, *J* = 3.2 and 7.1 Hz, 1H, H-5), 7.92 (d, *J* = 8.9 Hz, 2H, H-2′,6′); ^13^C NMR (125 MHz, CDCl_3_): δ 29.9, 116.0 (d, ^2^*J*_CF_ = 27.9 Hz), 120.1 (q, ^1^*J*_CF_ = 260.9 Hz), 121.22 (d, ^2^*J*_CF_ = 24.1 Hz), 121.23 (q, ^4^*J*_CF_ = 1.1 Hz), 131.2, 131.3, 134.1 (d, ^4^*J*_CF_ = 4.0 Hz), 139.2 (d, ^3^*J*_CF_ = 6.2 Hz), 144.9 (d, ^3^*J*_CF_ = 8.1 Hz), 154.4 (q, ^3^*J*_CF_ = 1.8 Hz), 159.6 (d, ^1^*J*_CF_ = 256.1 Hz), 194.8; ^19^F NMR: δ −57.7, −107.3.

#### 3.2.6. 2-Acetyl-4-chloro-6-nitrophenyl 4-methoxybenzenesulfonate (**2f**)

Grey solid (1.56 g, 84%), mp. 105–107 °C; ν_max_ (ATR) 526, 543, 567, 646, 688, 779, 804, 889, 1010, 1085, 1161, 1215, 1271, 1348, 1452, 1541, 1589, 1701, 3086 cm^−1^; ^1^H NMR: δ 2.61 (s, 3H, -CH_3_), 3.91 (s, 3H, -OCH_3_), 7.01 (d, *J* = 9.0 Hz, 2H, H-3′,5′), 7.61 (dd, *J* = 3.2 and 7.5 Hz, 1H, H-3), 7.70–7.73 (overlapping signals, 3H, H-2′,6′ an H-5); ^13^C NMR: δ 29.9, 55.9, 115.0, 116.0 (d, ^2^*J*_CF_ = 28.0 Hz), 121.2 (d, ^2^*J*_CF_ = 23.9 Hz), 124.0, 131.2, 134.9 (d, ^4^*J*_CF_ = 4.0 Hz), 139.2 (d, ^3^*J*_CF_ = 6.1 Hz), 145.1 (d, ^3^*J*_CF_ = 8.0 Hz), 159.3 (d, ^1^*J*_CF_ = 255.2 Hz), 165.4, 195.0; ^19^F NMR: δ −108.2.

#### 3.2.7. 2-Acetyl-4-chloro-6-nitrophenyl 2-fluorobenzenesulfonate (**2g**)

Yellow solid (1.65 g, 95%), mp. 131–133 °C; ν_max_ (ATR) 519, 567, 708, 768, 884, 1076, 1178, 1251, 1352, 1477, 1541, 1705, 3084 cm^−1^; ^1^H NMR: δ 2.63 (s, 3H, -CH_3_), 7.28–7.36 (m, 2H, H-3′ and H-4′), 7.74–7.80 (m, 2H, H-5′ and H-6′), 7.82 (d, *J* = 2.7 Hz, 1H, H-3), 7.99 (d, *J* = 2.7 Hz, 1H, H-5); ^13^C NMR: δ 30.1, 118.0 (d, ^2^*J*_CF_ = 20.6 Hz), 122.3 (d, ^2^*J*_CF_ = 13.1 Hz), 125.0 (d, ^4^*J*_CF_ = 4.0 Hz), 128.2, 131.1, 133.9, 134.0, 136.8, 138.3 (d, ^3^*J*_CF_ = 8.9 Hz), 138.6, 144.4, 159.7 (d, ^1^*J*_CF_ = 262.3 Hz), 195.2; ^19^F NMR: δ −105.3.

#### 3.2.8. 2-Acetyl-4-chloro-6-nitrophenyl 3-fluorobenzenesulfonate (**2h**)

Light brown solid (1.56 g, 90%), mp. 106–108 °C; ν_max_ (ATR) 486, 574, 673, 680, 779, 858, 1086, 1168, 1175, 1348, 1541, 1705, 3069 cm^−1^; ^1^H NMR (500 MHz, CDCl_3_): δ 2.60 (s, 3H, -CH_3_), 7.47–7.51 (m, 1H, H-4′), 7.54–7.56 (m, 1H, H-5′), 7.60 (td, *J* = 5.0 and 8.0 Hz, 1H, H-2′), 7.63–7.67 (m, 1H, H-6′), 7.85 (d, *J* = 2.6 Hz, 1H, H-3), 8.00 (d, *J* = 2.6 Hz, 1H, H-5); ^13^C NMR (125 MHz, CDCl_3_): δ 30.0, 116.1 (d, ^2^*J*_CF_ = 25.3 Hz), 123.1 (d, ^2^*J*_CF_ = 21.1 Hz), 124.7 (d, ^4^*J*_CF_ = 3.5 Hz), 128.4, 131.8 (d, ^3^*J*_CF_ = 7.8 Hz), 134.1, 135.1 (d, ^3^*J*_CF_ = 7.5 Hz), 136.5, 138.5, 144.5, 162.4 (d, ^1^*J*_CF_ = 254.0 Hz), 195.0; ^19^F NMR (470 MHz, CDCl_3_): δ −107.4.

#### 3.2.9. 2-Acetyl-4-chloro-6-nitrophenyl 4-fluorobenzenesulfonate (**2i**)

Pale yellow solid (1.68 g, 97%), mp. 129–131 °C; ν_max_ (ATR) 489, 538, 584, 690, 688, 746, 796, 840, 1088, 1155, 1175, 1206, 1340, 1390, 1485, 1531, 1537, 1533, 1707, 3078 cm^−1^; ^1^H NMR: δ 2.61 (s, 3H, CH_3_), 7.27 (t, *J* = 8.7 Hz, 2H, H-3′,5′), 7.85 (d, *J* = 2.7 Hz, 1H, H-3), 7.87 (dd, *J* = 4.0 and 8.7 Hz, 2H, H-2′,6′), 7.98 (d, *J* = 2.7 Hz, 1H, H-5); ^13^C NMR: δ 30.0, 117.3 (d, ^2^*J*_CF_ = 23.0 Hz), 128.2, 129.3 (d, ^4^*J*_CF_ = 3.3 Hz), 131.9 (d, ^3^*J*_CF_ = 10.1 Hz), 133.9, 134.0, 136.6, 138.6, 144.7, 167.0 (d, ^1^*J*_CF_ = 260.3 Hz), 195.0; ^19^F NMR: δ −99.0.

#### 3.2.10. 2-Acetyl-4-chloro-6-nitrophenyl 4-(trifluoromethyl)benzenesulfonate (**2j**)

Orange solid (1.59 g, 81%), mp. 141–143 °C; ν_max_ (ATR) 532, 577, 592, 686, 716, 808, 846, 1061, 1123, 1323, 1393, 1526, 1701, 3076 cm^−1^; ^1^H NMR: δ 2.62 (s, 3H, -CH_3_), 7.88 (d, *J* = 8.6 Hz, 3H, H-3′,5′ and H-5), 8.00–8.04 (m, 3H, H-2′,6′ and H-3); ^13^C NMR: δ 30.0, 122.8 (q, ^1^*J*_CF_ = 273.4 Hz), 126.9 (q, ^3^*J*_CF_ = 3.7 Hz), 128.3, 129.4, 134.0, 134.2, 136.1, 137.0 (q, ^2^*J*_CF_ = 33.5 Hz), 137.1, 138.5, 144.6, 194.5; ^19^F NMR: δ −63.4.

#### 3.2.11. 2-Acetyl-4-chloro-6-nitrophenyl 4-(trifluoromethoxy)benzenesulfonate (**2k**)

Yellow solid (1.82 g, 89%), mp. 124–126 °C; ν_max_ (ATR) 563, 592, 662, 775, 858, 1084, 1157, 1177, 1346, 1537, 1697, 3070 cm^−1^; ^1^H NMR: δ 2.62 (s, 3H, -CH_3_), 7.41 (d, *J* = 8.9 Hz, 2H, H-3′,5′), 7.86 (d, *J* = 2.6 Hz, 1H, H-3), 7.90–7.94 (m, 2H, H-2′,6′), 7.99 (d, *J* = 2.6 Hz, 1H, H-5); ^13^C NMR: δ 29.9, 120.1 (q, ^1^*J*_CF_ = 260.8 Hz), 121.2 (q, ^4^*J*_CF_ = 1.2 Hz), 128.2, 131.2, 131.4, 134.0, 134.1, 136.4, 138.6, 144.7, 154.4 (q, ^3^*J*_CF_ = 1.8 Hz), 194.9; ^19^F NMR: δ −57.7.

#### 3.2.12. 2-Acetyl-4-chloro-6-nitrophenyl 4-methoxybenzenesulfonate (**2l**)

Grey solid (1.70 g, 95%), mp. 97–99 °C; ν_max_ (ATR) 534, 588, 665, 723, 772, 844, 1016, 1082, 1166, 1263, 1350, 1499, 1541, 1697, 2981, 3076 cm^−1^; ^1^H NMR: δ 2.61 (s, 3H, -CH_3_), 3.92 (s, 3H, -OCH_3_), 7.01 (d, *J* = 8.8 Hz, 2H, H-3′,5′), 7.72 (d, *J* = 8.8 Hz, 2H, H-2′,6′), 7.84 (d, *J* = 2.3 Hz, 1H, H-3), 7.96 (d, *J* = 2.3 Hz, 1H, H-5); ^13^C NMR: δ 30.0, 56.0, 115.0, 124.0, 128.2, 131.2, 133.5, 134.0, 137.2, 138.6, 144.9, 165.4, 195.1.

#### 3.2.13. 2-Acetyl-4-bromo-6-nitrophenyl 2-fluorobenzenesulfonate (**2m**)

Yellow solid (1.48 g, 92%), mp. 125–127 °C; ν_max_ (ATR) 517, 567, 743, 768, 797, 858, 1076, 1179, 1256, 1350, 1477, 1537, 1705, 3078 cm^−1^; ^1^H NMR: δ 2.63 (s, 3H, -CH_3_), 7.27–7.36 (m, 2H, H-3′ and H-4′), 7.74–7.81 (m, 2H, H-5′ and H-6′), 7.97 (d, *J* = 2.5 Hz, 1H, H-3), 8.13 (d, *J* = 2.5 Hz, 1H, H-5); ^13^C NMR: δ 30.1, 118.0 (d, ^2^*J*_CF_ = 20.5 Hz), 121.1, 122.3 (d, ^2^*J*_CF_ = 13.2 Hz), 125.0 (d, ^4^*J*_CF_ = 4.0 Hz), 131.05, 131.09, 136.9, 137.4, 138.3 (d, ^3^*J*_CF_ = 8.8 Hz), 138.7, 144.4, 159.7 (d, ^1^*J*_CF_ = 262.3 Hz), 195.1; ^19^F NMR: δ −105.3.

#### 3.2.14. 2-Acetyl-4-bromo-6-nitrophenyl 3-fluorobenzenesulfonate (**2n**)

Light brown solid (1.47 g, 91%), mp. 110–112 °C; ν_max_ (ATR) 579, 677, 777, 790, 853, 1086, 1169, 1175, 1344, 1537, 1705, 3069 cm^−1^; ^1^H NMR: δ 2.60 (s, 3H, -CH_3_), 7.40–7.50 (m, 1H, H-4′), 7.55–7.57 (m, 1H, H-5′), 7.60 (td, *J* = 5.0 and 8.1 Hz, 1H, H-2′), 7.64–7.66 (m, 1H, H-6′), 7.99 (d, *J* = 2.5 Hz, 1H, H-3), 8.13 (d, *J* = 2.5 Hz, 1H, H-6); ^13^C NMR: δ 30.0, 116.1 (d, ^2^*J*_CF_ = 25.4 Hz), 121.2, 123.1 (d, ^2^*J*_CF_ = 21.2 Hz), 124.7 (d, ^4^*J*_CF_ = 3.5 Hz), 131.1, 131.7 (d, ^3^*J*_CF_ = 7.9 Hz), 135.2 (d, ^3^*J*_CF_ = 7.4 Hz), 136.96, 137.01, 138.6, 144.6, 162.4 (d, ^1^*J*_CF_ = 254.1 Hz), 194.8; ^19^F NMR: δ −107.4.

#### 3.2.15. 2-Acetyl-4-bromo-6-nitrophenyl 4-fluorobenzenesulfonate (**2o**)

Yellow solid (1.51 g, 94%), mp. 127–129 °C; ν_max_ (ATR) 536, 538, 684, 739, 741, 840, 854, 1087, 1155, 1174, 1201, 1336, 1386, 1485, 1531, 1539, 1589, 1705, 3074 cm^−1^; ^1^H NMR: δ 2.63 (s, 3H, -CH_3_), 7.29 (dd, *J* = 4.0 and 9.0 Hz, 2H, H-3′,5′), 7.89 (dd, *J* = 4.0 and 9.0 Hz, 2H, H-2′,6′), 8.02 (d, *J* = 2.5 Hz, 1H, H-3), 8.14 (d, *J* = 2.5 Hz, 1H, H-6); ^13^C NMR: δ 30.0, 117.3 (d, ^2^*J*_CF_ = 23.0 Hz), 121.1, 129.3 (d, ^4^*J*_CF_ = 3.2 Hz), 131.1, 131.9 (d, ^3^*J*_CF_ = 10.1 Hz), 137.0, 137.2, 138.7, 144.7, 167.0 (d, ^1^*J*_CF_ = 260.6 Hz), 194.9; ^19^F NMR: δ −98.8.

#### 3.2.16. 2-Acetyl-4-bromo-6-nitrophenyl 4-(trifluoromethyl)benzenesulfonate (**2p**)

Green solid (1.50 g, 83%), mp. 136–138 °C; ν_max_ (ATR) 528, 569, 592, 677, 714, 777, 847, 1060, 1123, 1159, 1207, 1321, 1392, 1530, 1701, 3051 cm^−1^; ^1^H NMR: δ 2.60 (s, 3H, -CH_3_), 7.86 (d, *J* = 7.8 Hz, 2H, H-3′,5′), 7.99–8.02 (m, 3H, H-2′,6′ and H-3), 8.11–8.13 (m, 1H, H-5); ^13^C NMR: δ 29.7, 121.1, 122.5 (q, ^1^*J*_CF_ = 273.5 Hz), 126.6 (q, ^3^*J*_CF_ = 3.7 Hz), 129.1, 130.9, 136.4, 136.7, 136.9 (q, ^2^*J*_CF_ = 33.5 Hz), 138.4, 138.7, 144.3, 194.5; ^19^F NMR: δ −63.6.

#### 3.2.17. 2-Acetyl-4-bromo-6-nitrophenyl 4-(trifluoromethoxy)benzenesulfonate (**2q**)

Yellow solid (1.62 g, 87%), mp. 119–121 °C; ν_max_ (ATR) 563, 662, 719, 775, 851, 1084, 1157, 1179, 1201, 1342, 1537, 1701, 3022 cm^−1^; ^1^H NMR: δ 2.61 (s, 3H, -CH_3_), 7.41 (d, *J* = 9.0 Hz, 2H, H-3′,5′), 7.90–7.94 (m, 2H, H-2′,6′), 8.00 (d, *J* = 2.5 Hz, 1H, H-3), 8.12 (d, *J* = 2.5 Hz, 1H, H-5); ^13^C NMR: δ 30.0, 120.1 (q, ^1^*J*_CF_ = 260.7 Hz), 121.2 (q, ^4^*J*_CF_ = 1.2 Hz), 131.1, 131.2, 131.4, 136.9, 137.0, 138.7, 144.7, 154.4 (q, ^3^*J*_CF_ = 1.8 Hz), 194.8; ^19^F NMR: δ −57.7.

#### 3.2.18. 2-Acetyl-4-bromo-6-nitrophenyl 4-methoxybenzenesulfonate (**2r**)

Grey solid (1.38 g, 83%), mp. 96–98 °C; ν_max_ (ATR) 530, 542, 584, 718, 764, 849, 1015, 1084, 1163, 1195, 1350, 1496, 1533, 1539, 1693, 2960, 3076 cm^−1^; ^1^H NMR: δ 2.60 (s, 3H, -CH_3_), 3.91 (s, 3H, -OCH_3_), 7.01 (d, *J* = 7.8 Hz, 2H, H-3′,5′), 7.72 (d, *J* = 7.8 Hz, 2H, H-2′,6′), 7.98 (s, 1H, H-3), 8.09 (s, 1H, H-5); ^13^C NMR: δ 30.0, 55.9, 115.0, 120.7, 124.0, 131.0, 131.2, 137.0, 137.8, 138.8, 144.9, 165.4, 195.0.

#### 3.2.19. 2-Acetyl-4-methyl-6-nitrophenyl 2-fluorobenzenesulfonate (**2s**)

Yellow solid (1.78 g, 98%), mp. 110–112 °C; ν_max_ (ATR) 513, 569, 715, 767, 829, 1074, 1175, 1271, 1354, 1474, 1533, 1695, 3038 cm^−1^; ^1^H NMR: δ 2.45 (s, 3H, -CH_3_), 2.61 (s, 3H, -CH_3_), 7.24–7.33 (m, 2H, H-3′, H-4′), 7.64 (d, *J* = 2.3 Hz, 1H, H-3), 7.71–7.78 (m, 2H, H-5′, H-6′), 7.81 (d, *J* = 2.3 Hz, 1H, H-5); ^13^C NMR: δ 20.8, 30.2, 117.9 (d, ^2^*J*_CF_ = 20.6 Hz), 122.6 (d, ^2^*J*_CF_ = 13.2 Hz), 124.9 (d, ^4^*J*_CF_ = 3.9 Hz), 128.6, 131.1, 134.5, 136.0, 137.1, 138.0 (d, ^3^*J*_CF_ = 8.8 Hz), 139.1, 143.7, 159.7 (d, ^1^*J*_CF_ = 262.0 Hz), 196.9; ^19^F NMR: δ −105.4.

#### 3.2.20. 2-Acetyl-4-methyl-6-nitrophenyl 3-fluorobenzenesulfonate (**2t**)

Yellow solid (1.69 g, 93%), mp. 114–116 °C; ν_max_ (ATR) 503, 576, 673, 712, 779, 837, 844, 1086, 1171, 1180, 1354, 1346, 1541, 1705, 3076 cm^−1^; ^1^H NMR: δ 2.47 (s, 3H, -CH_3_), 2.58 (s, 3H, -CH_3_), 7.43–7.47 (m, 1H, H-4′), 7.52–7.54 (m, 1H, H-5′), 7.57 (td, *J* = 5.1 and 8.1 Hz, 1H, H-2′), 7.63–7.65 (m, 1H, H-6′), 7.67 (d, *J* = 2.3 Hz, 1H, H-3), 7.81 (d, *J* = 2.3 Hz, 1H, H-5); ^13^C NMR: δ 20.9, 30.1, 116.1 (d, ^2^*J*_CF_ = 25.3 Hz), 122.7 (d, ^2^*J*_CF_ = 21.1 Hz), 124.6 (d, ^4^*J*_CF_ = 3.5 Hz), 128.7, 131.6 (d, ^3^*J*_CF_ = 7.8 Hz), 134.6, 135.5 (d, ^3^*J*_CF_ = 7.4 Hz), 135.7, 137.1, 139.2, 143.9, 162.3 (d, ^1^*J*_CF_ = 253.6 Hz), 196.6; ^19^F NMR: δ −107.9.

#### 3.2.21. 2-Acetyl-4-methyl-6-nitrophenyl 4-fluorobenzenesulfonate (**2u**)

Yellow solid (1.70 g, 94%), mp. 130–132 °C; ν_max_ (ATR) 534, 540, 688, 692, 694, 700, 777, 839, 1083, 1161, 1165, 1184, 1352, 1354, 1490, 1537, 1548, 1697, 3062 cm^−1^; ^1^H NMR: δ 2.46 (s, 3H, -CH_3_), 2.59 (s, 3H, -CH_3_), 7.24 (dd, *J* = 4.0 and 9.0 Hz, 2H, H-3,5), 7.67 (d, *J* = 2.3 Hz, 1H, H-3), 7.79 (d, *J* = 2.3 Hz, 1H, H-5), 7.85 (dd, *J* = 4.0 and 9.0 Hz, 2H, H-2′,6′); ^13^C NMR: δ 20.8, 30.1, 117.1 (d, ^2^*J*_CF_ = 23.0 Hz), 128.6, 129.6 (d, ^4^*J*_CF_ = 3.2 Hz), 131.8 (d, ^3^*J*_CF_ = 10.1 Hz), 134.6, 135.8, 137.2, 139.0, 144.0, 166.8 (d, ^1^*J*_CF_ = 259.8 Hz), 196.6; ^19^F NMR: δ −99.7.

#### 3.2.22. 2-Acetyl-4-methyl-6-nitrophenyl 4-(trifluoromethyl)benzenesulfonate (**2v**)

Orange solid (1.92 g, 93%), mp. 110–112 °C; ν_max_ (ATR) 519, 594, 694, 719, 839, 1063, 1124, 1169, 1323, 1390, 1526, 1695, 3046 cm^−1^; ^1^H NMR: δ 2.48 (s, 3H, -CH_3_), 2.59 (s, 3H, -CH_3_), 7.68 (d, *J* = 1.4 Hz, 1H, H-3), 7.81 (d, *J* = 1.4 Hz, 1H, H-5), 7.85 (d, *J* = 8.4 Hz, 2H, H-3′,5′), 8.00 (d, 2H, H-2′,6′); ^13^C NMR: δ 20.6, 29.8, 122.6 (q, ^1^*J*_CF_ = 273.4 Hz), 126.5 (q, ^3^*J*_CF_ = 3.6 Hz), 128.4, 129.1, 134.3, 135.1, 136.5 (q, ^2^*J*_CF_ = 33.5 Hz), 136.9, 137.2, 139.0, 143.7, 196.2; ^19^F NMR: δ −63.6.

#### 3.2.23. 2-Acetyl-4-methyl-6-nitrophenyl 4-(trifluoromethoxy)benzenesulfonate (**2w**)

Yellow solid (1.87 g, 87%), mp. 102–104 °C; ν_max_ (ATR) 521, 567, 677, 714, 775, 1086, 1161, 1192, 1385, 1533, 1699, 3045 cm^−1^; ^1^H NMR: δ 2.47 (s, 3H, -CH_3_), 2.59 (s, 3H, -CH_3_), 7.39 (d, *J* = 9.0 Hz, 2H, H-3′,5′), 7.68 (d, *J* = 2.3 Hz, 1H, H-3), 7.80 (d, *J* = 2.3 Hz, 1H, H-5), 7.88–7.92 (m, 2H, H-2′,6′); ^13^C NMR: δ 20.8, 30.1, 120.1 (q, ^1^*J*_CF_ = 260.5 Hz), 121.1 (q, ^4^*J*_CF_ = 1.2 Hz), 128.6, 131.2, 131.7, 134.6, 135.6, 137.1, 139.1, 144.0, 154.2 (q, ^3^*J*_CF_ = 1.8 Hz), 196.5; ^19^F NMR: δ −57.7.

#### 3.2.24. 2-Acetyl-4-methyl-6-nitrophenyl 4-methoxybenzenesulfonate (**2x**)

Grey solid (1.75 g, 93%), mp. 128–130 °C; ν_max_ (ATR) 522, 571, 694, 777, 827, 1016, 1086, 1163, 1269, 1346, 1496, 1537, 1699, 2908, 3041 cm^−1^; ^1^H NMR: δ 2.45 (s, 3H, -CH_3_), 2.58 (s, 3H, -CH_3_), 3.90 (s, 3H, -OCH_3_), 6.98 (d, *J* = 9.0 Hz, 2H, H-3′,5′), 7.66 (d, *J* = 1.4 Hz, 1H, H-3), 7.70 (d, *J* = 9.0 Hz, 2H, H-2′,6′), 7.78 (d, *J* = 1.4 Hz, 1H, H-5); ^13^C NMR: δ 20.8, 30.1, 55.9, 114.9, 124.4, 128.6, 131.1, 134.5, 136.4, 137.2, 138.6, 144.2, 165.2, 196.7.

### 3.3. Single Crystal X-Ray Diffraction Analysis of ***2o***

Intensity data was determined on a Bruker D8 Venture Microfocus with Photon III CCD area detector diffractometer with graphite monochromated MoKα_1_ (l = 0.71073 Å) radiation at 173 K using an Oxford Cryostream 600 cooler. Data reduction, empirical absorption corrections and space group assignments were carried out using the program SAINT+, version 6.02 (version 2; Bruker AXS Inc., Madison, WI, USA, 2016). The empirical absorption corrections were made using SADABS (Bruker AXS Inc., Madison, WI, USA, 2016). The structure of **2o** was solved in the WinGX Suite of programs [65], using intrinsic phasing through SHELXT [66]. The data was refined using full-matrix least-squares/difference Fourier techniques on F2 using SHELXL-2018/3 [67]. All carbon (C)-bound hydrogen atoms were placed at idealized positions. The atoms were refined as riding atoms with isotropic parameters 1.2 or 1.5 times those of their parent atoms. Diagrams and publication material were generated using ORTEP-3 [66] and PLATON [68]. Experimental details of the X-ray analyses for **2o** are provided in Table S1 of the Supplementary Materials.

### 3.4. Biology

#### 3.4.1. In Vitro α-Glucosidase Inhibitory Assay of **2a**–**x**

The tests were performed in triplicates using a 96-well plate following a method described in our previous study [31]. The stock solutions (200 µM) of the test compounds and acarbose were prepared in DMSO and then diluted with a 100 mM phosphate buffer to obtain the concentrations of 1, 5, 10, 25 and 50 µM. A mixture of α-glucosidase (17 µL) from *Saccharomyces cerevisiae* (0.48 u/mL α-glucosidase), phosphate buffer (100 mM, pH 6.8; 50 µL) and test sample in DMSO was incubated at 37 °C for 10 min. After 10 min., a solution of 2 mM *p*-nitrophenyl-α-D-glucopyranoside (PNP-G, 17 µL) was added to each well to initiate the reaction. The plate was incubated for 30 min at 37 °C. Five absorbance readings were recorded for each triplicate run at a wavelength of 405 nm using Varioskan flash microplate spectrophotometer (Thermo Scientific, Waltham, MA, USA). The average values obtained from the readings were used to determine the IC_50_ and standard deviation values. The values were calculated by nonlinear regression analysis and expressed as the mean SD of three distinct experiments using Graph Pad Prism software.

#### 3.4.2. In Vitro α-Amylase Inhibitory Assay on **2a**, **2e**, **2f**, **2g**, **2j**, **2k**, **2v** and **2x**

The α-amylase assay was performed in triplicate using a 96-well plate using the α-Amylase Inhibitor Screening Kit (Catalog No. K482; Abcam, Cambridge, UK). The procedure was carried out according to the manufacturer’s protocol as outlined in the Kit by referring to the instructions reported in our previous study [31]. The stock solutions (100 μM) of the test compounds and acarbose were prepared in DMSO and then diluted with α-amylase assay buffer to obtain final concentrations of 1, 5, 10, 25 and 50 μM. A solution of α-amylase enzyme (50 μL) prepared by adding 490 μL of assay buffer to 10 μL of α-amylase enzyme was added to each of the wells containing the reaction mixture to initiate the reaction. The plate was incubated for 10 min. at room temperature in the dark. Five different absorbance readings were recorded for each triplicate run at a wavelength of 405 nm using Varioskan flash microplate spectrophotometer (Thermo Scientific, Waltham, MA, USA). The IC_50_ and SD values were calculated using the Graph Pad Prism software.

### 3.5. In Vitro PTP1B Inhibitory Assay *on* ***2a***, ***2e***, ***2f***, ***2g***, ***2j***, ***2k***, ***2v*** and ***2x***

The PTP1B assay was performed in duplicate using a 96-well plate. The procedure was carried out according to the manufacturer’s protocol as outlined in the Fluorogenic PTP1B (Catalytic Domain) Assay (Catalogue No. #79764; Abcam, Cambridge, UK). The stock solutions (200 μM) of the test compounds and the reference standard, sodium orthovanadate, were prepared in DMSO, and then diluted with PTP1B assay buffer to obtain a concentration of 100 μM. From this dilution appropriate volumes of each compound and the standard were placed in the 96-well plates to obtain the final concentrations of 1, 5, 10, 25 and 50 µM for the assay. The PTP1B enzyme (2 pg/μL) was added to the wells and the mixtures were incubated at room temperature. After 30 min of incubation, the fluorescence intensity was measured at 360 nm excitation and 460 nm emission wavelengths using an Elisa plate reader. The blank value was subtracted from all other values. The IC_50_ and SD values were calculated using GraphPad prism version 8.0 and Excel (Version 2409), respectively.

### 3.6. Inhibition of VGEFR-2 Tyrosine Phosphorylation by ***2a***, ***2e***, ***2f***, ***2g***, ***2j***, ***2k***, ***2v*** and ***2x***

The effect of these selected compounds on VEGFR-2 activity was carried out employing the Human VEGFR-2 (KDR) kinase assay kit (BPS Bioscience Corporation catalogue #40325; San Diego, CA, USA). The procedure was carried out according to the manufacturer’s protocol by referring to the instructions reported in our previous study [69]. The test compounds as well as the reference standard nintedanib, at concentrations 1, 5, 10, 25 and 50 μM were added to the micro-ELISA plate wells. The VEGFR-2 enzyme (1 ng/μL) was added to the wells and the mixtures were incubated at 30 °C. After 45 min of incubation, kinase detection reagent (50 μL, Kinase-Glo MAX, Promega) was added to each well. The mixtures were incubated for 15 min at room temperature in the dark. A luminescence reading was detected and quantified by a microplate reader (Varioskan Flash, ThermoFisher Scientific, Vantaa, Finland). The IC_50_ values were determined by the nonlinear regression analysis using GraphPad Prism software version 8.0 (GraphPad Software, Inc., San Diego, CA, USA).

### 3.7. Cytotoxicity Study on ***2a***, ***2e***, ***2f***, ***2g***, ***2j***, ***2k***, ***2v*** and ***2x***

The cytotoxic activity of these compounds was evaluated in vitro (triplicate run) on the MCF-7, A549 and Vero cell lines using the CellTiter-Blue Cell viability assay (Promega, Madison, WI, USA) assay referring to the reported instructions as described in our previous study [70]. The cells were maintained in culture flasks in the presence of Dulbecco’s Modified Eagle’s Medium (DMEM; Gibco, Carlsbad, CA, USA) supplemented with 10% fetal bovine serum (FBS) and 1% penicillin/streptomycin and then incubated at 37 °C in 5% CO_2_. The cells were detached using 2% trypsin upon reaching 85% confluency and counted with a hand-held automated cell counter (Scapter 3.0™, Merck, Burlington, MA, USA). The cells were then seeded at 1 × 105 cells/well and the well-plates were incubated overnight to allow cell attachment. After 24 h, treatments were administered with different concentrations (1, 5, 10, 25, 50 and 100 µM) of the test compounds and the reference standards (doxorubicin and nintedanib). The mixtures were incubated for another 24 h and then 20 µL of the CellTiter-Blue solution was added to each well. Fluorescence was measured using an Elisa microplate reader (ThermoFisher Scientific, Vario SkanFlash, Vantaa, Finland) at 560 nm excitation/590 nm emission. The cell viability (triplicate results) was calculated using a GraphPad Prism software (Version 8, GraphPad Software Inc., San Diego, CA, USA) to fit the nonlinear regression dose-response curves to obtain the IC_50_ and standard deviation values.

### 3.8. Molecular Docking Studies of ***2a***, ***2e***, ***2f***, ***2g***, ***2j***, ***2k***, ***2v*** and ***2x*** into α-Glucosidase and into α-Amylase

Discovery Studio software version 22.1.100.22290 (Accelrys, San Diego, CA, USA) was used to construct and prepare ligands and proteins were also prepared for docking. The PDB structures selected were 5NN8 and 3BAJ for α-glucosidase and α-amylase, respectively. The binding site coordinates (x, y, z) for α-glucosidase were −16.5323, −38.2485, 96.4584 with a radius of 12 and for α-amylase were 10.5622, 16.2775, 37.4422 with a radius of 12.1. For both proteins, acarbose was also prepared and docked into the binding site but for α-amylase the ligand that was co-crystalized with the protein was also re-docked to confirm the binding site selection. The PDB structures for the PTP1B catalytic (code: 2QBP) and allosteric site (code: 1T49) as well as for VEGFR-2 (PDB 4ASD) were used. The binding site coordinates (x, y, z) for 2QBP, 1T49 and 4ASD: 47.0042, 12.7734, −3.29397; 53.1785, 29.1109, 22.6947 and −26.0997, 0.65999, −11.7026 with radii of 17.4, 10.7 and 12.2, respectively. C-Docker was used to dock ligands to the binding site followed by a binding energy calculation for the top 5 to 7 poses as ranked by cdocker and cdocker interaction energies. The binding energy calculation parameters that were altered from the default values were flexing binding site protein residues and using the Generalized Born with Molecular Volume (GBMV) solvent model. Poses with the best binding energy and preferably with no unfavourable interactions were selected.

### 3.9. Prediction of Pharmacokinetic Properties for ***2a***, ***2e***, ***2f***, ***2g***, ***2j***, ***2k***, ***2v*** and ***2x***

The compounds were prepared using Discovery Studio version v22.1.000.22290 and were saved in SMILES format, and then entered into the online tool Swiss ADME (http://www.swissadme.ch/index.php accessed on 14 October 2024) [66] and the data was collected on the 1 October 2024.

## 4. Conclusions

The benzenesulfonic ester derivatives exhibited strong inhibitory activity against α-glucosidase compared to the parent 5-substituted 2-hydroxy-3-nitroacetophenones. Replacement of the *ortho*-hydroxyl group on scaffold of the 5-fluoro-, 5-chloro and 5-methyl substituted 3-nitroacetophenones with a 2-fluorophenylsulfonyl moiety favoured activity against α-glucosidase. Similarly, a fluorine-containing group (-CF_3_ or -OCF_3_) at the *para* position of the scaffold of compounds **2** favoured inhibition against α-glucosidase activity for all the sub-series. The 4-(trifluoromethoxyphenyl)sulfonyl substituted derivatives exhibited strong anti–α-glucosidase activity compared to the analogous 4-(methoxyphenylsulfonyl) derivatives **2f** and **2x** thus, confirming the influence of the fluorinated benzenesulfonyl group on biological activity consistent with the design strategy. The fluorinated benzenesulfonic esters **2a**, **2g**, **2j**, **2k** and **2v** and the analogue **2x** with dual inhibition of both enzymes, but moderate inhibitory effect against α-amylase will probably inhibit T2DM with reduced or no gastrointestinal side effects. Compounds **2a** and **2v** are potential MTDL against T2DM capable of inhibiting both carbohydrate hydrolyzing enzymes and PTP1B. The 2-(4-(trifluoromethylphenyl)sulfonyl)-5-methyl-3-nitroacetophenone derivative **2v** also exhibited moderate inhibitory effect against VEGFR-2 activity. Hydrophilic (hydrogen and/or halogen bonds), hydrophobic (eg, π-π stacking, π-alkyl & alkyl) and electrostatic (π-anion and salt bridges) interactions helped to embed these ligands in the active sites of both α-glucosidase and α-amylase to inhibit enzyme activity. The test compounds exhibited moderate and significant cytotoxicity against the MCF-7 cell line and the A549 ell line, respectively, with significantly reduced toxicity towards the Vero cell line. This makes the test compounds suitable MTDLs to mitigate PPHG and inhibit cancer with minimal or no toxicity to the normal cells. However, only bioactivity studies using in vivo diabetic models will help well to define the underlying potential of these compounds.

## Figures and Tables

**Table 1:** Substitution pattern and designation of Ar and X for compounds **2a**–**x**.

Ar	X (Compound Number)			
-C_6_H_4_(2-F)	F (**2a**)	Cl (**2g**)	Br (**2m**)	-CH_3_ (**2s**)
-C_6_H_4_(3-F)	F (**2b**)	Cl (**2h**)	Br (**2n**)	-CH_3_ (**2t**)
-C_6_H_4_(4-F)	F (**2c**)	Cl (**2i**)	Br (**2o**)	-CH_3_ (**2u**)
-C_6_H_4_(4-CF_3_)	F (**2d**)	Cl (**2j**)	Br (**2p**)	-CH_3_ (**2v**)
-C_6_H_4_(4-OCF_3_)	F (**2e**)	Cl (**2k**)	Br (**2q**)	-CH_3_ (**2w**)
-C_6_H_4_(4-OCH_3_)	F (**2f**)	Cl (**2l**)	Br (**2r**)	-CH_3_ (**2x**)

**Table 2:** The IC_50_ values of **1** and **2** against α-glucosidase, α-amylase, PTP1B and VEGFR-2 activities.

Compound	IC_50_ (µM ± SD)			
	α-Glucosidase	α-Amylase	PTP1B	VEGFR-2
**1a**	16.9 ± 0.064	-	-	-
**1b**	27.2 ± 0.195	-	-	-
**1c**	11.9 ± 0.010	-	-	-
**1d**	8.2 ± 0.015	-	-	-
**2a**	5.6 ± 0.038	7.9 ± 0.054	6.3 ± 0.029	28.7 ± 0.27
**2b**	8.6 ± 0.025	-	-	-
**2c**	27.4 ± 0.044	-	-	-
**2d**	6.4 ± 0.012	18.1 ± 0.185	-	-
**2e**	3.1 ± 0.043	15.2 ± 0.019	23.1 ± 0.006	28.5 ± 0.23
**2f**	5.3 ± 0.159	3.1 ± 0.110	5.9 ± 0.018	28.3 ± 031
**2g**	4.2 ± 0.054	7.0 ± 0.040	16.9 ± 0.016	34.4 ± 0.26
**2h**	12.4 ± 0.041	-	-	-
**2i**	10.6 ± 0.039	-	-	-
**2j**	5.4 ± 0.030	7.3 ± 0.008	19.6 ± 0.042	20.8 ± 0.35
**2k**	3.9 ± 0.031	9.8 ± 0.021	20.1 ± 0.065	26.4 ± 0.20
**2l**	8.5 ± 0.043	-	-	-
**2m**	7.9 ± 0.044	-	-	-
**2n**	18.2 ± 0.041	-	-	-
**2o**	20.5 ± 0.058	-	-	-
**2p**	8.0 ± 0.060	-	-	-
**2q**	8.7 ± 0.075	-	-	-
**2r**	13.2 ± 0.043	-	-	-
**2s**	18.1 ± 0.048	-	-	-
**2t**	13.1 ± 0.045	-	-	-
**2u**	11.2 ± 0.045	-	-	-
**2v**	3.0 ± 0.014	6.0 ± 0.020	7.6 ± 0.070	16.3 ± 0.29
**2w**	6.0 ± 0.034	16.7 ± 0.035	-	-
**2x**	3.6 ± 0.029	11.9 ± 0.022	21.5 ± 0.066	24.2 ± 0.22
Acarbose	6.4 ± 0.134	5.4 ± 0.131	-	-
Na_3_VO_4_	-	-	6.3 ± 0.050	-
Nintedanib	-	-	-	1.4 ± 0.42

**Table 3:** Cytotoxicity of **2a**, **2e**, **2f**, **2g**, **2j**, **2k**, **2v** and **2x** against the MCF-7, A549 and Vero cell lines.

Compounds	IC_50_ (µM ± SD)		
	MCF-7	A549	Vero
**2a**	4.96 ± 0.05	7.75 ± 0.11	39.21 ± 0.13
**2e**	8.18 ± 0.03	7.42 ± 0.09	20.78 ± 0.14
**2f**	4.38 ± 0.07	1.91 ± 0.01	24.03 ± 1.01
**2g**	9.92 ± 0.09	6.15 ± 0.06	33.93 ± 0.15
**2j**	2.58 ± 0.02	2.52 ± 0.02	38.86 ± 0.16
**2k**	34.53 ± 0.12	1.89 ± 0.05	33.39 ± 0.13
**2v**	8.58 ± 0.07	5.50 ± 0.01	48.02 ± 0.18
**2x**	11.01 ± 0.07	1.01 ± 0.01	56.77 ± 0.18
Doxorubicin	0.59 ± 0.03	0.71 ± 0.09	0.94 ± 0.04
Nintedanib	0.40 ± 0.03	0.78 ± 0.04	0.24 ± 0.02

**Table 4:** Pharmacokinetics properties predictions of compounds **2a**, **2e**, **2f**, **2g**, **2j**, **2k**, **2v** and **2x**.

Property	2a	2e	2f	2g	2j	2k	2v	2x
XLogP3	2.73	3.82	2.61	3.26	4.05	4.34	3.78	2.87
mLogP	2.44	2.14	1.78	2.56	3.05	2.26	2.78	1.63
TPSA (Å)	114.64	123.87	123.87	114.64	114.64	123.87	114.64	123.87
Solubility (Log S)	−3.82	−4.73	−3.73	−4.25	−4.95	−5.16	−4.65	−3.87
GI Absorption	High	Low	High	High	Low	Low	Low	High
Cytochrome P450 enzyme inhibitor *	2C19 3A4	2C19, 2C9, 2D6, 3A4	2C19, 2C9, 2D6, 3A4	2C19, 2C9, 3A4	2C19, 2C9, 3A4	2C19, 2C9, 3A4	1A2, 2C19, 2C9,3A4	1A2, 2C19,2C9, 2D6, 3A4
Saturation (fraction Csp^3^)	0.07	0.13	0.13	0.07	0.13	0.13	0.19	0.19
MW(g/mol)	357.29	423.29	369.32	373.74	423.75	439.75	403.33	365.36
H-bond acceptor	8	11	8	7	9	10	9	7
H-bond donor	0	0	0	0	0	0	0	0
Rotatable bonds	5	7	6	5	6	7	6	6
Lipinski’s violation	none	none	none	none	none	none	none	none

* The Cytochrome P450 enzymes include CYP1A2, CYP2C19, CYP2C9, CYP2D6, CYP3A4.

## Data Availability

The original contributions presented in the study are included in the article/Supplementary Materials, further inquiries can be directed to the corresponding author.

## Supplementary Materials

The following supporting information can be downloaded at: https://www.mdpi.com/article/10.3390/ijms252211862/s1.
