# Supplementary material for: Enzyme (α-Glucosidase, α-Amylase, PTP1B & VEGFR-2) Inhibition and Cytotoxicity of Fluorinated Benzenesulfonic Ester Derivatives of the 5-Substituted 2-Hydroxy-3-nitroacetophenones
Source: Int J Mol Sci. 2024 Nov 5;25(22):11862. doi: 10.3390/ijms252211862 (PMC11594133; doi:10.3390/ijms252211862)
Supplement: Supplementary file 1 [file ijms-25-11862-s001.zip › ijms-3288199-supplementary.pdf]

Supplementary Materials

**Figures S1:**	^{1}H-, ^{13}C- and ^{19}F NMR of **2a–x**
**Table S1:**	The crystal data and structure refinement of **2o**
**Figure S2:**	%Inhibition curves of compounds **2a–x** against α-glucosidase
**Figure S3:**	%Inhibition curves of **2a**, **2d**, **2e**, **2f**, **2g**, **2j**, **2k**, **2v**, **2w** and **2x** against α-amylase
**Figure S4:**	%Inhibition curves of **2a**, **2e**, **2f**, **2g**, **2j**, **2k**, **2v** and **2x** against PTP1B
**Figure S5:**	%Inhibition curves of **2a**, **2e**, **2f**, **2g**, **2j**, **2k**, **2v** and **2x** against VEGFR-2
**Figure S6:**	%Inhibition curves of **2a**, **2e**, **2f**, **2g**, **2j**, **2k**, **2v** and **2x** against the MCF-7 cell line
**Figure S7:**	%Inhibition curves of **2a**, **2e**, **2f**, **2g**, **2j**, **2k**, **2v** and **2x** against the A543 cell line
**Figure S8:**	%Inhibition curves of **2a**, **2e**, **2f**, **2g**, **2j**, **2k**, **2v** and **2x** against the Vero cell line
**Figure S9:**	Docking of **2a**, **2e**, **2f**, **2g**, **2j**, **2k**, **2v** and **2x** into α-glucosidase
**Figure S10:**	Docking of **2a**, **2e**, **2f**, **2g**, **2j**, **2k**, **2v** and **2x** into α-amylase
**Figure S11:**	Docking of **2a**, **2e**, **2f**, **2g**, **2j**, **2k**, **2v** and **2x** into PTP1B active site
**Figure S12:**	Docking of **2a**, **2e**, **2f**, **2g**, **2j**, **2k**, **2v** and **2x** into PTP1B allosteric site
**Figure S13:**	Docking of **2a**, **2e**, **2f**, **2g**, **2j**, **2k**, **2v** and **2x** into VEGFR-2 active site

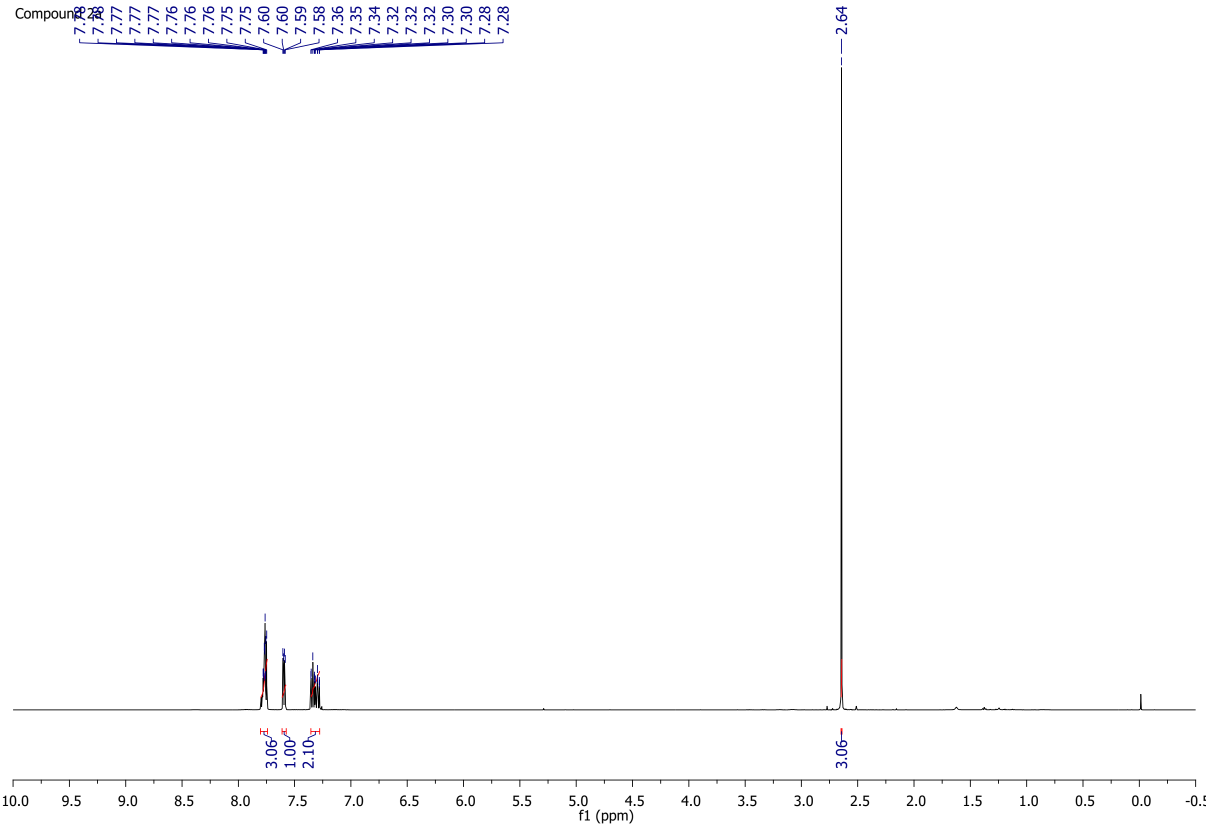
7.77
7.77
7.77
7.76
7.76
7.76
7.75
7.75
7.60
7.60
7.59
7.58
7.36
7.35
7.34
7.32
7.32
7.32
7.30
7.30
7.28
7.28
2.64
3.06
1.00
2.10
3.06
10.0 9.5 9.0 8.5 8.0 7.5 7.0 6.5 6.0 5.5 5.0 4.5 4.0 3.5 3.0 2.5 2.0 1.5 1.0 0.5 0.0
f1 (ppm)

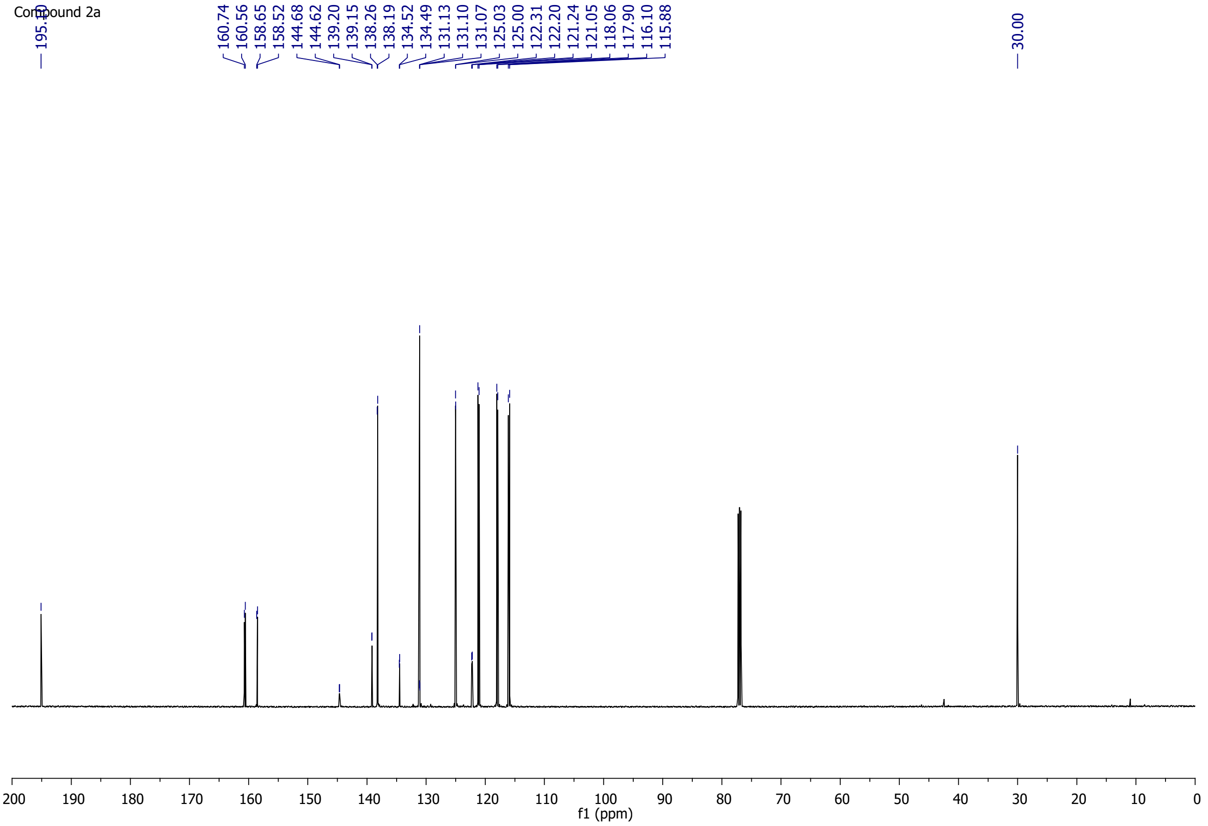
Compound 2a
195.[illegible]
160.74
160.56
158.65
158.52
144.68
144.62
139.20
139.15
138.26
138.19
134.52
134.49
131.13
131.10
131.07
125.03
125.00
122.31
122.20
121.24
121.05
118.06
117.90
116.10
115.88
30.00
200 190 180 170 160 150 140 130 120 110 100 90 80 70 60 50 40 30 20 10 0
f1 (ppm)

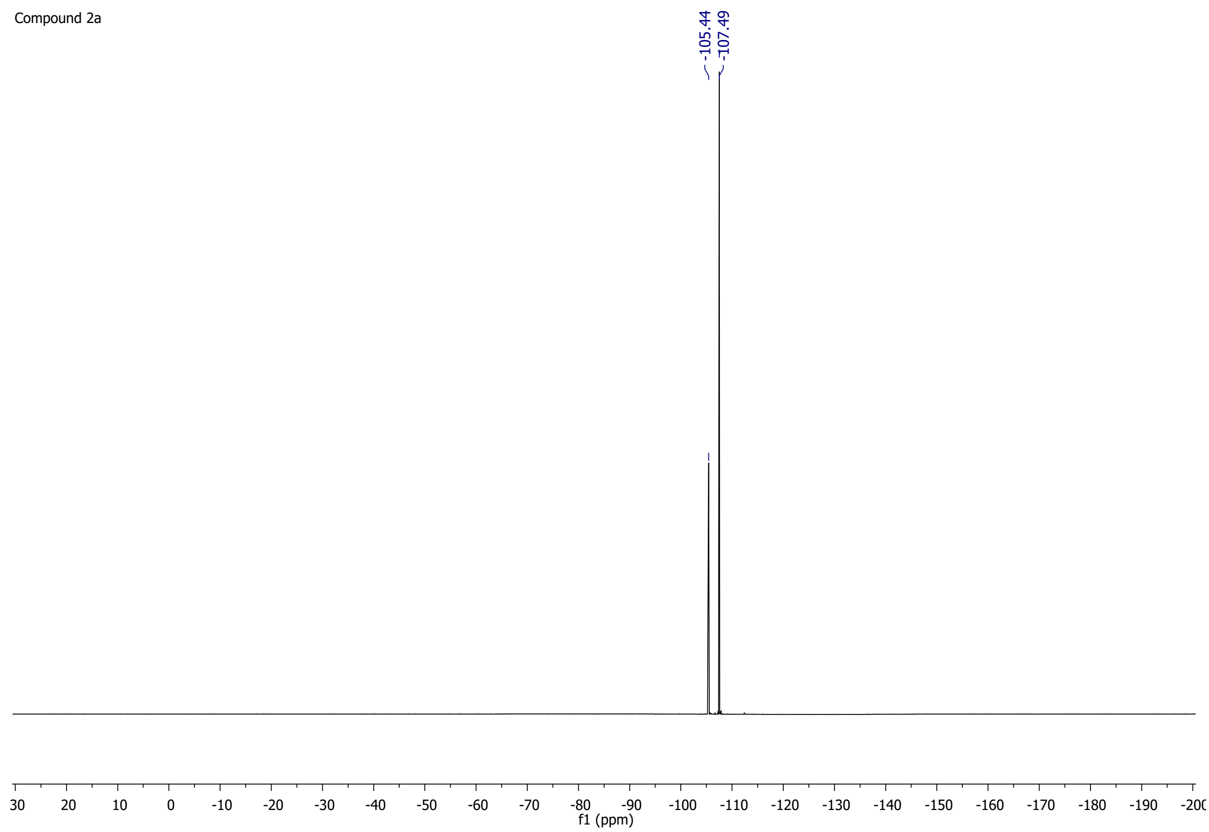

Figure S1.1: ^{1}H-, ^{13}C- and ^{19}F NMR of **2a** in $CDCl_3$ at 500, 125 and 470 MHz, respectively.

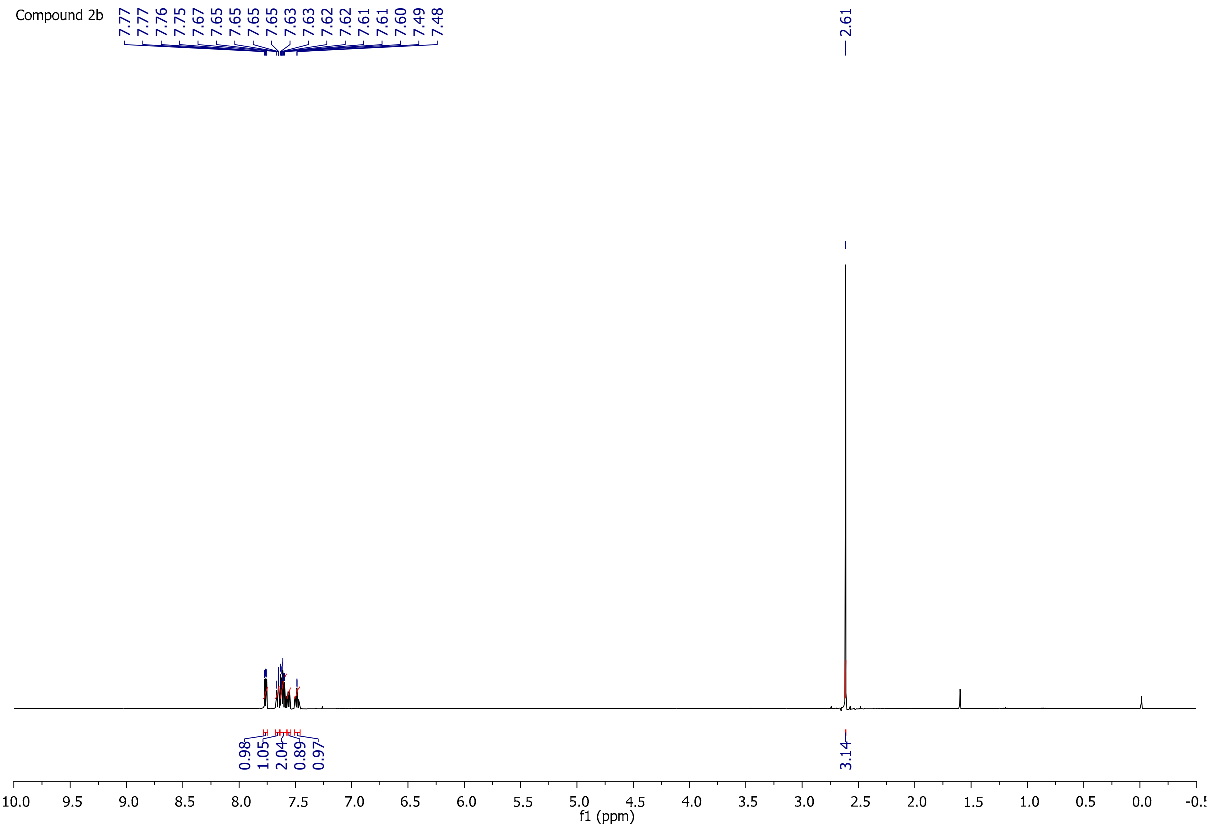

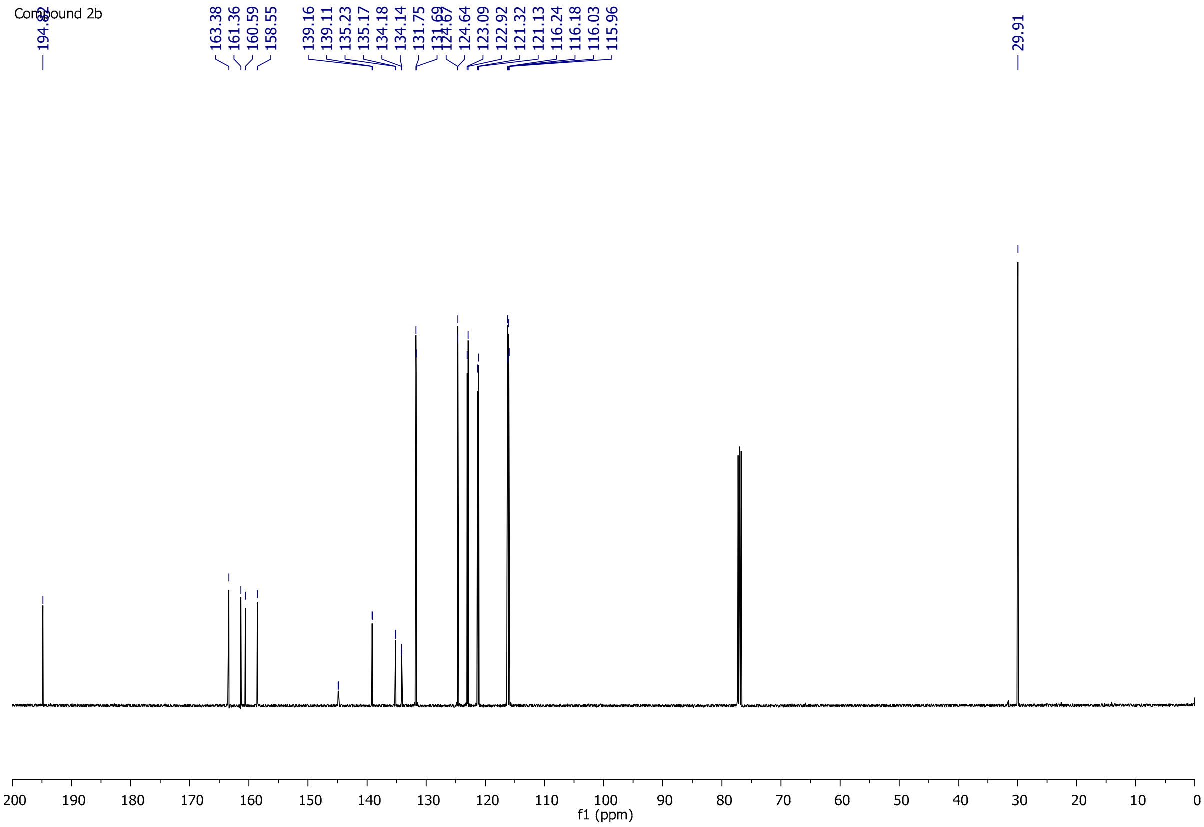

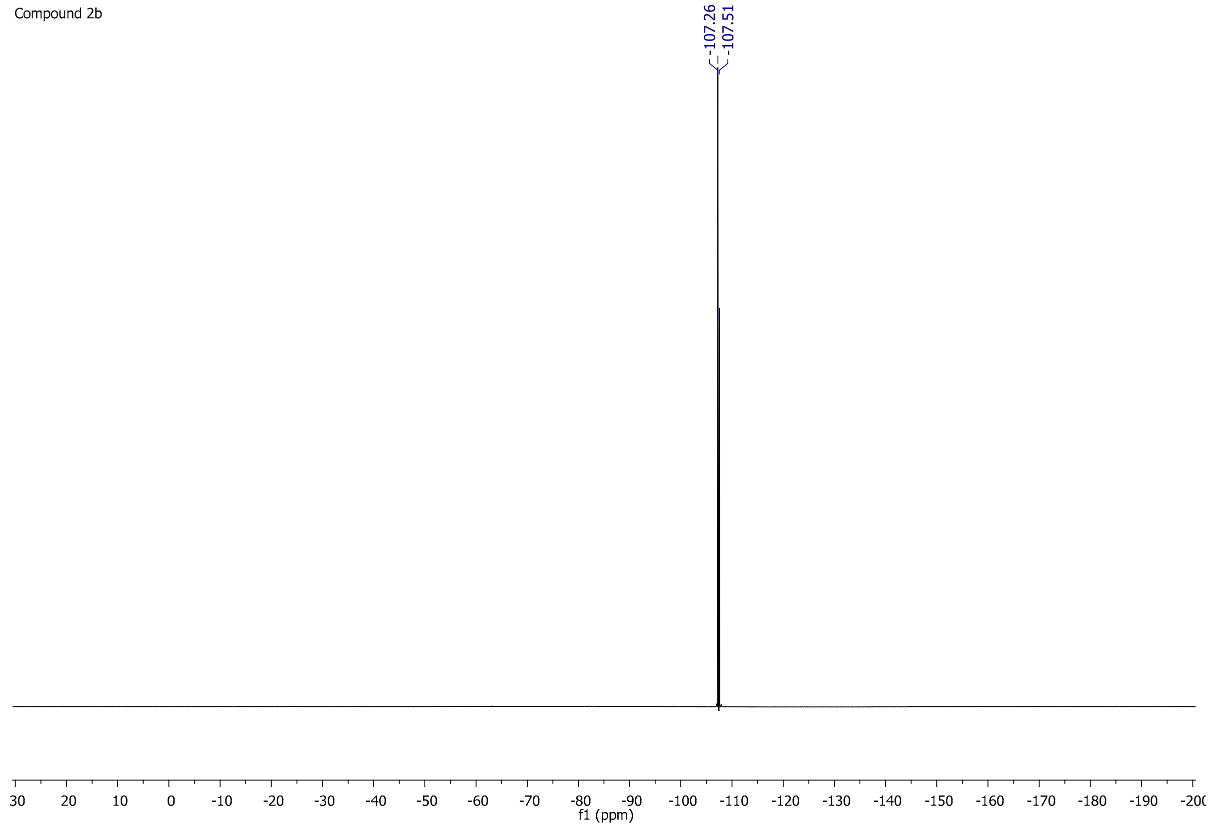

Figure S1.2: ^{1}H-, ^{13}C- and ^{19}F NMR of **2b** in $CDCl_3$ at 500, 125 and 470 MHz, respectively.

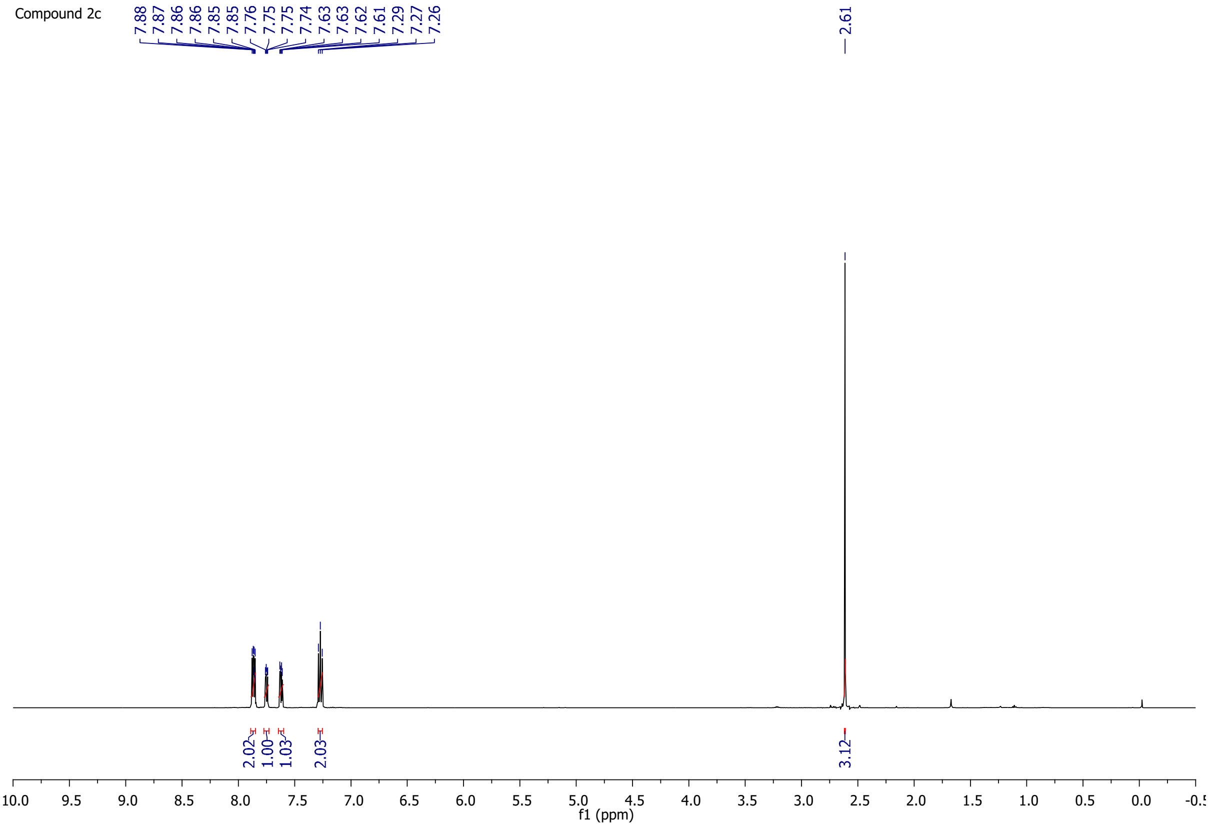
Compound 2c
7.88
7.87
7.86
7.86
7.85
7.76
7.75
7.75
7.74
7.63
7.62
7.61
7.29
7.27
7.26
2.61
2.02
1.00
1.03
2.03
3.12
f1 (ppm)

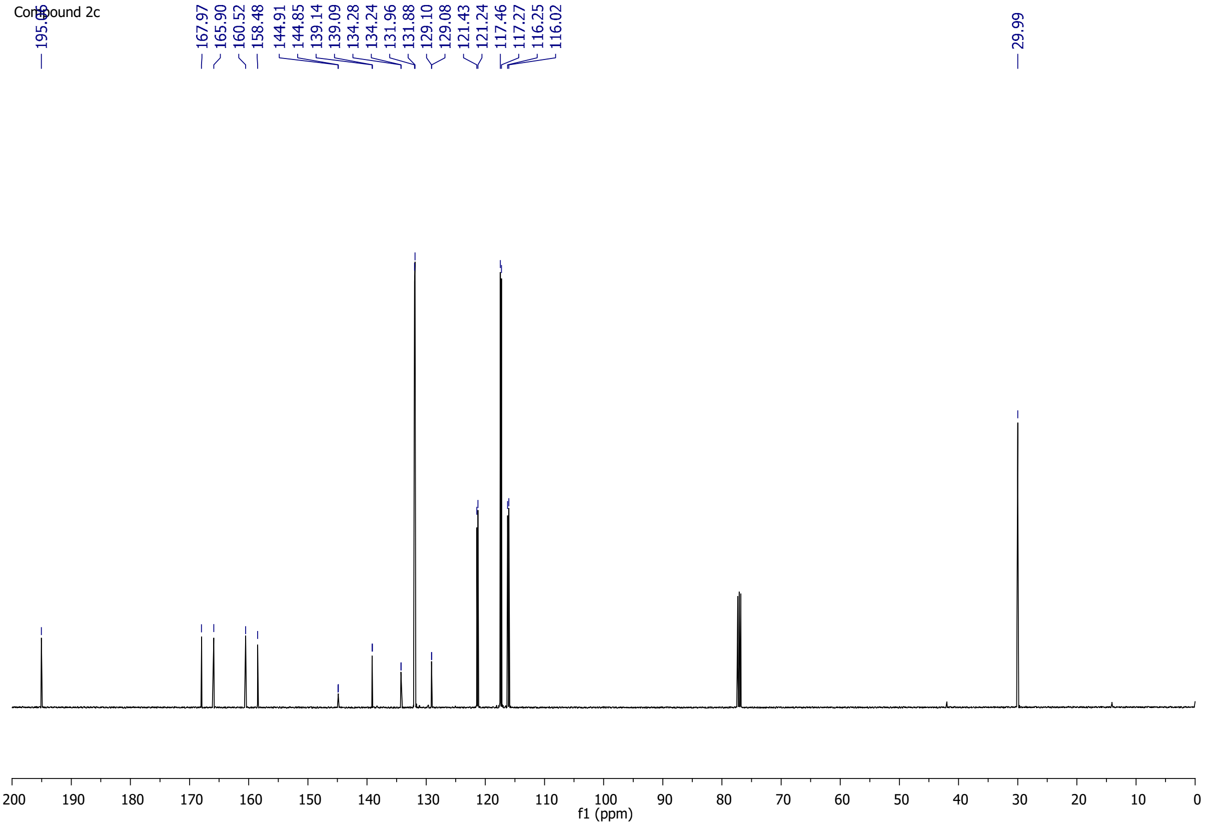
Compound 2c
195.00
167.97
165.90
160.52
158.48
144.91
144.85
139.14
139.09
134.28
134.24
131.96
131.88
129.10
129.08
121.43
121.24
117.46
117.27
116.25
116.02
29.99
f1 (ppm)

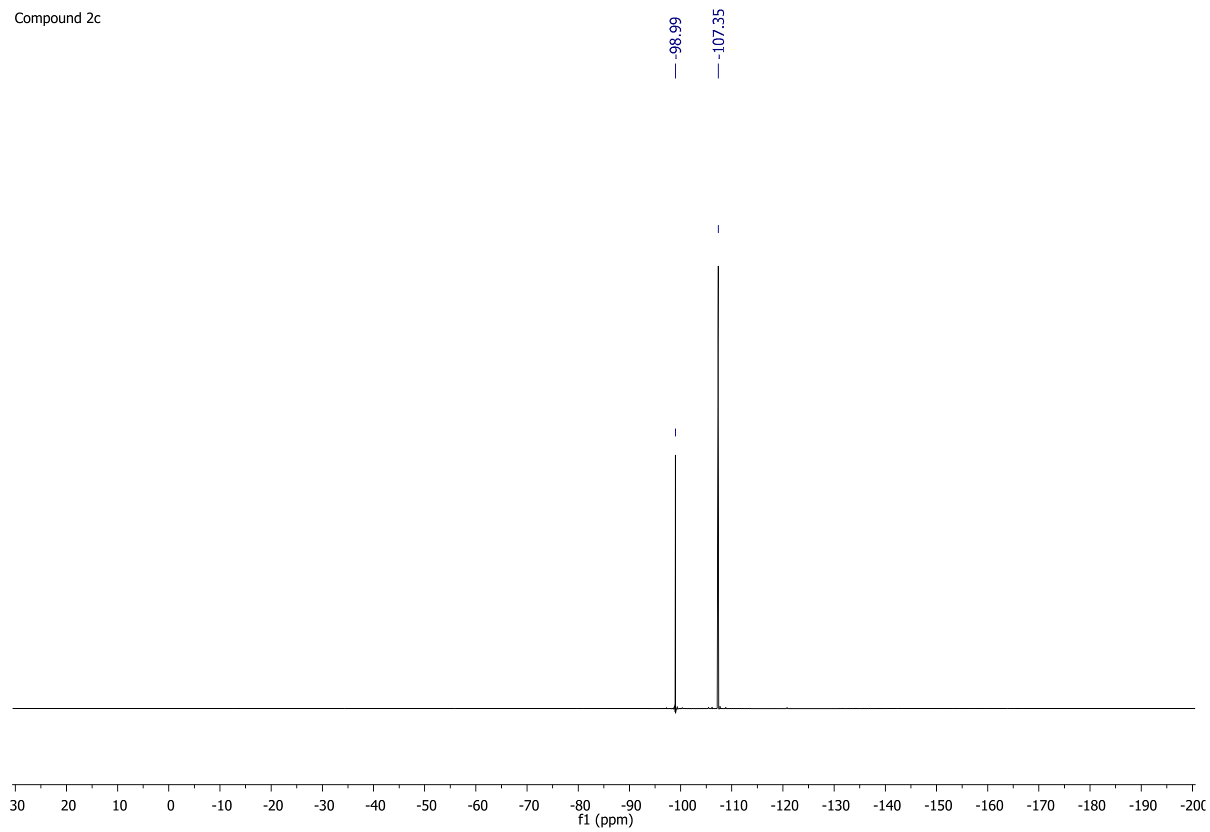

Figure S1.3: ^{1}H-, ^{13}C- and ^{19}F NMR of **2c** in $CDCl_3$ at 500, 125 and 470 MHz, respectively.

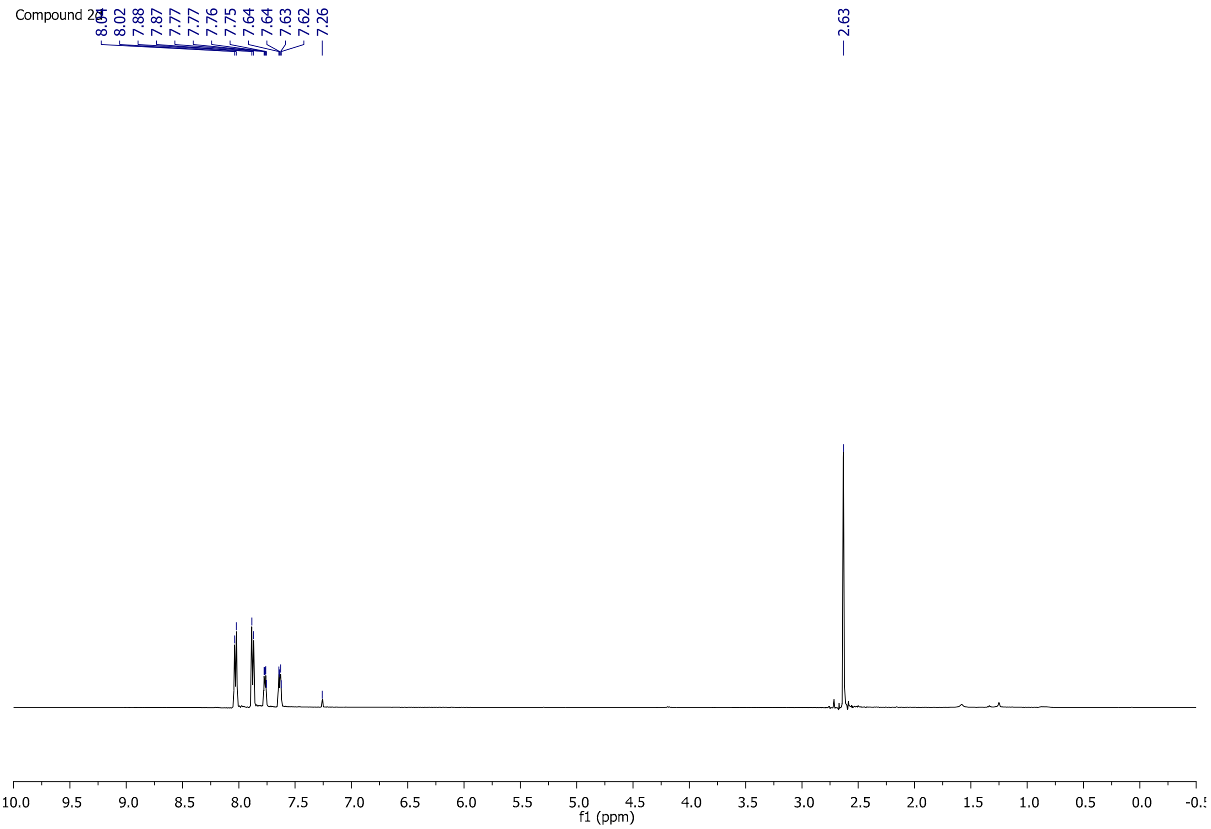

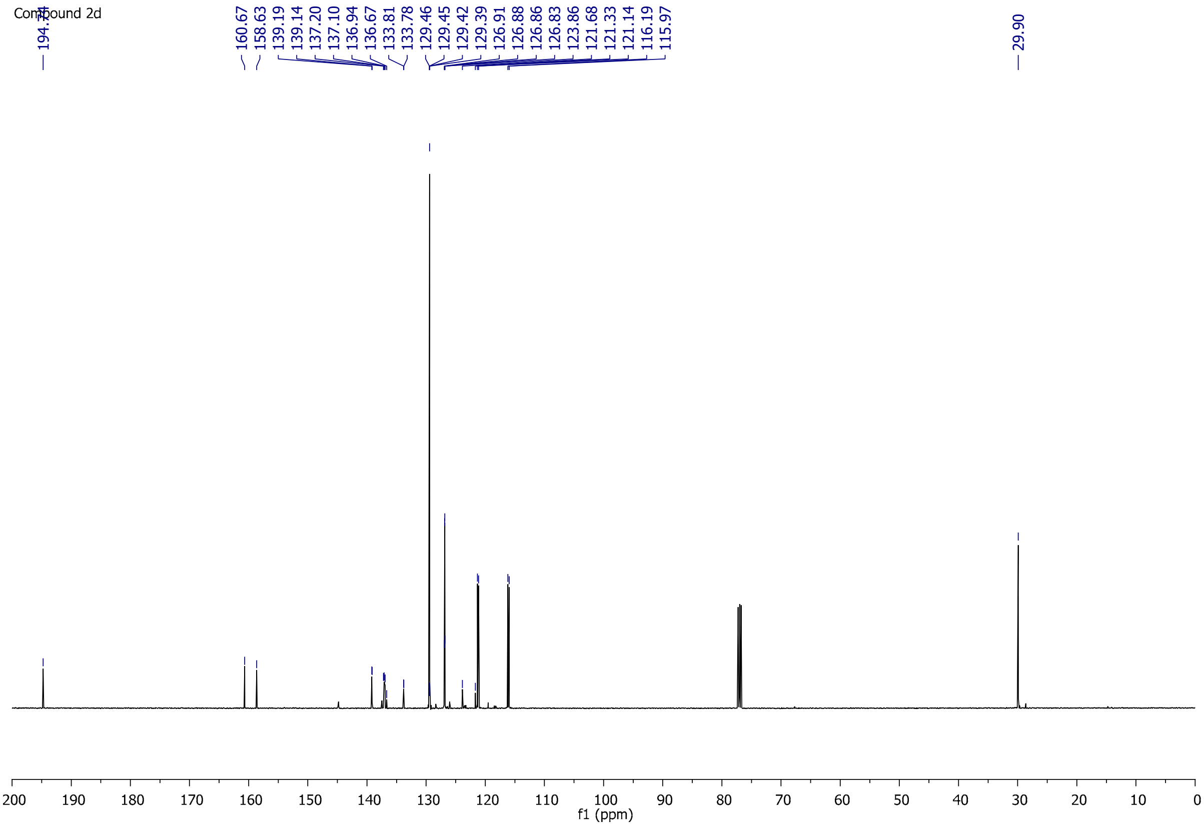

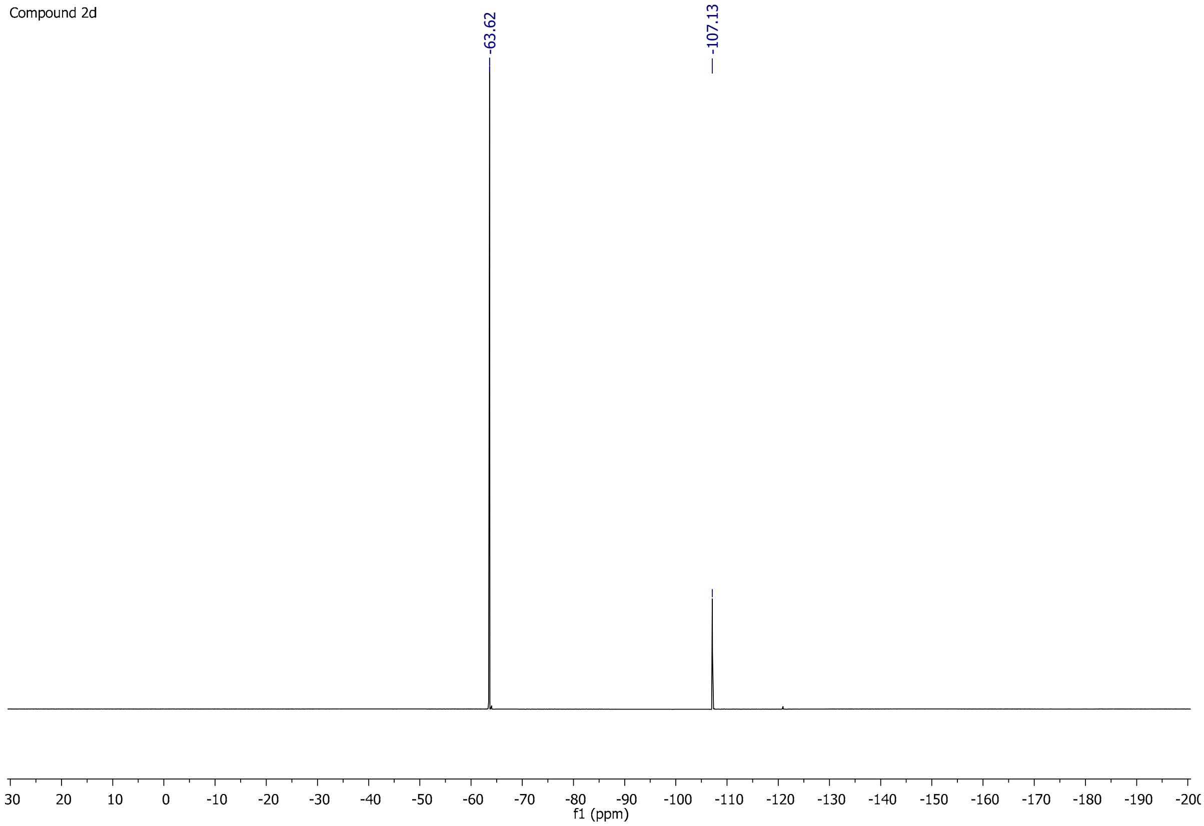

Figure S1.4: ^{1}H-, ^{13}C- and ^{19}F NMR of **2d** in $CDCl_3$ at 500, 125 and 470 MHz, respectively.

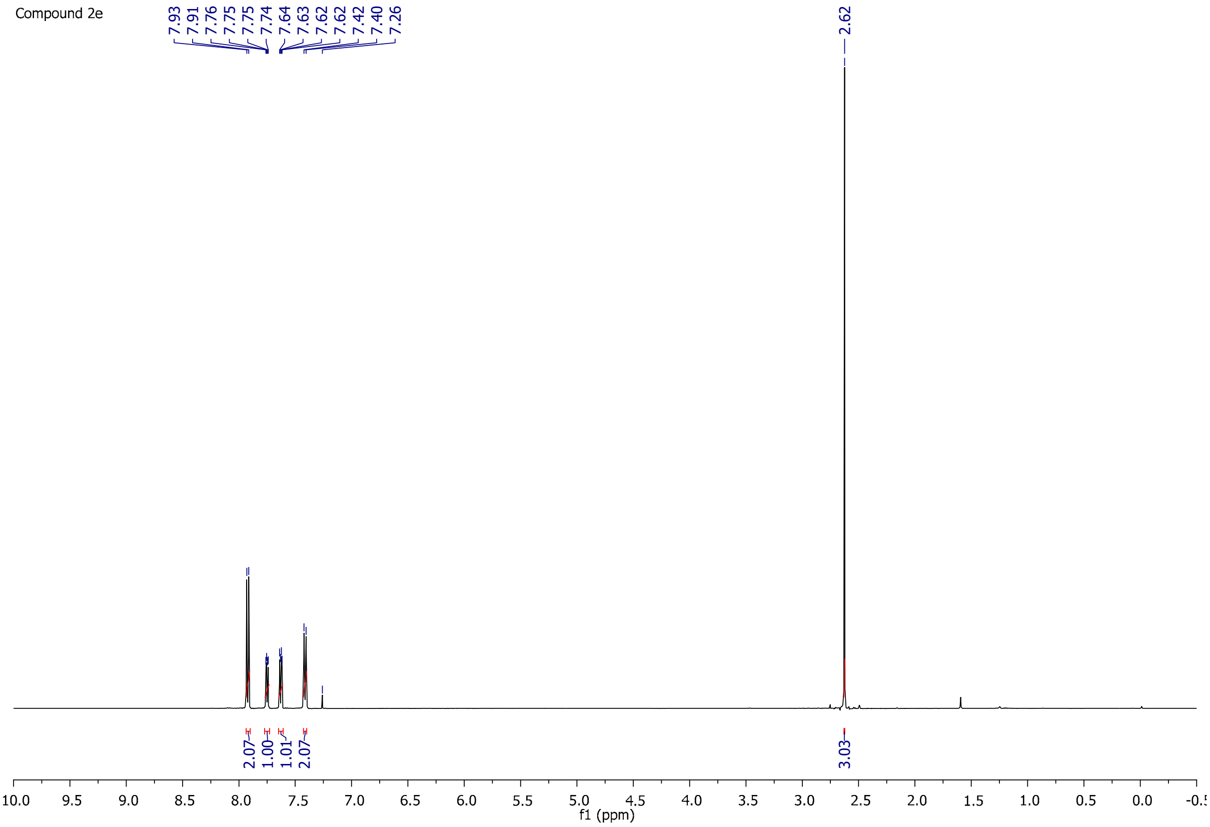
Compound 2e
7.93
7.91
7.76
7.75
7.75
7.74
7.64
7.63
7.62
7.62
7.42
7.40
7.26
2.62
2.07
1.00
1.01
2.07
3.03
10.0 9.5 9.0 8.5 8.0 7.5 7.0 6.5 6.0 5.5 5.0 4.5 4.0 3.5 3.0 2.5 2.0 1.5 1.0 0.5 0.0
f1 (ppm)

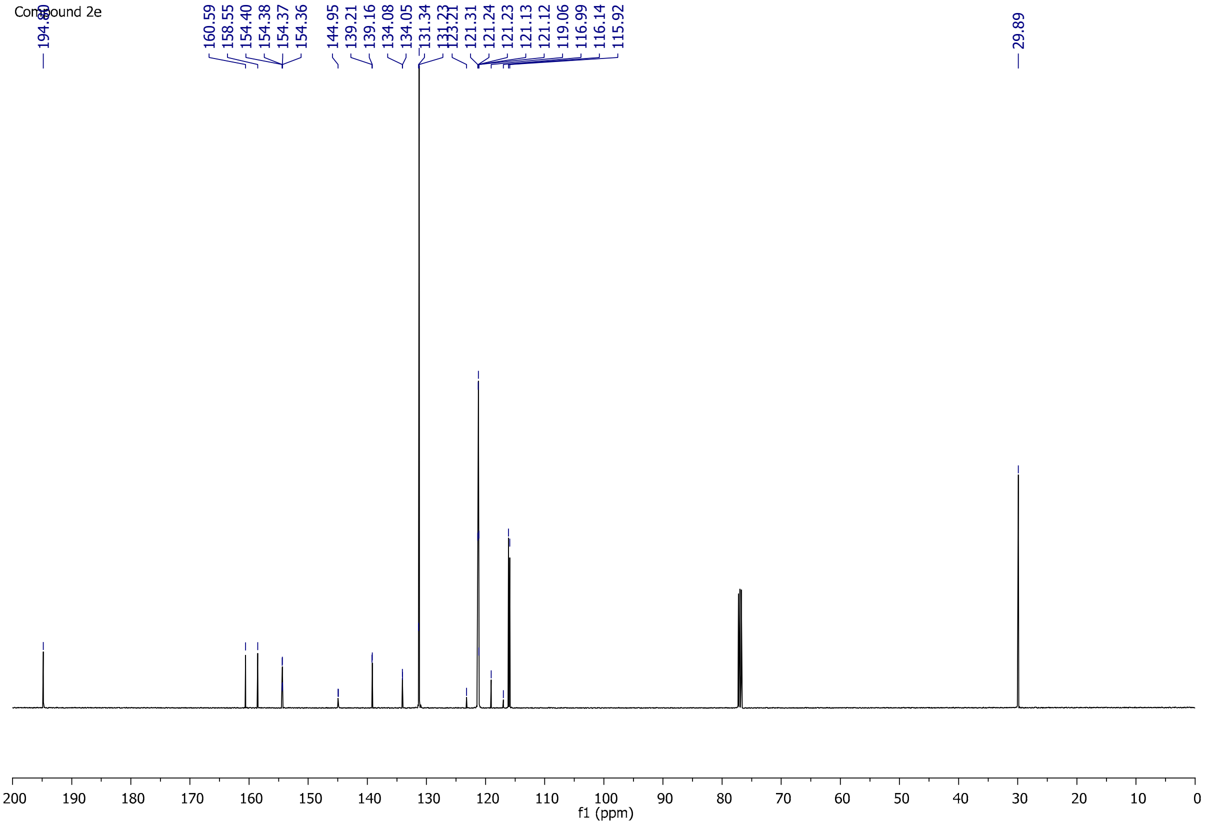
Compound 2e
160.59
158.55
154.40
154.38
154.37
154.36
144.95
139.21
139.16
134.08
134.05
131.34
121.31
121.24
121.23
121.13
121.12
119.06
116.99
116.14
115.92
29.89
200 190 180 170 160 150 140 130 120 110 100 90 80 70 60 50 40 30 20 10 0
f1 (ppm)

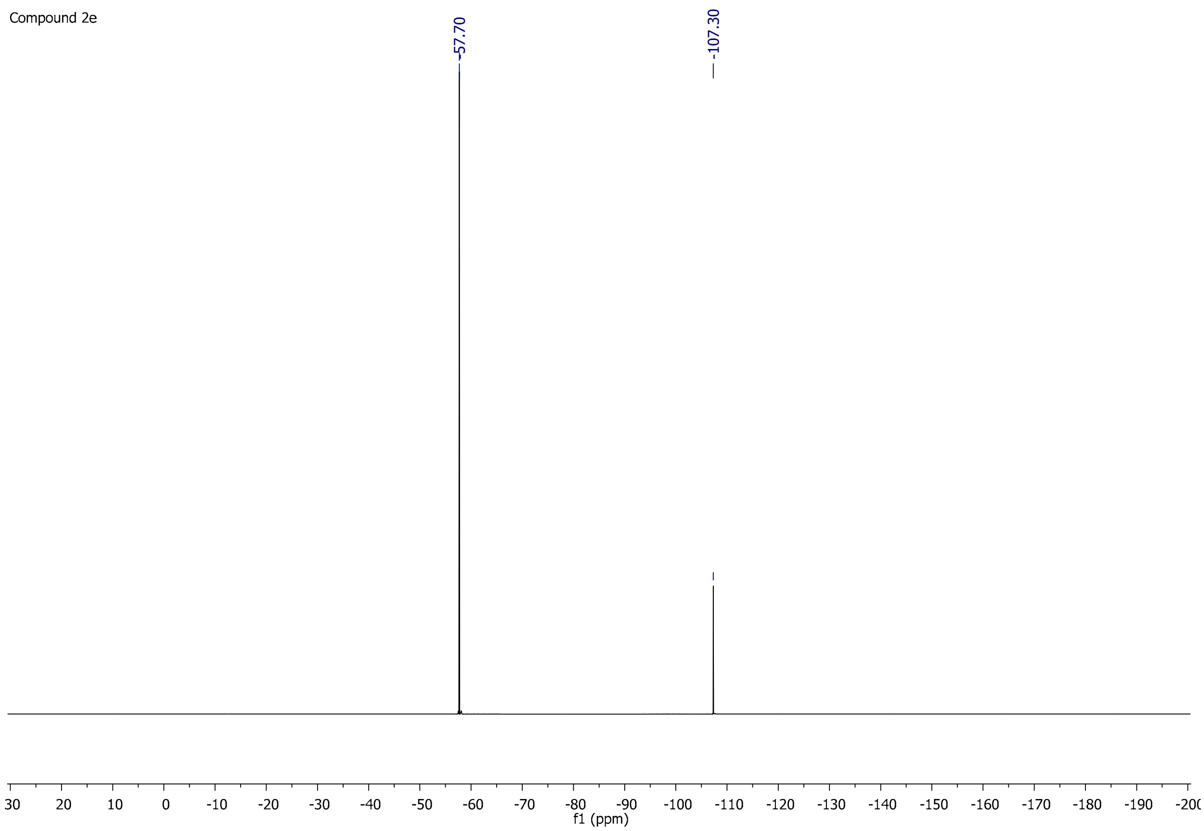

Figure S1.5: ^{1}H-, ^{13}C- and ^{19}F NMR of **2e** in $CDCl_3$ at 500, 125 and 470 MHz, respectively.

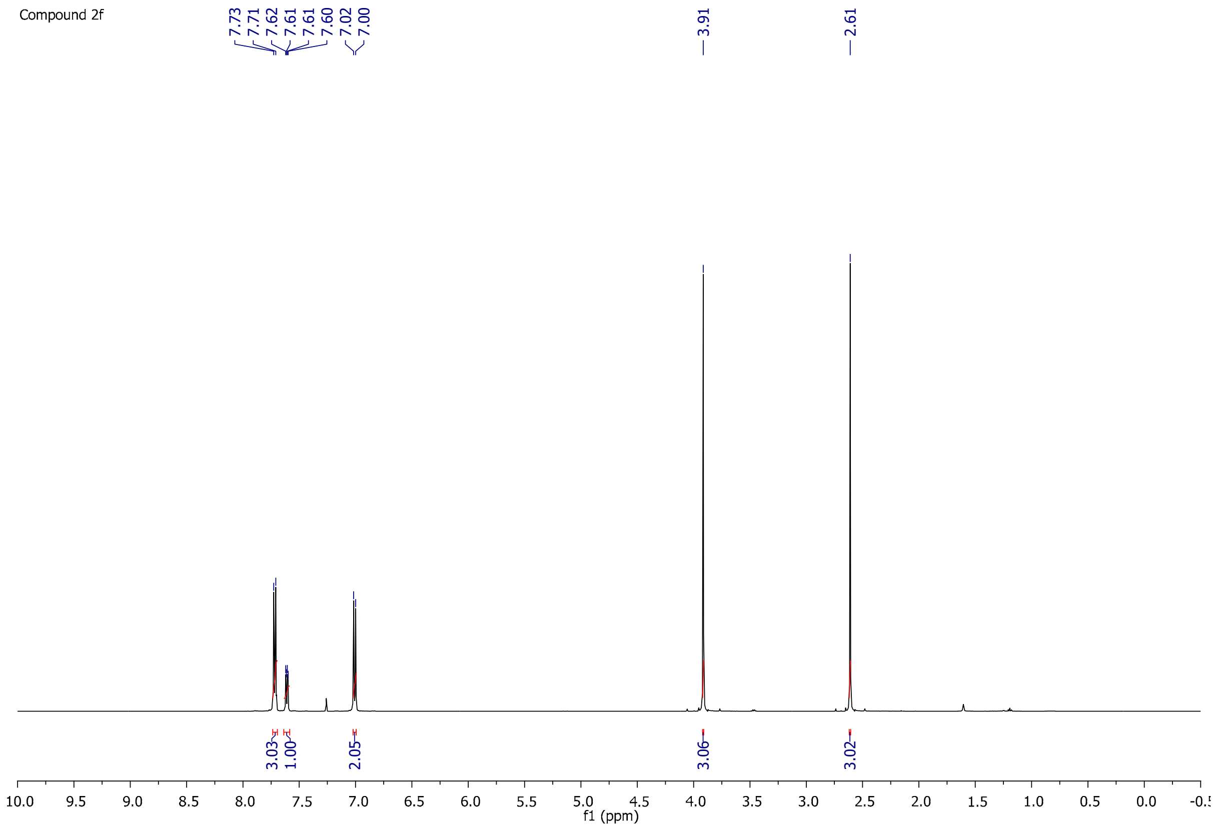

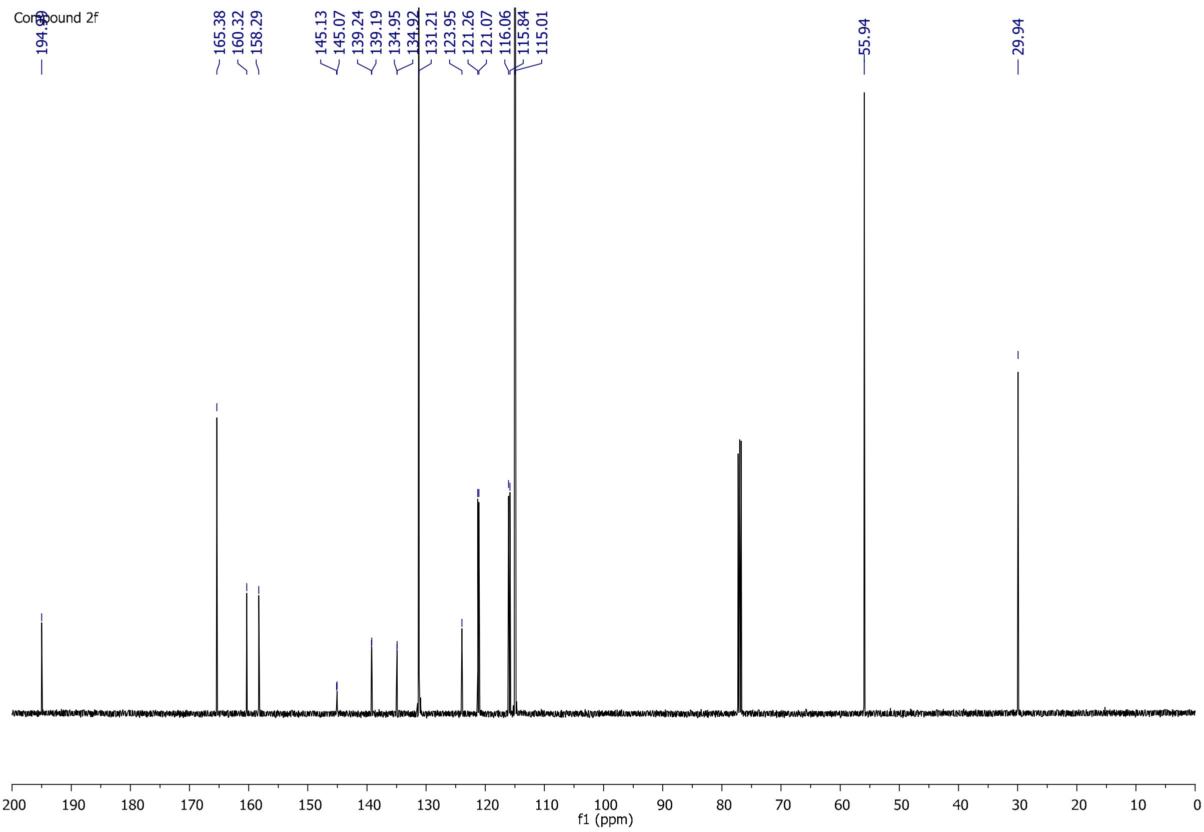

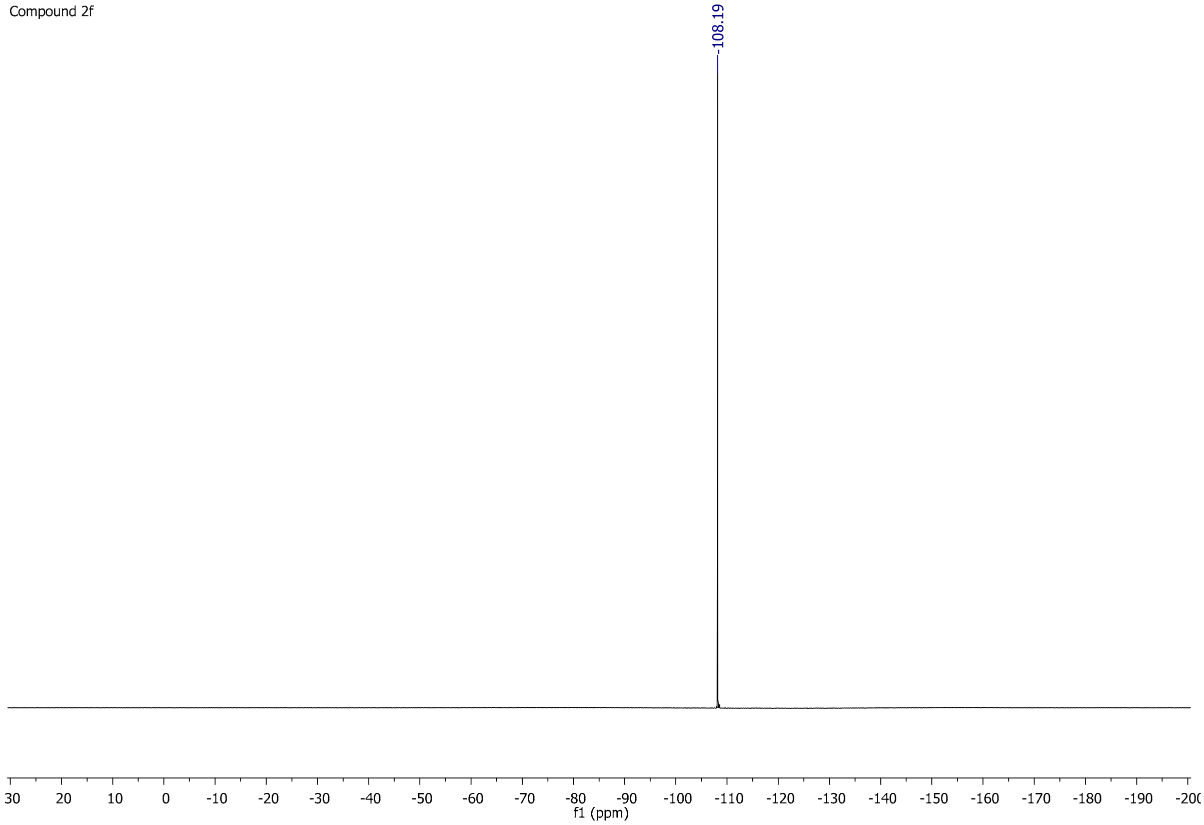

Figure S1.6: ^{1}H-, ^{13}C- and ^{19}F NMR of **2f** in $CDCl_3$ at 500, 125 and 470 MHz, respectively.

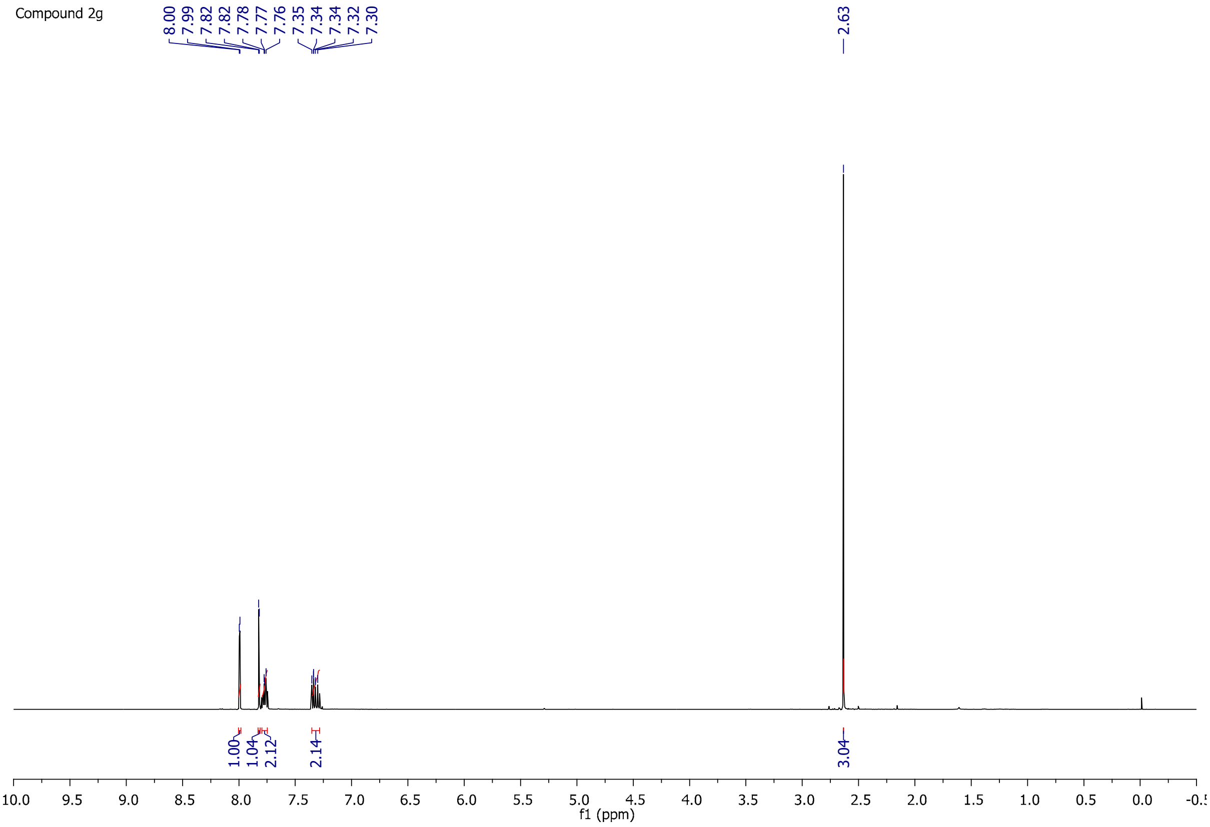
Compound 2g
8.00
7.99
7.82
7.82
7.78
7.77
7.76
7.35
7.34
7.34
7.32
7.30
2.63
1.00
1.04
2.12
2.14
3.04
f1 (ppm)

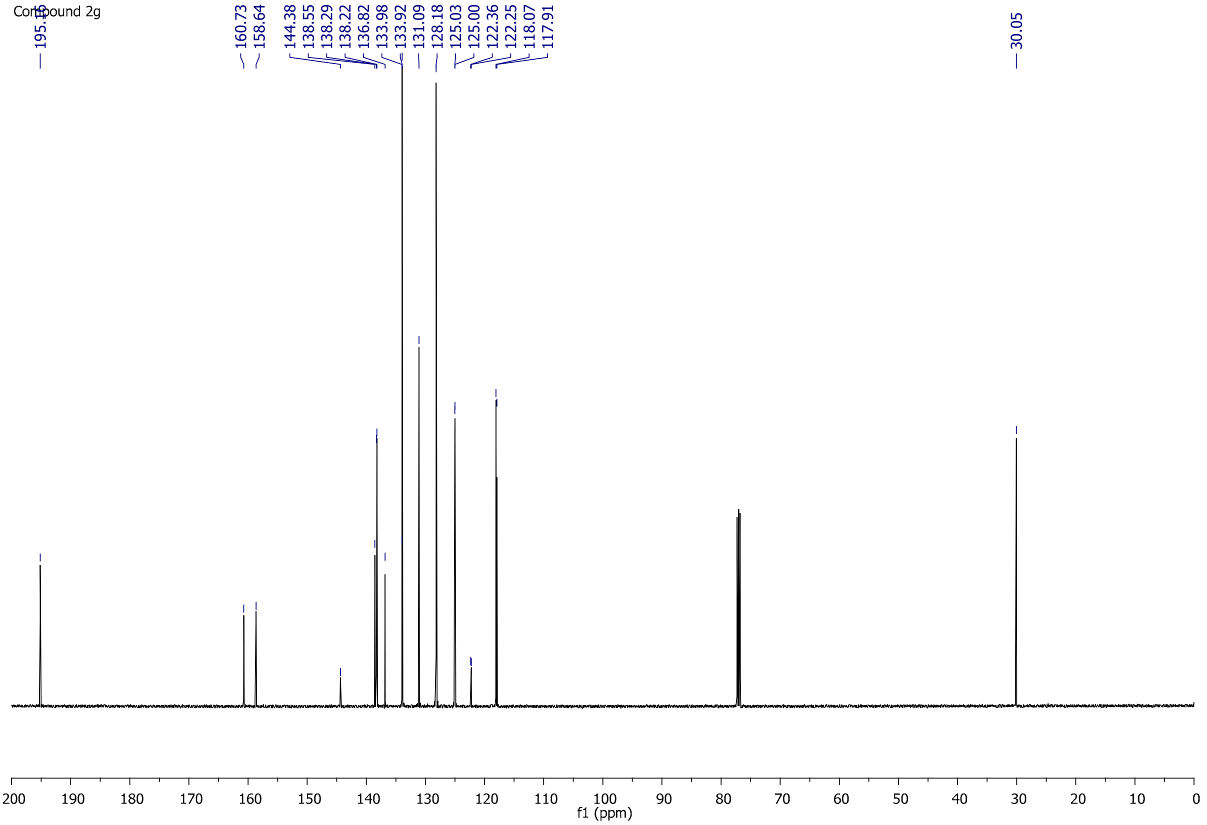
Compound 2g
160.73
158.64
144.38
138.55
138.29
138.22
136.82
133.98
133.92
131.09
128.18
125.03
125.00
122.36
122.25
118.07
117.91
30.05
f1 (ppm)

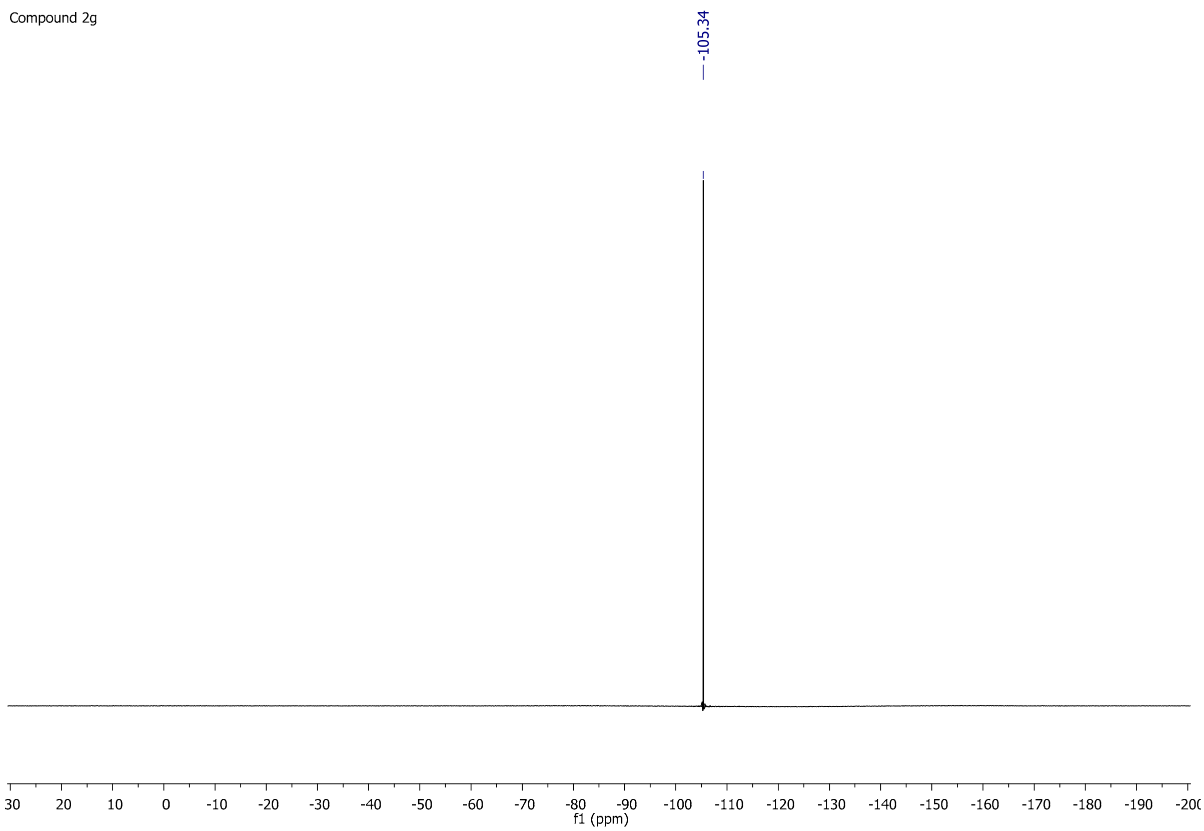

Figure S1.7: ^{1}H-, ^{13}C- and ^{19}F NMR of **2g** in $CDCl_3$ at 500, 125 and 470 MHz, respectively.

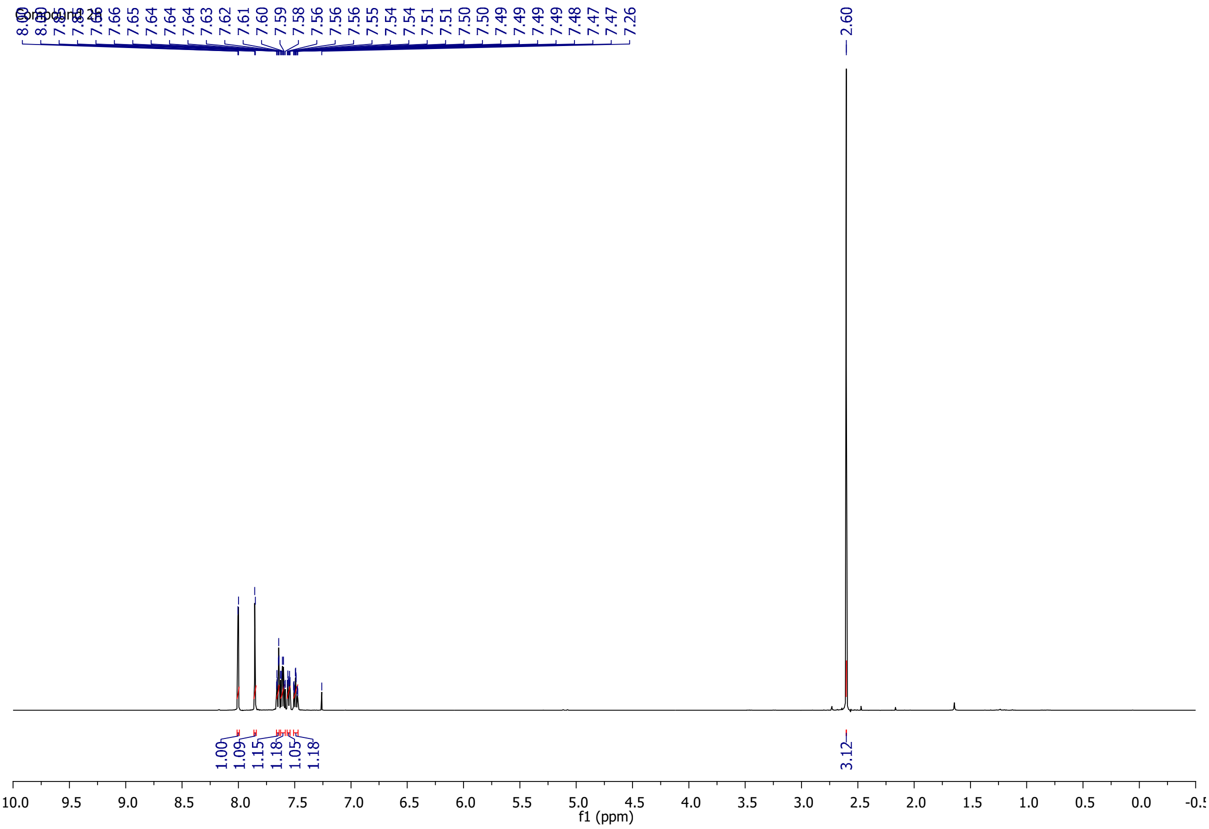

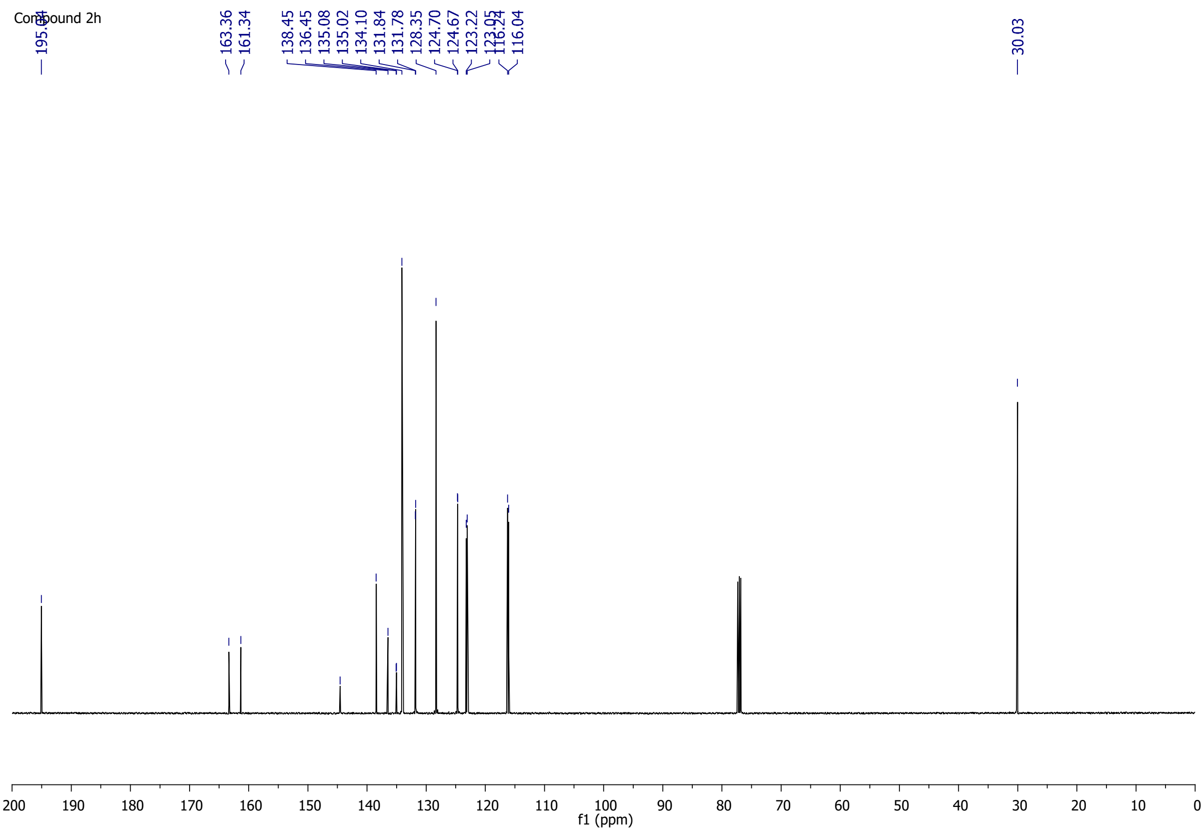

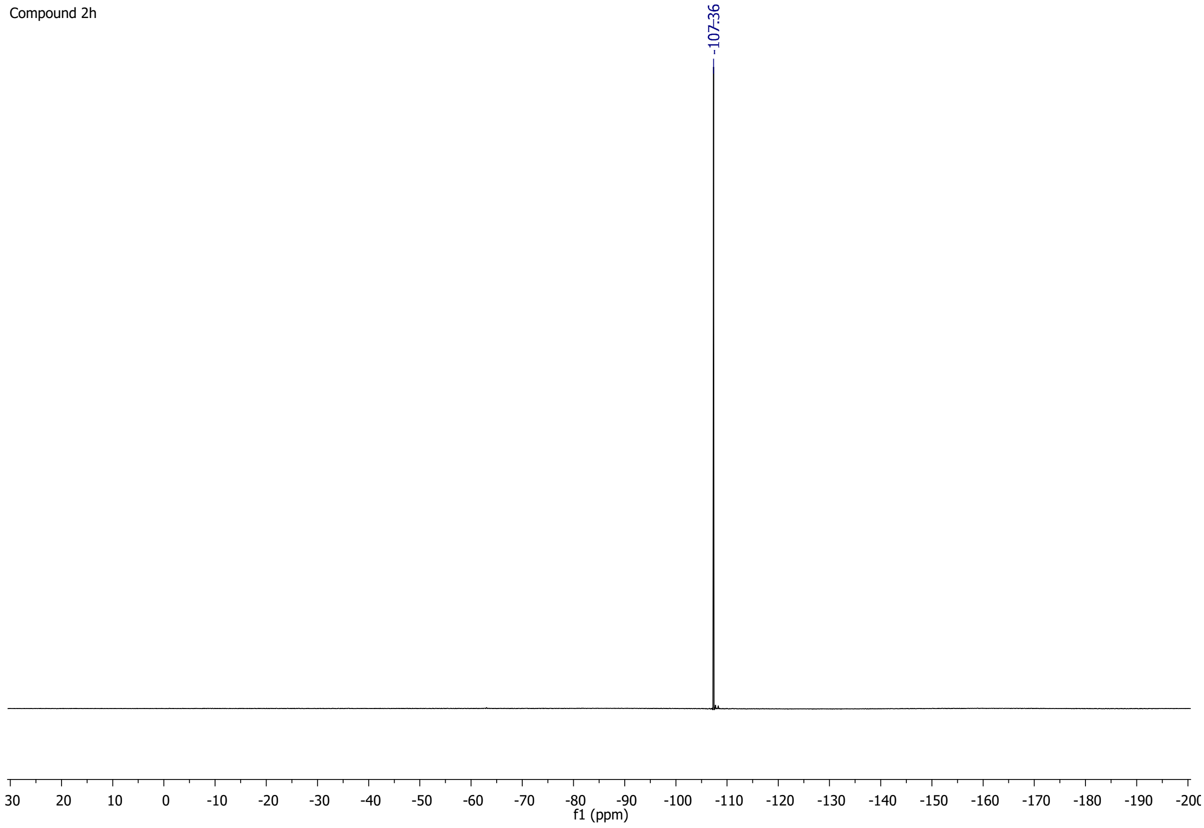

Figure S1.8: ^{1}H-, ^{13}C- and ^{19}F NMR of **2h** in $CDCl_3$ at 500, 125 and 470 MHz, respectively.

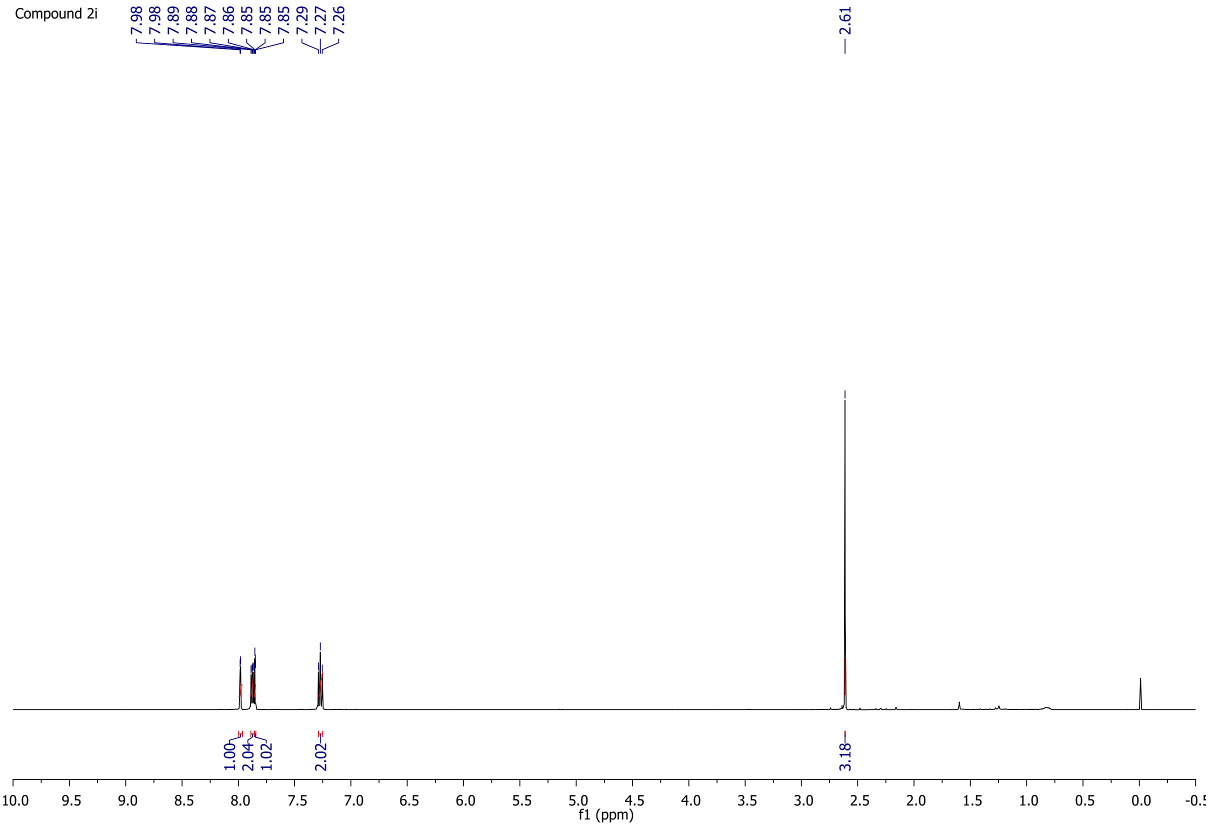
Compound 2i
7.98
7.98
7.89
7.88
7.87
7.86
7.85
7.85
7.85
7.29
7.27
7.26
2.61
1.00
2.04
1.02
2.02
3.18
10.0 9.5 9.0 8.5 8.0 7.5 7.0 6.5 6.0 5.5 5.0 4.5 4.0 3.5 3.0 2.5 2.0 1.5 1.0 0.5 0.0
f1 (ppm)

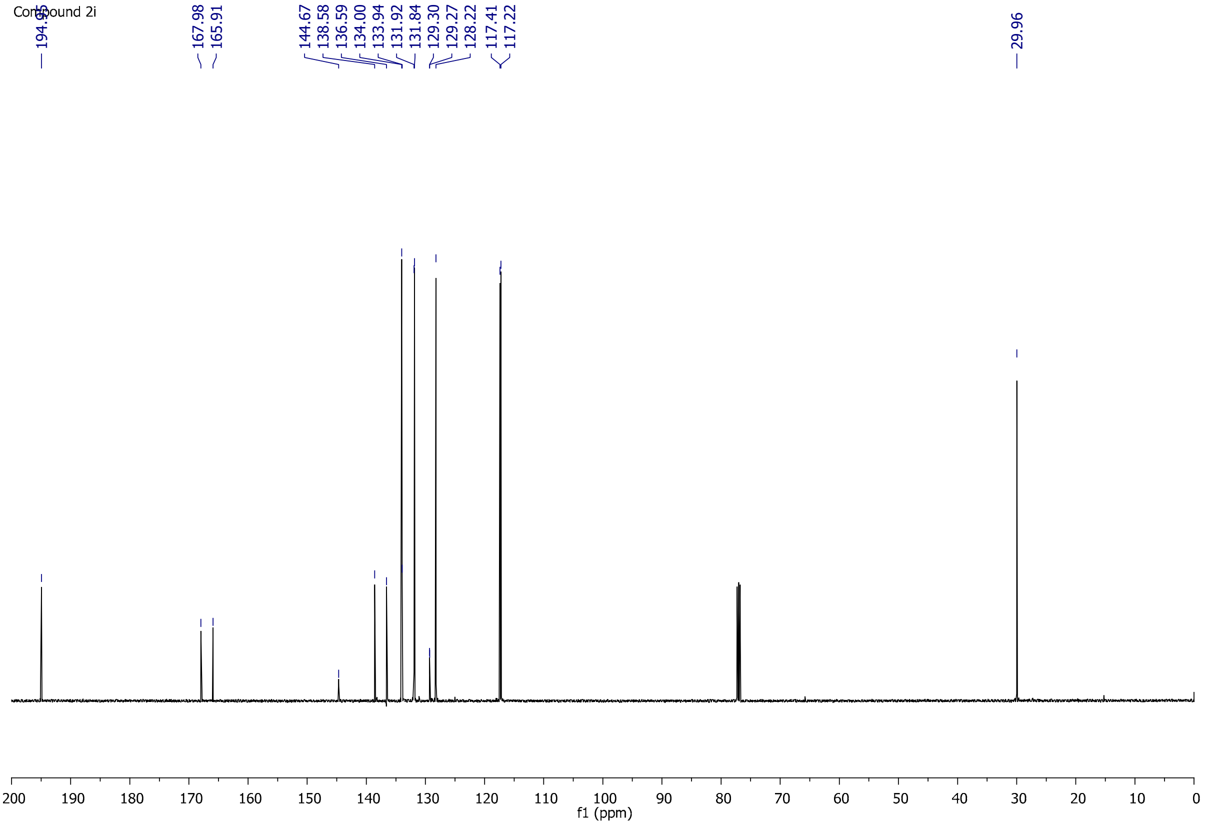
Compound 2i
167.98
165.91
144.67
138.58
136.59
134.00
133.94
131.92
131.84
129.30
129.27
128.22
117.41
117.22
29.96
200 190 180 170 160 150 140 130 120 110 100 90 80 70 60 50 40 30 20 10 0
f1 (ppm)

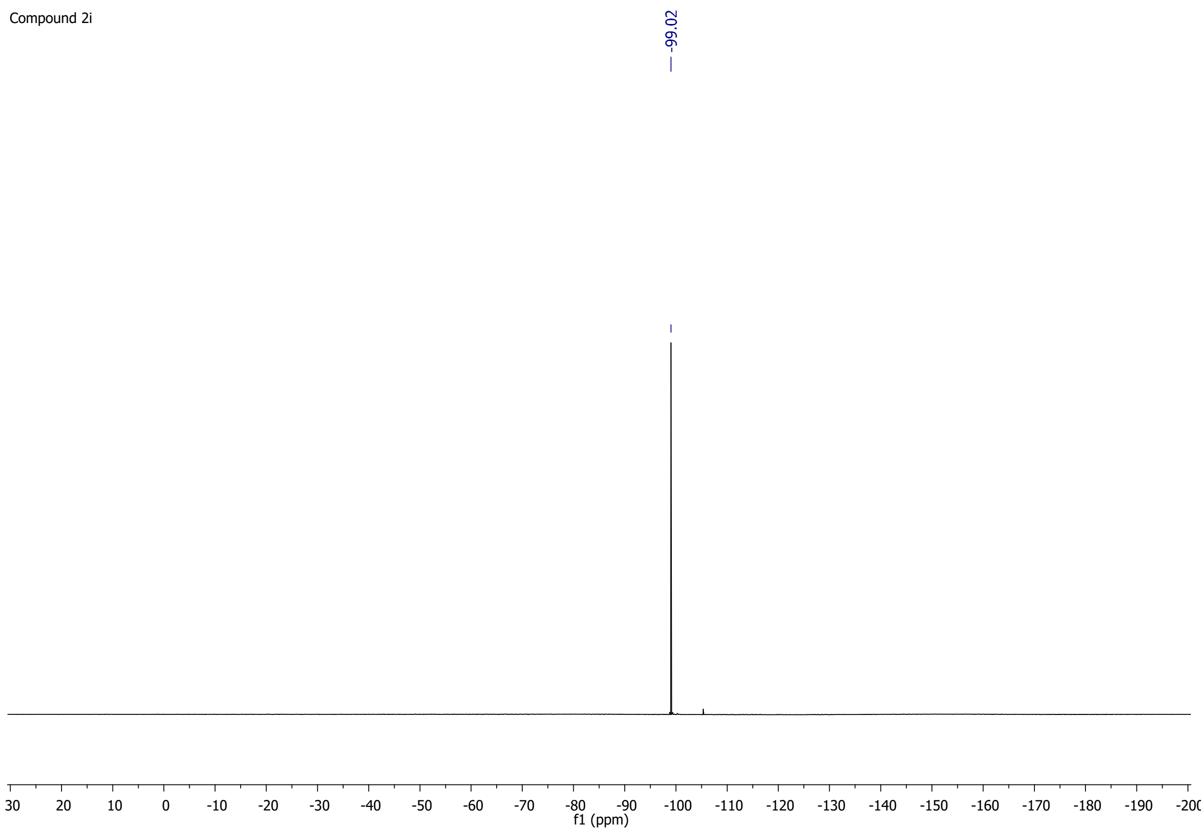

Figure S1.9: ^{1}H-, ^{13}C- and ^{19}F NMR of **2i** in $CDCl_3$ at 500, 125 and 470 MHz, respectively.

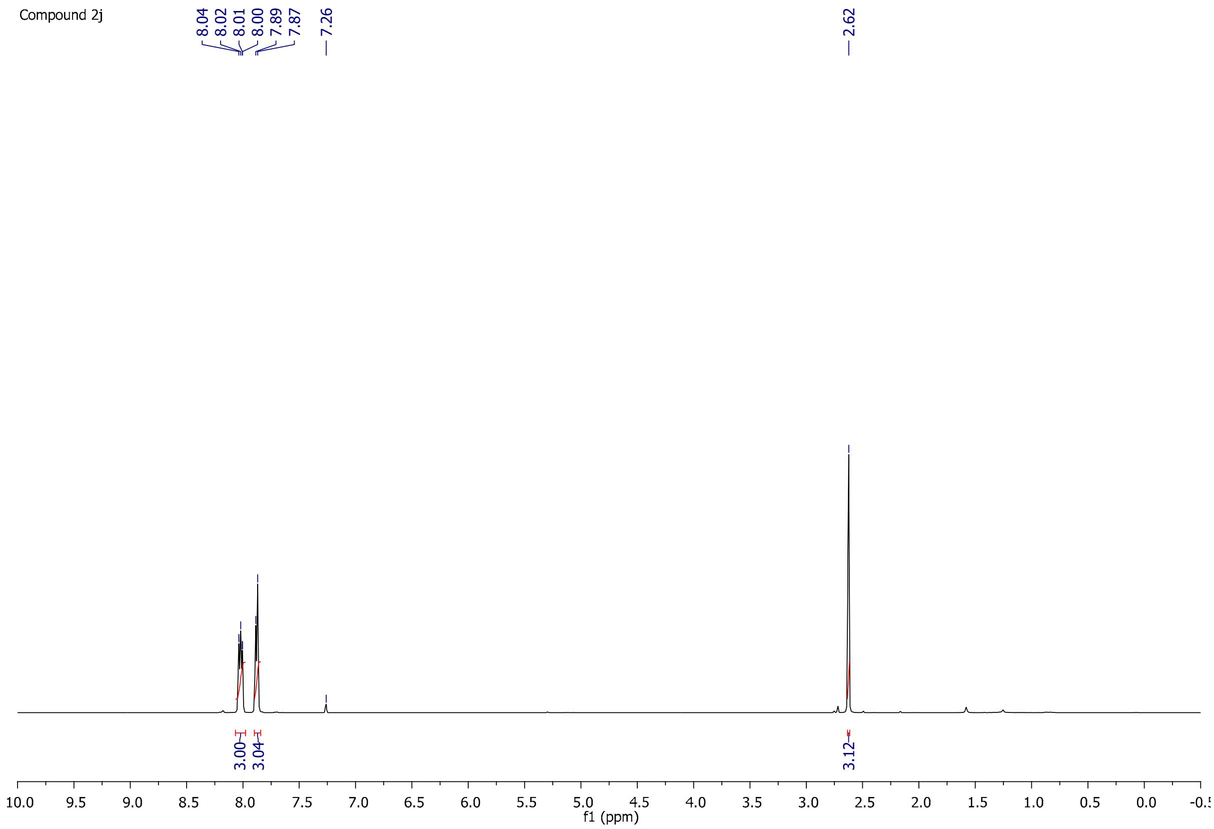

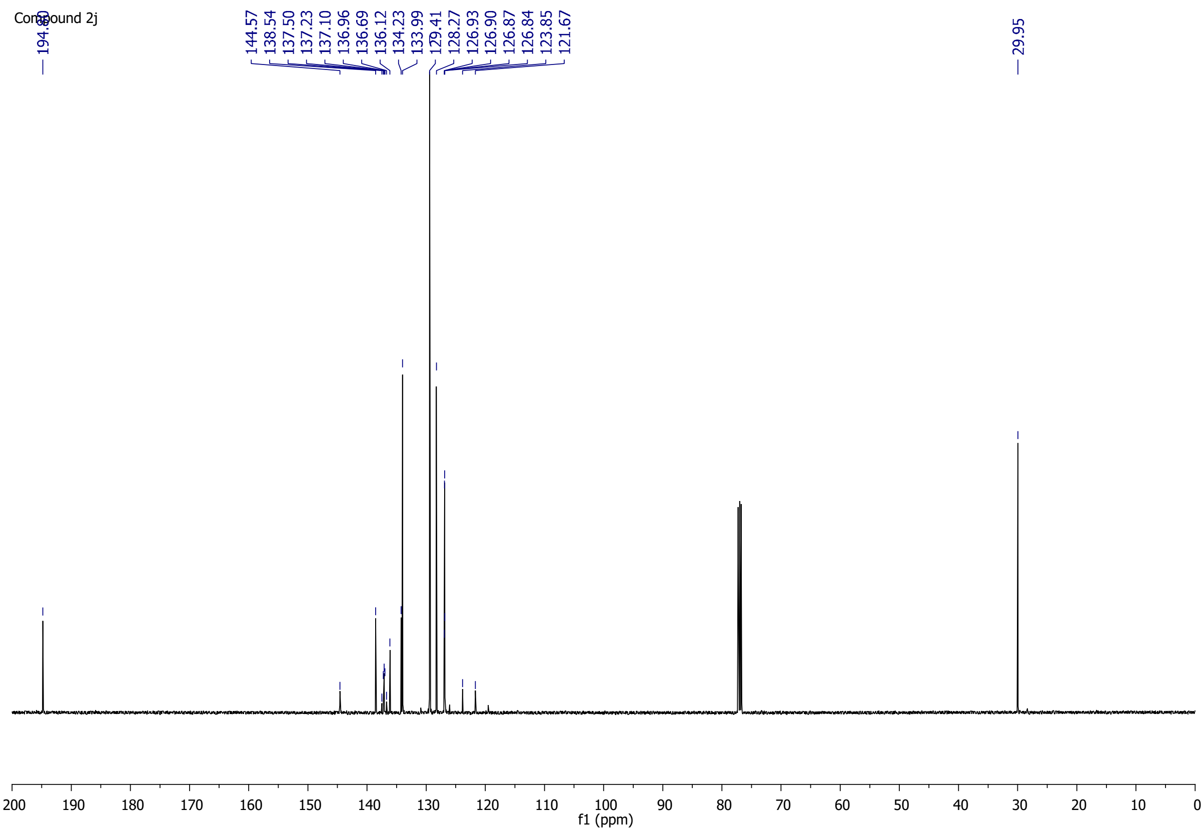

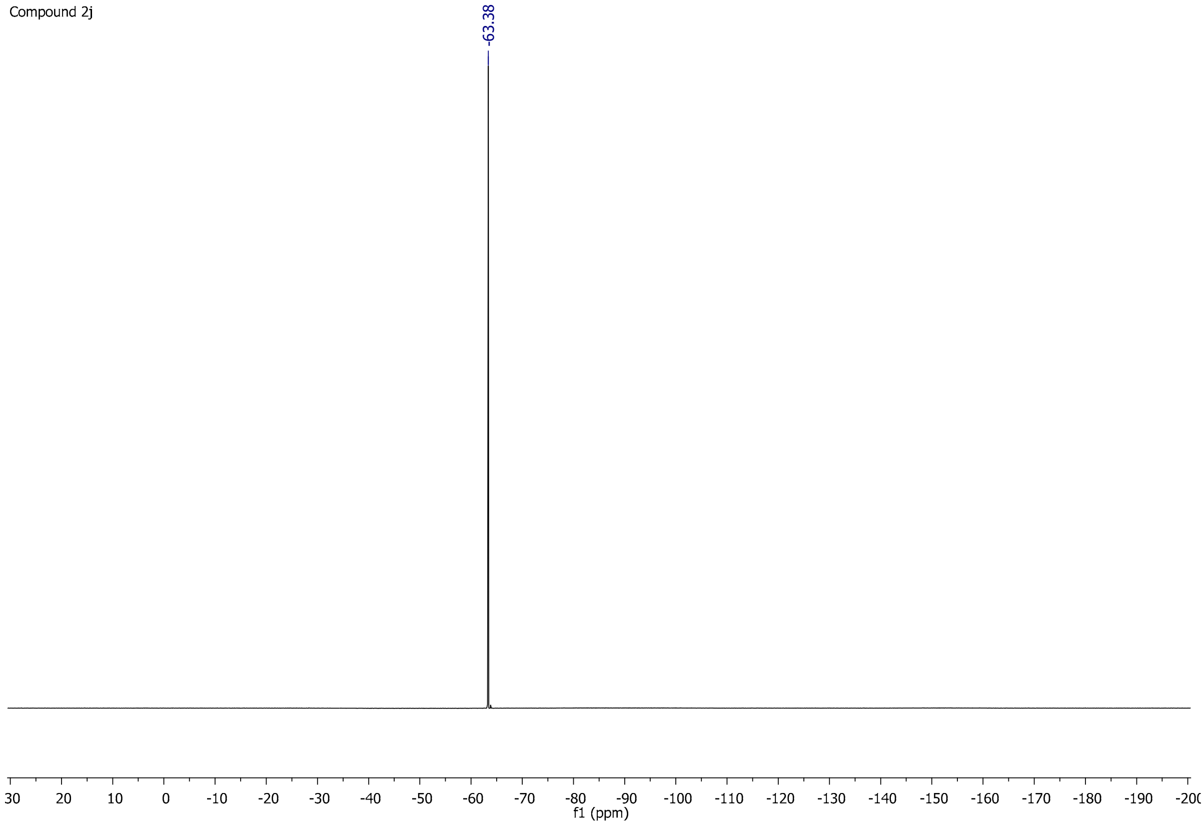

Figure S1.10: ^{1}H-, ^{13}C- and ^{19}F NMR of **2j** in $CDCl_3$ at 500, 125 and 470 MHz, respectively.

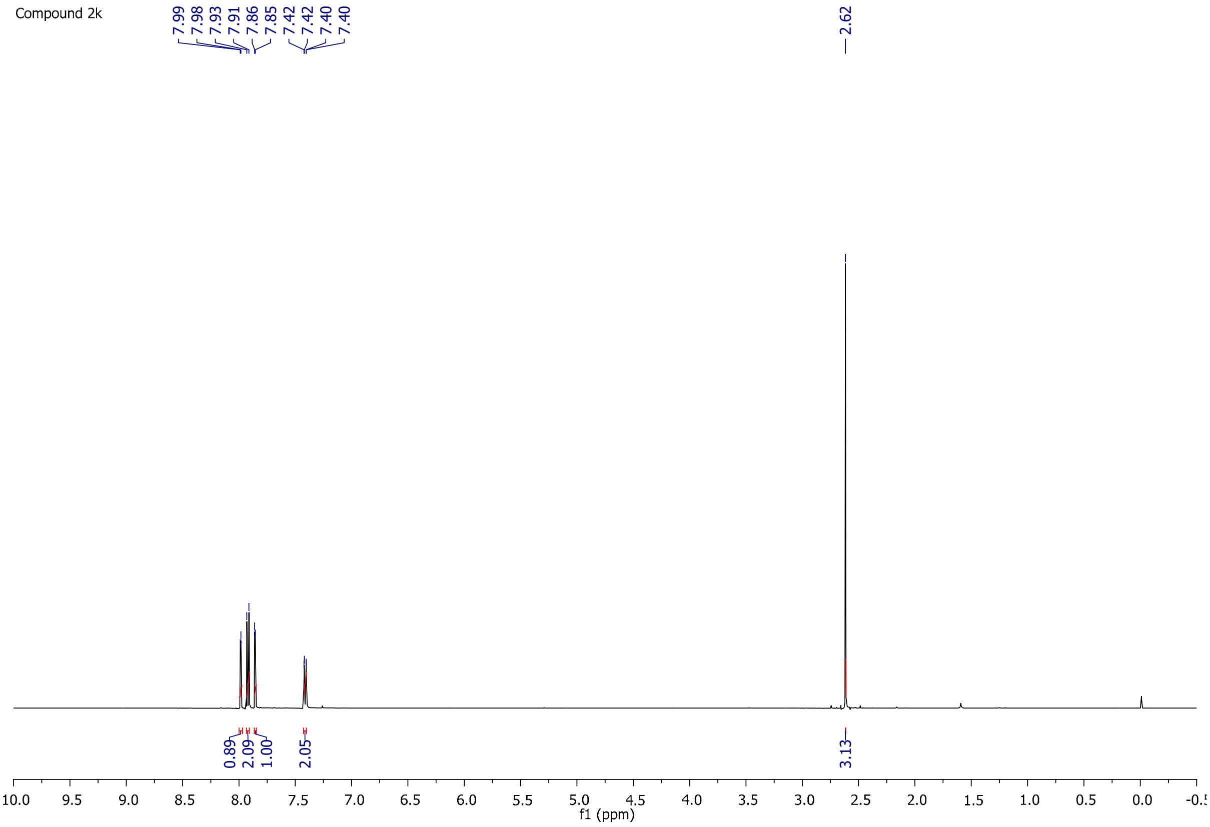
Compound 2k
7.99
7.98
7.93
7.91
7.86
7.85
7.42
7.42
7.40
7.40
2.62
0.89
2.09
1.00
2.05
3.13
f1 (ppm)

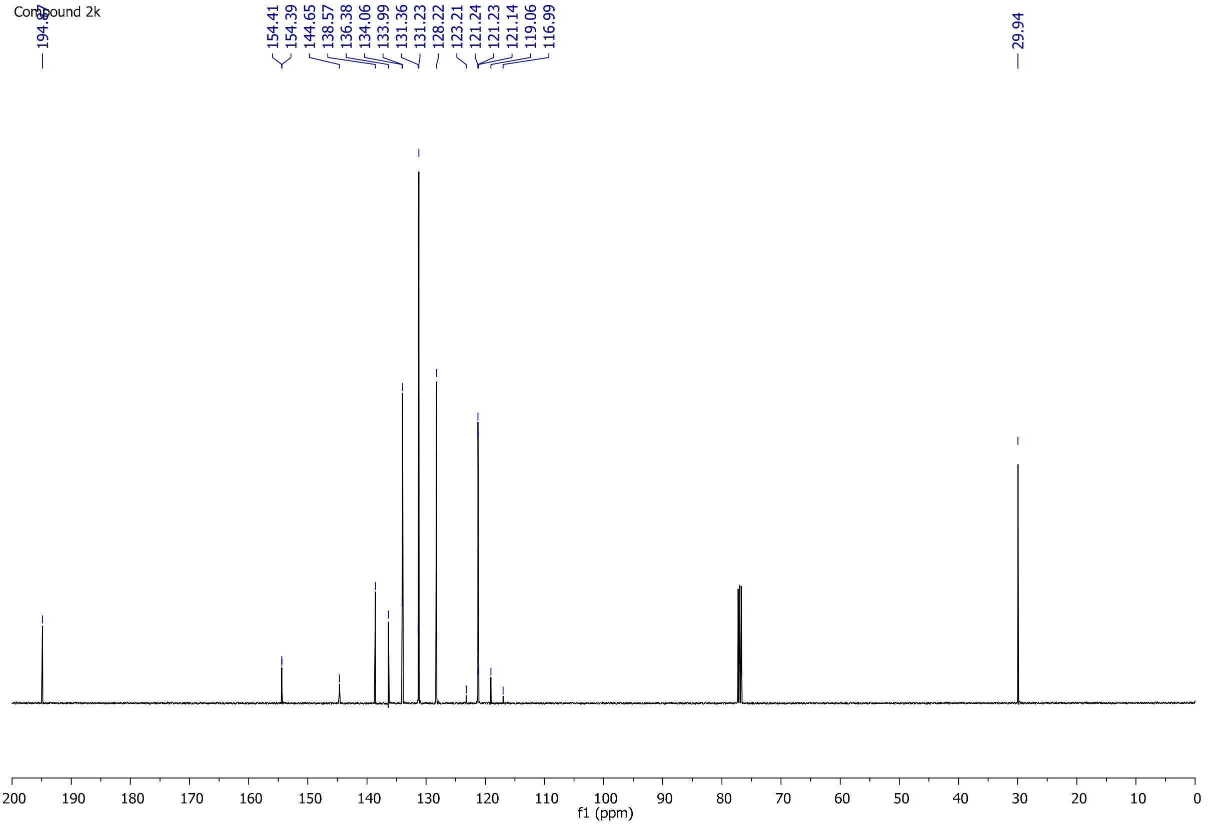
Compound 2k
194.87
154.41
154.39
144.65
138.57
136.38
134.06
133.99
131.36
131.23
128.22
123.21
121.24
121.23
121.14
119.06
116.99
29.94
f1 (ppm)

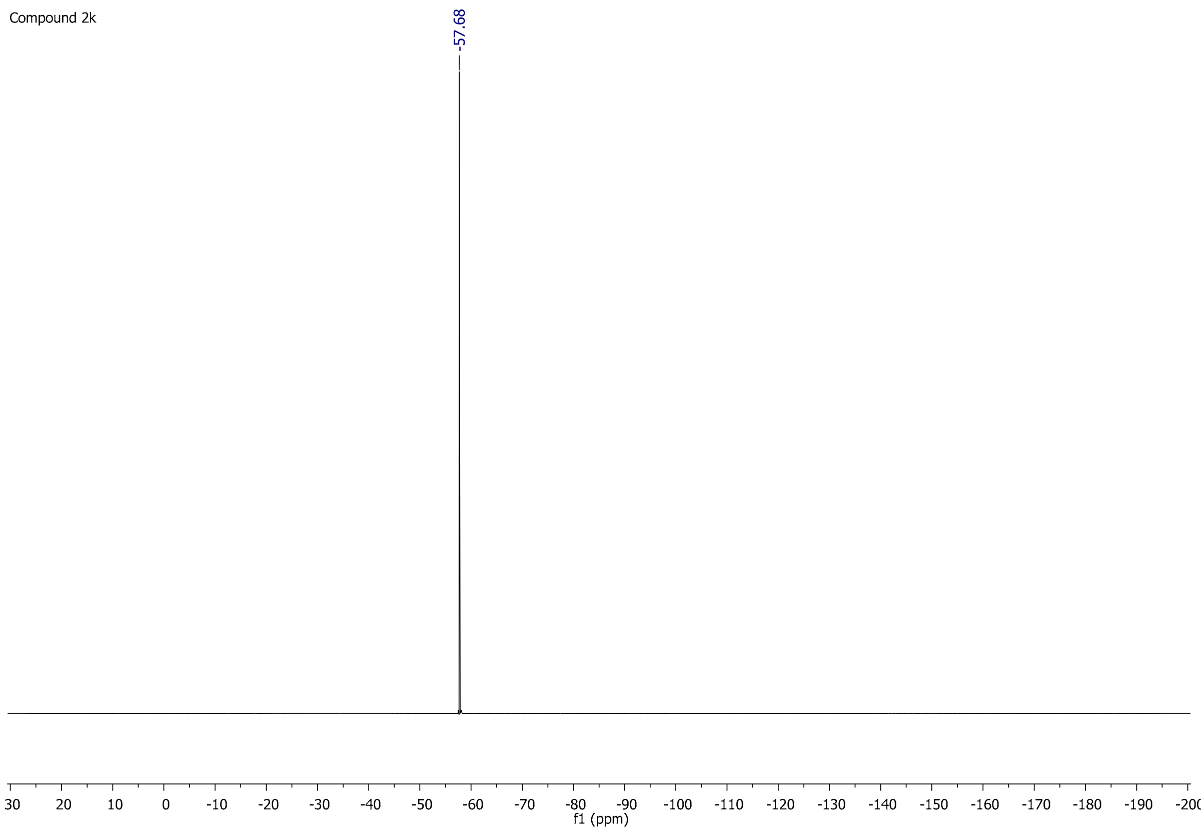

Figure S1.11: ^{1}H-, ^{13}C- and ^{19}F NMR of **2k** in $CDCl_3$ at 500, 125 and 470 MHz, respectively.

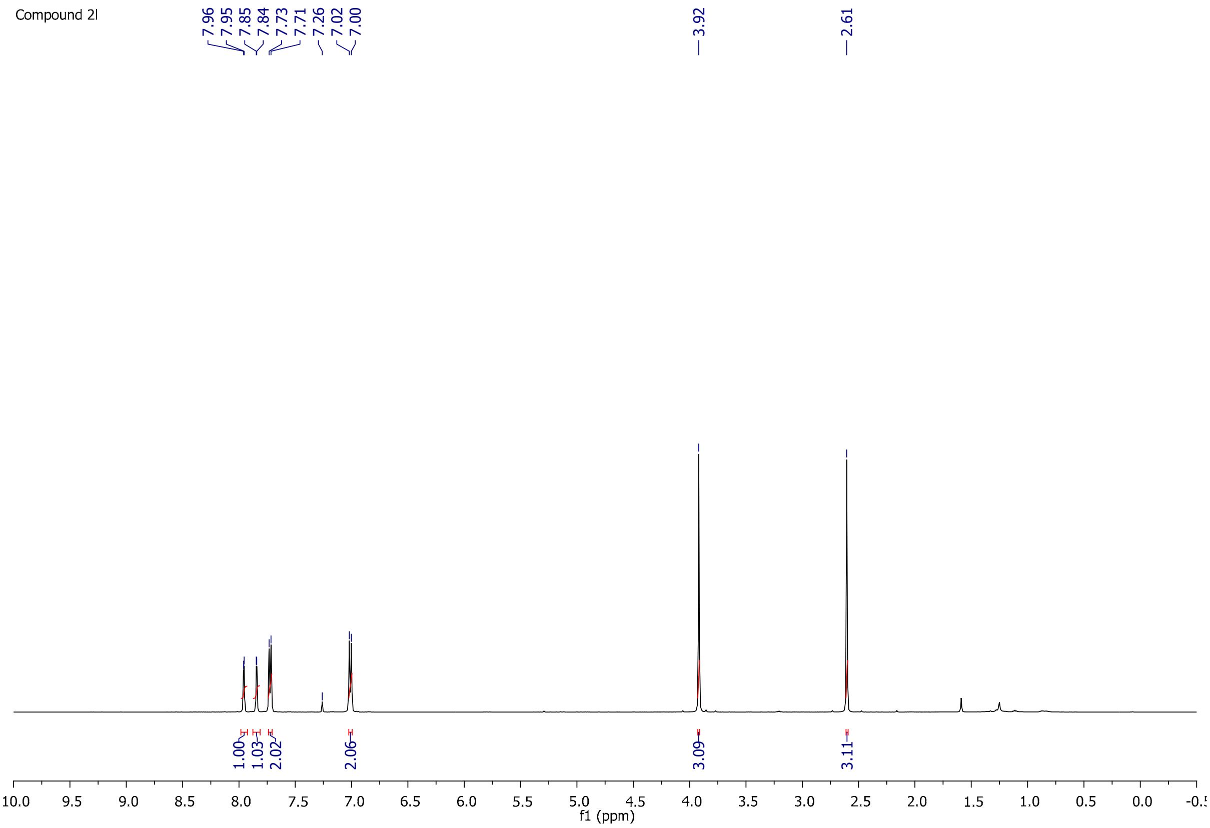

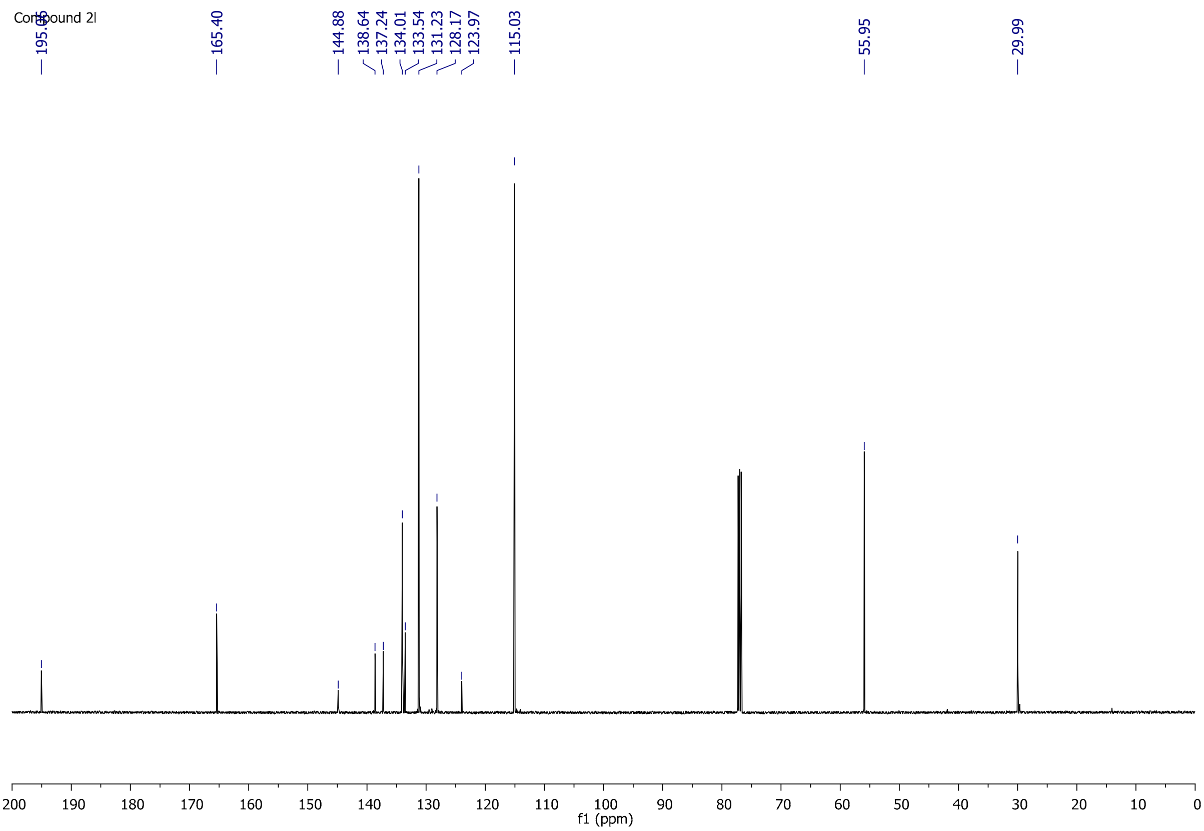

Figure S1.12: ^{1}H- and ^{13}C-NMR of **2l** in $CDCl_3$ at 500 and 125 MHz, respectively.

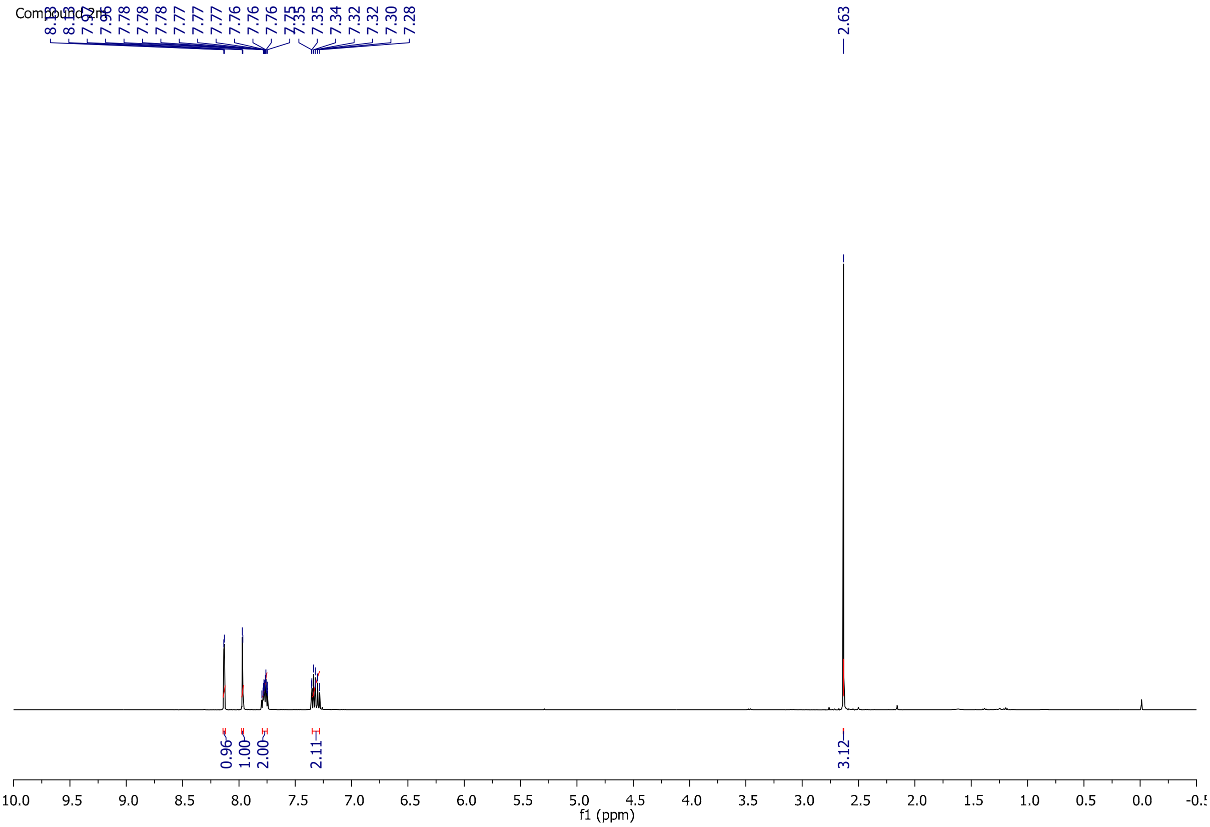

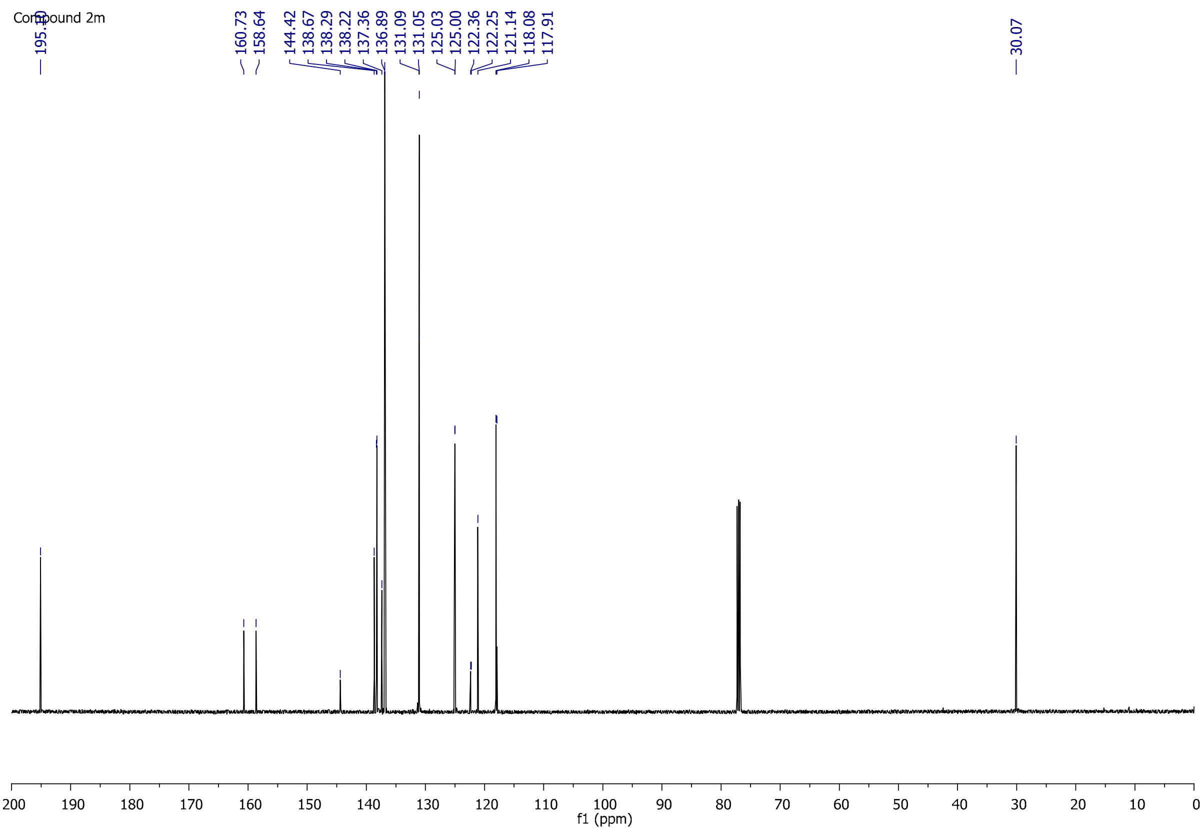

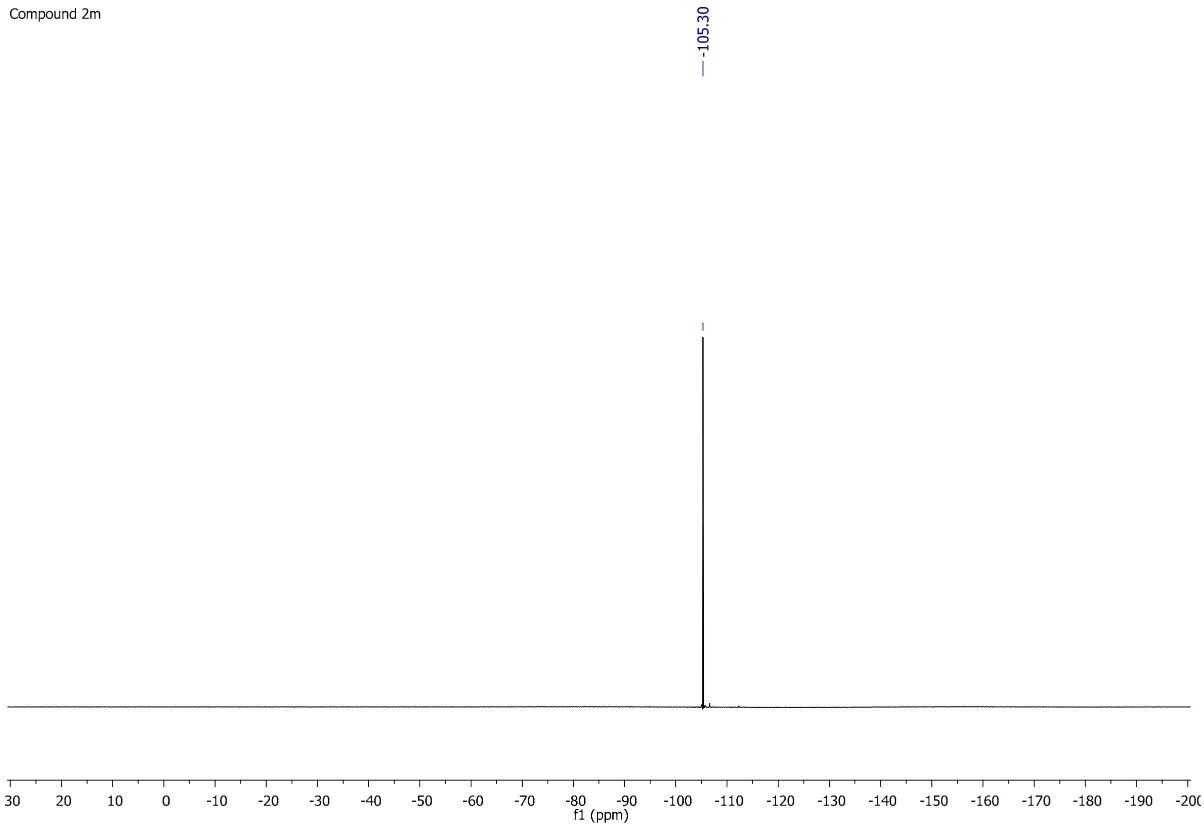

Figure S1.13: ^{1}H-, ^{13}C- and ^{19}F NMR of **2m** in $CDCl_3$ at 500, 125 and 470 MHz, respectively.

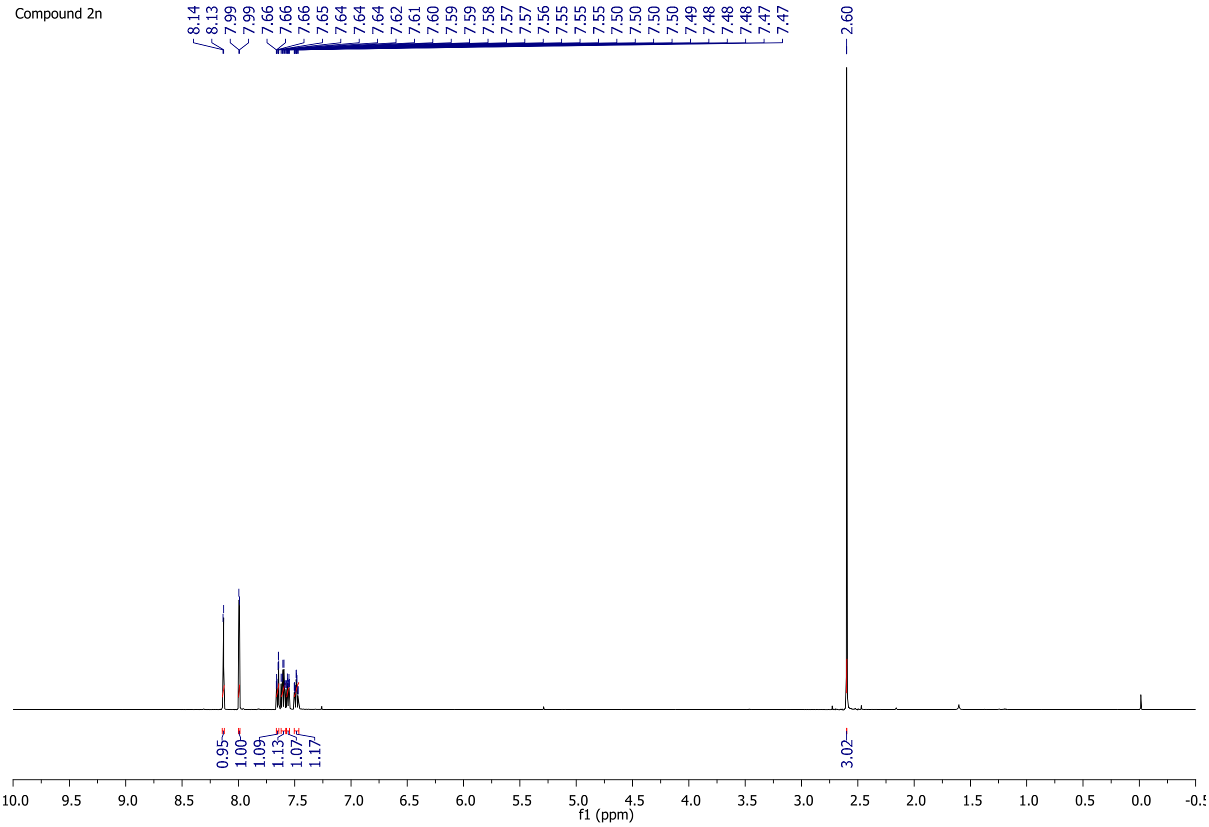
Compound 2n
8.14
8.13
7.99
7.99
7.66
7.66
7.66
7.65
7.64
7.64
7.64
7.62
7.61
7.60
7.59
7.59
7.58
7.57
7.57
7.56
7.55
7.55
7.55
7.50
7.50
7.50
7.50
7.49
7.48
7.48
7.47
7.47
2.60
0.95
1.00
1.09
1.13
1.07
1.17
3.02
10.0 9.5 9.0 8.5 8.0 7.5 7.0 6.5 6.0 5.5 5.0 4.5 4.0 3.5 3.0 2.5 2.0 1.5 1.0 0.5 0.0
f1 (ppm)

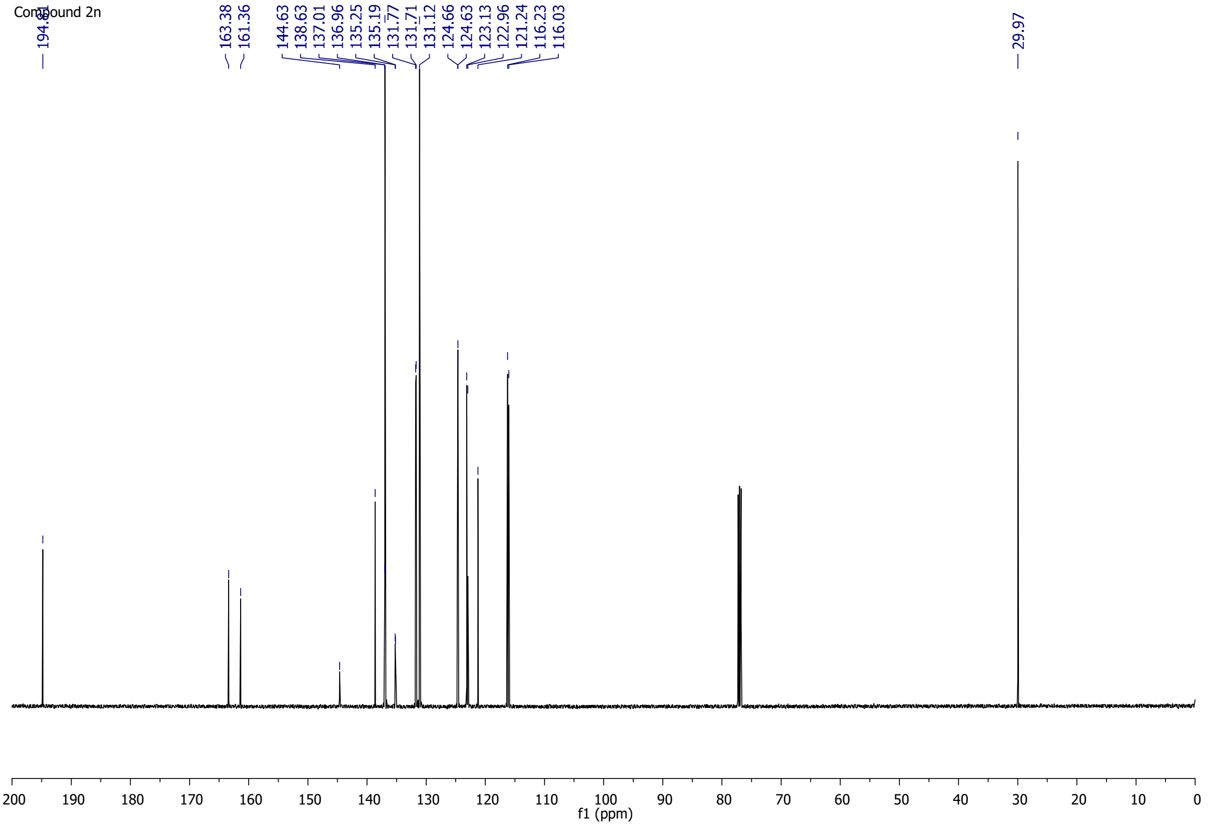
Compound 2n
163.38
161.36
144.63
138.63
137.01
136.96
135.25
135.19
131.77
131.71
131.12
124.66
124.63
123.13
122.96
121.24
116.23
116.03
29.97
200 190 180 170 160 150 140 130 120 110 100 90 80 70 60 50 40 30 20 10 0
f1 (ppm)

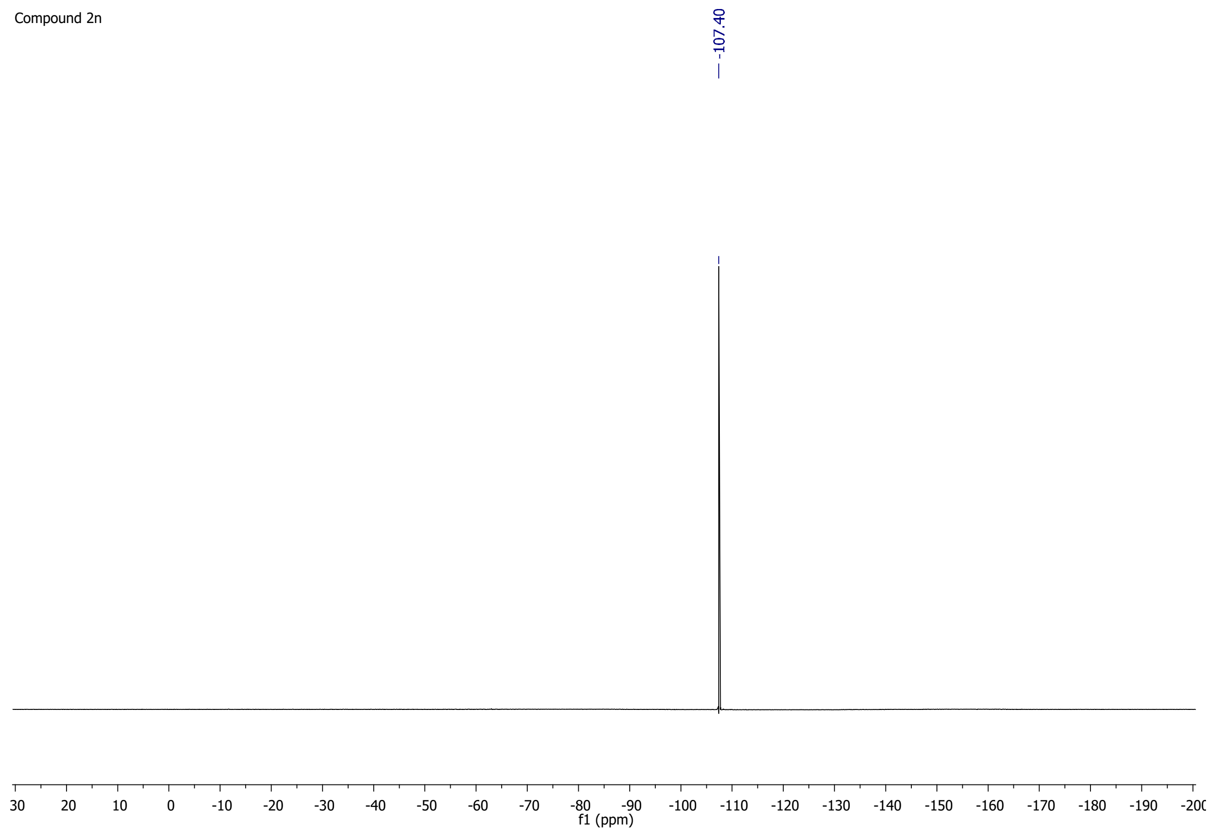

Figure S1.14: ^{1}H-, ^{13}C- and ^{19}F NMR of **2n** in $CDCl_3$ at 500, 125 and 470 MHz, respectively.

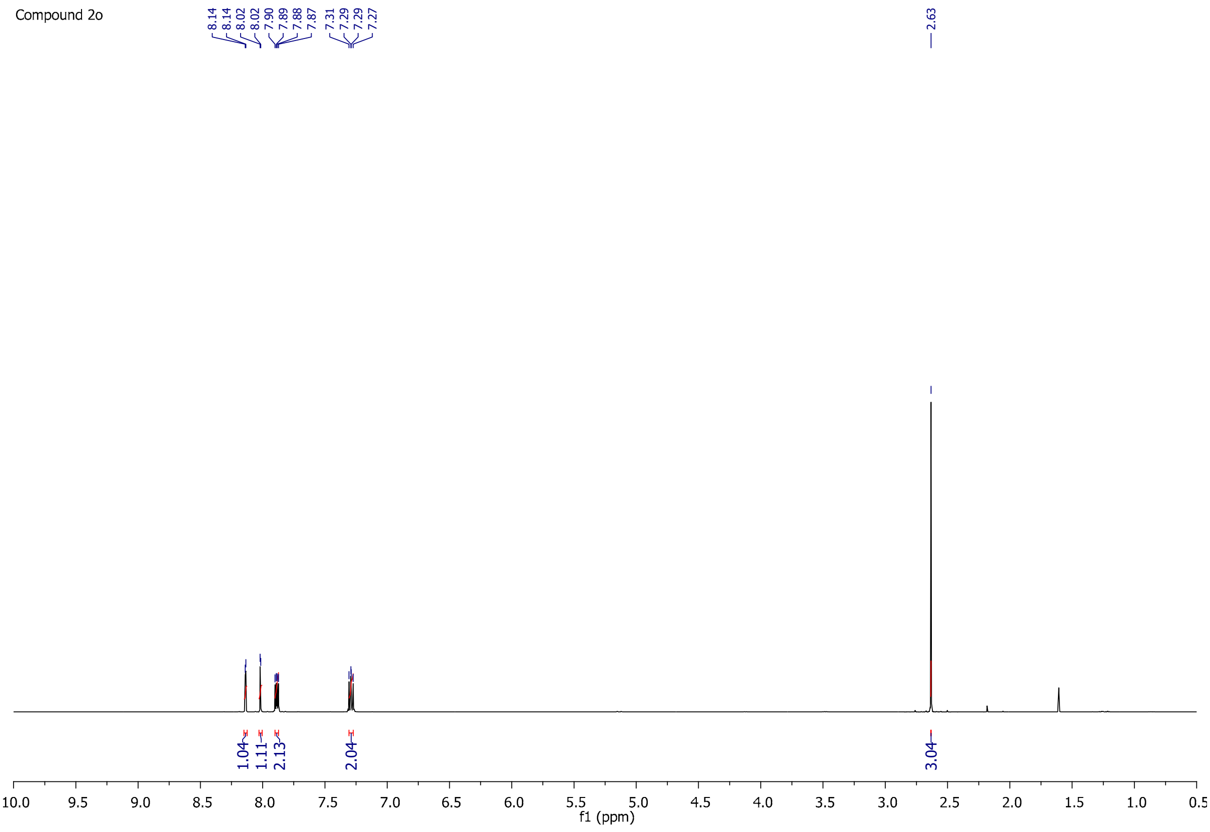

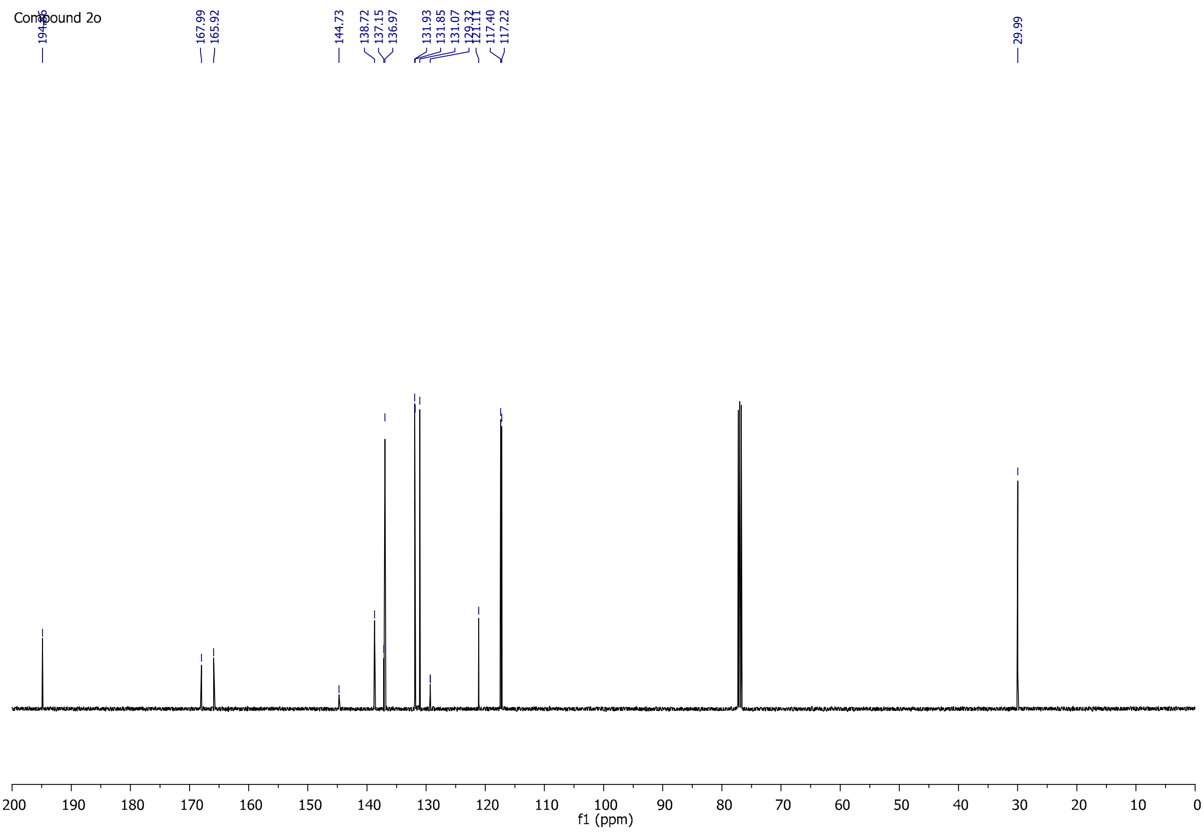

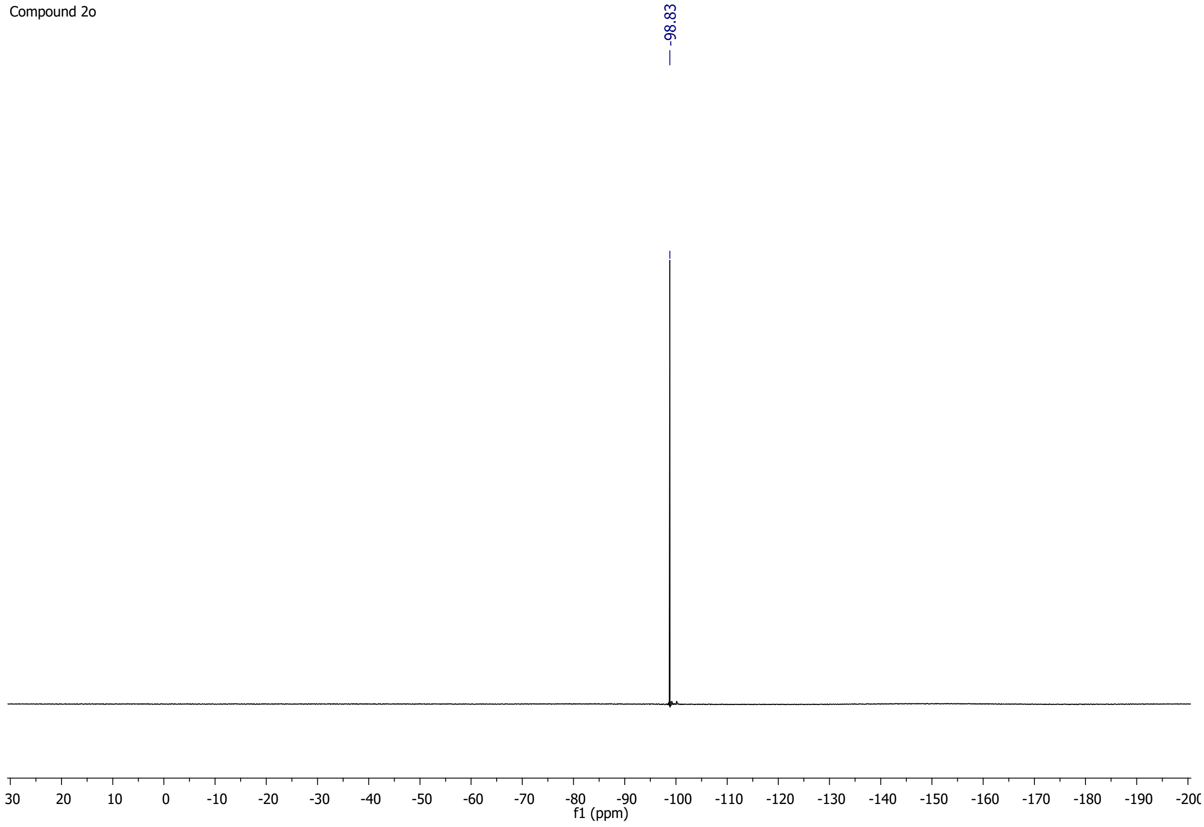

Figure S1.15: ^{1}H-, ^{13}C- and ^{19}F NMR of **2o** in $CDCl_3$ at 500, 125 and 470 MHz, respectively.

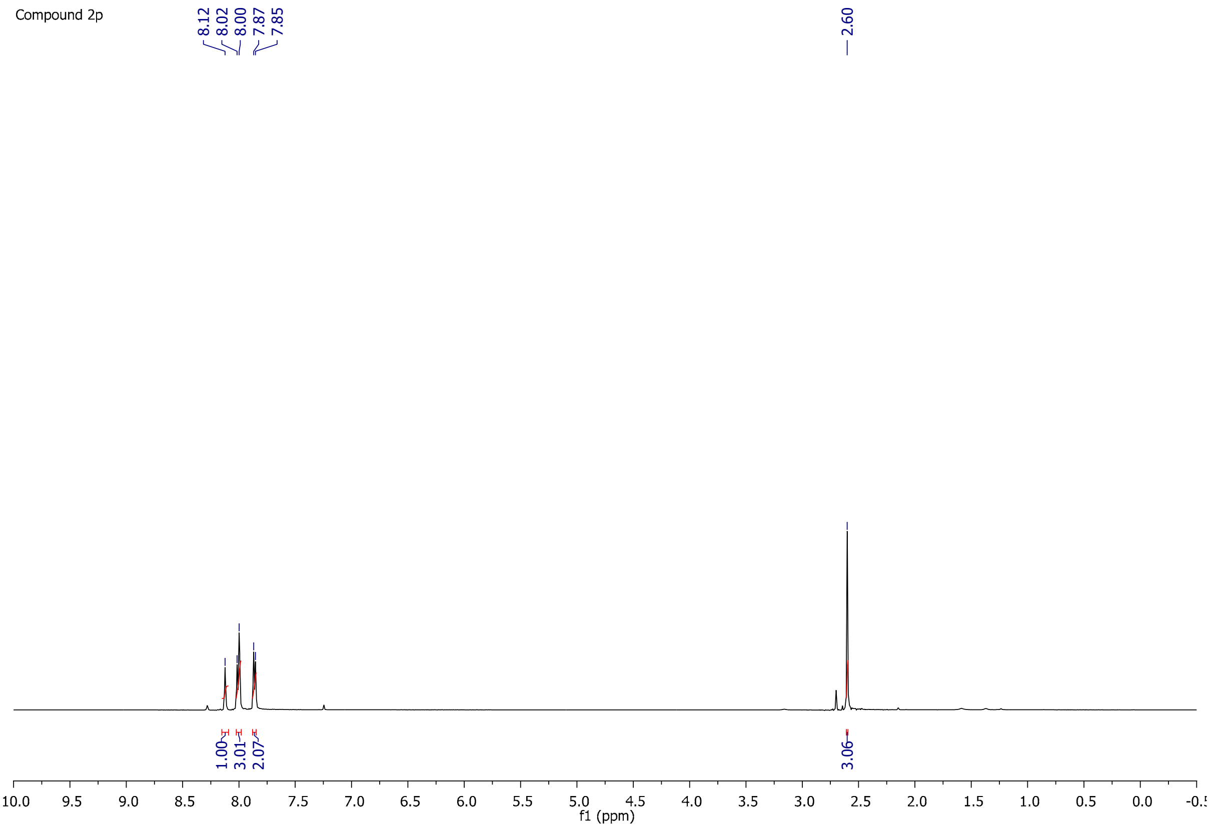
Compound 2p
8.12
8.02
8.00
7.87
7.85
2.60
1.00
3.01
2.07
3.06
f1 (ppm)

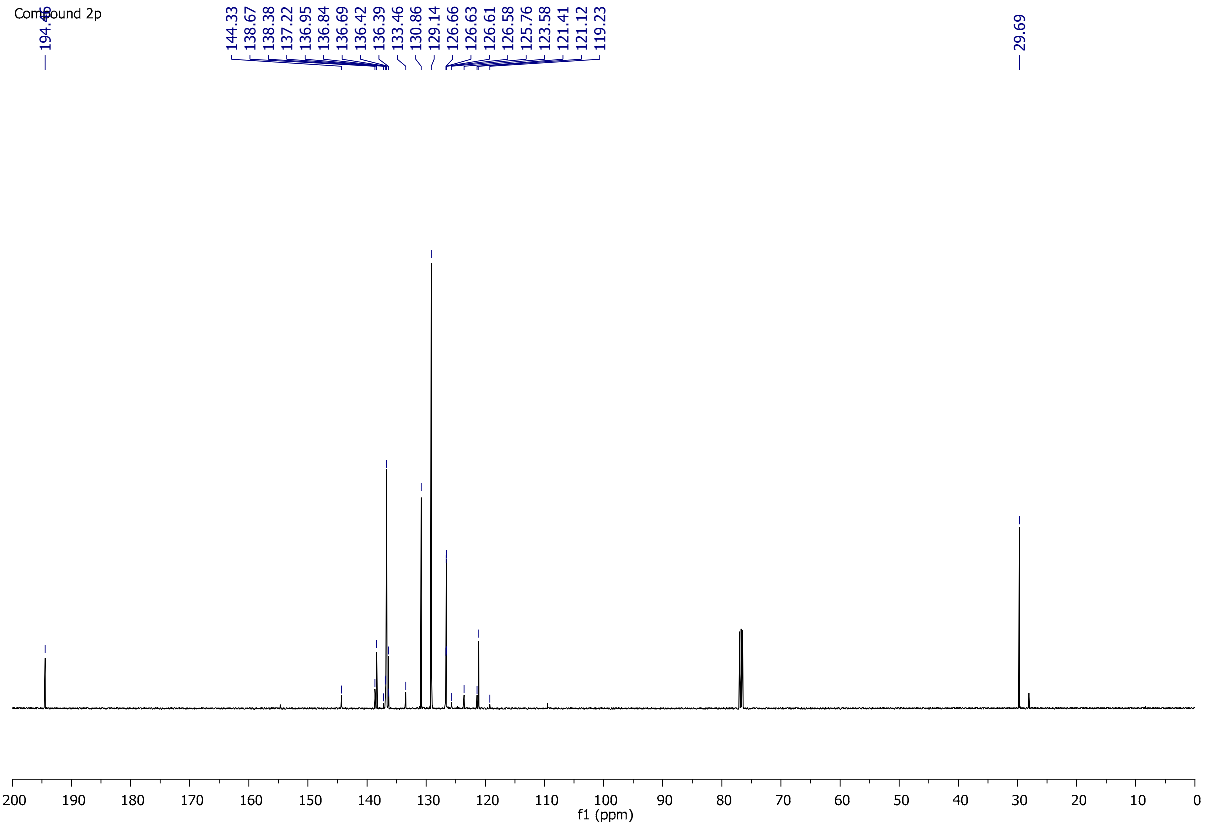
Compound 2p
194.40
144.33
138.67
138.38
137.22
136.95
136.84
136.69
136.42
136.39
133.46
130.86
129.14
126.66
126.63
126.61
126.58
125.76
123.58
121.41
121.12
119.23
29.69
f1 (ppm)

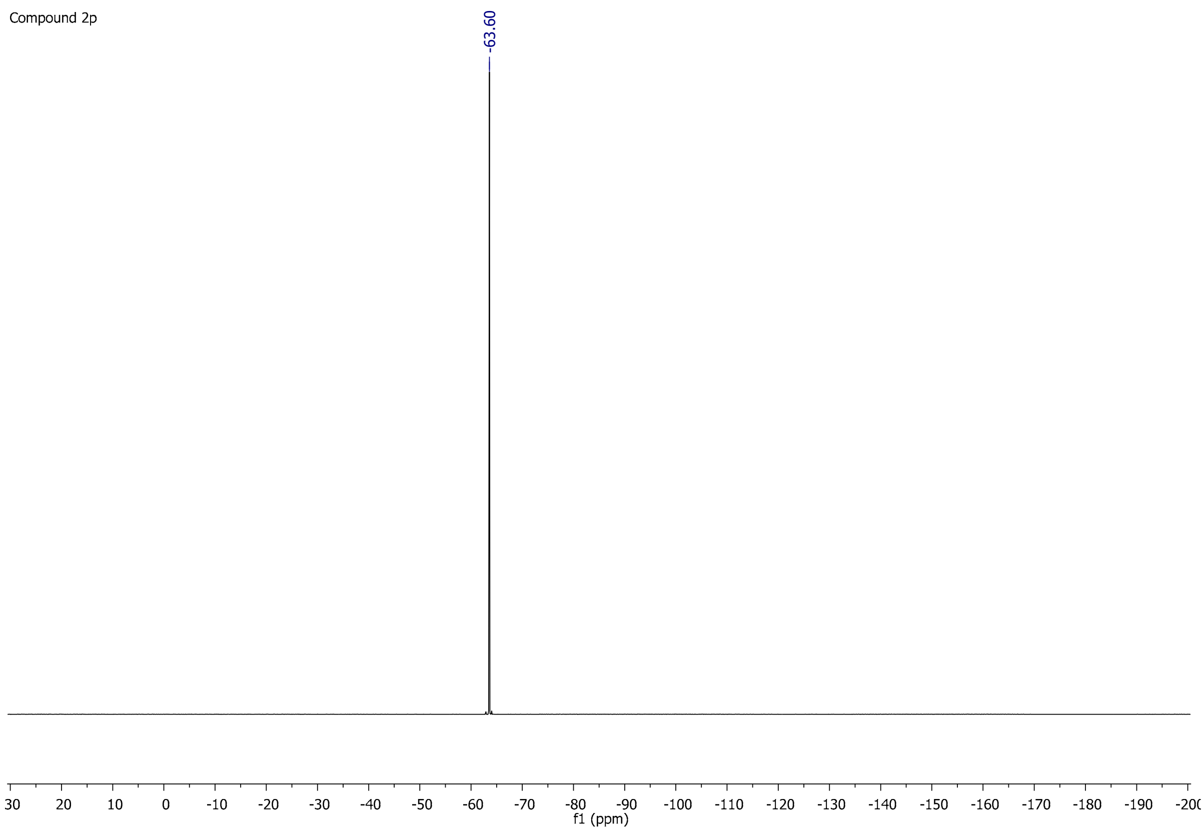

Figure S1.16: ^{1}H-, ^{13}C- and ^{19}F NMR of **2p** in $CDCl_3$ at 500, 125 and 470 MHz, respectively.

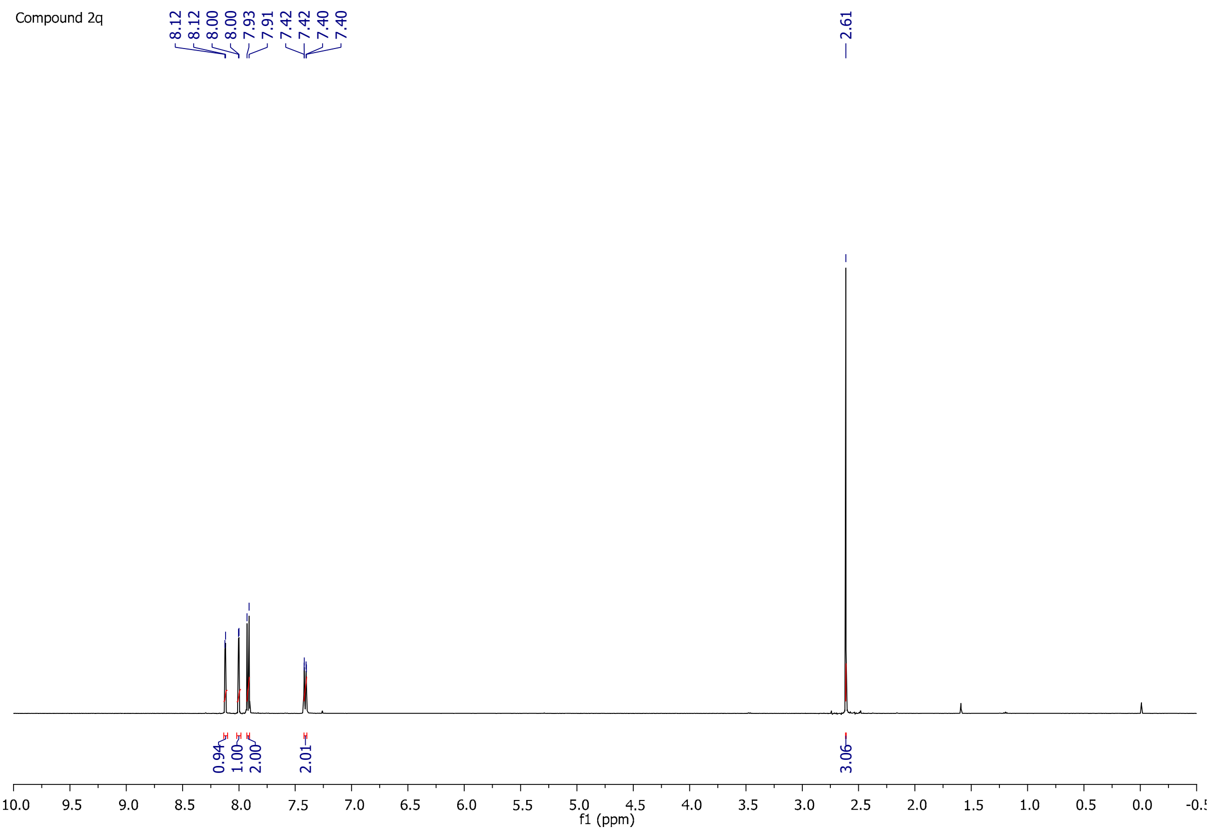
Compound 2q
8.12
8.12
8.00
8.00
7.93
7.91
7.42
7.42
7.40
7.40
2.61
0.94
1.00
2.00
2.01
3.06
f1 (ppm)

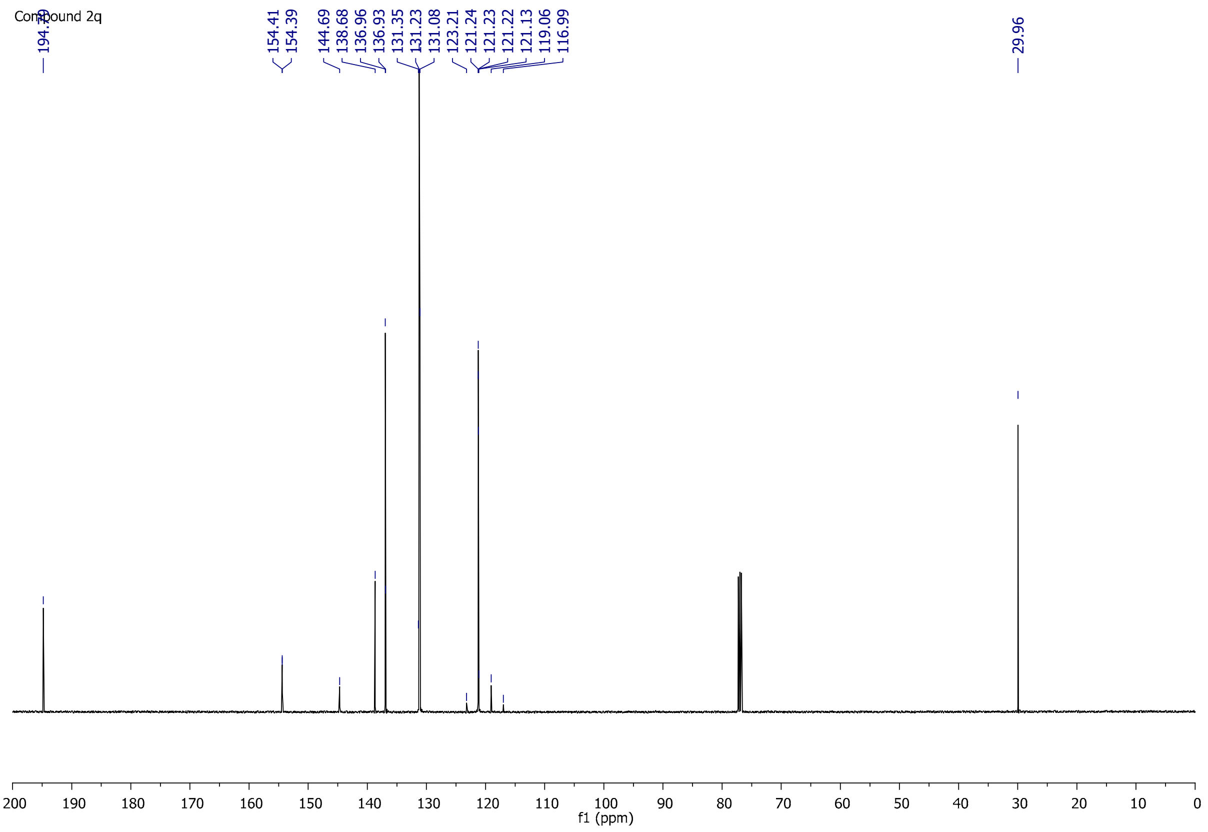
Compound 2q
154.41
154.39
144.69
138.68
136.96
136.93
131.35
131.23
131.08
123.21
121.24
121.23
121.22
121.13
119.06
116.99
29.96
f1 (ppm)

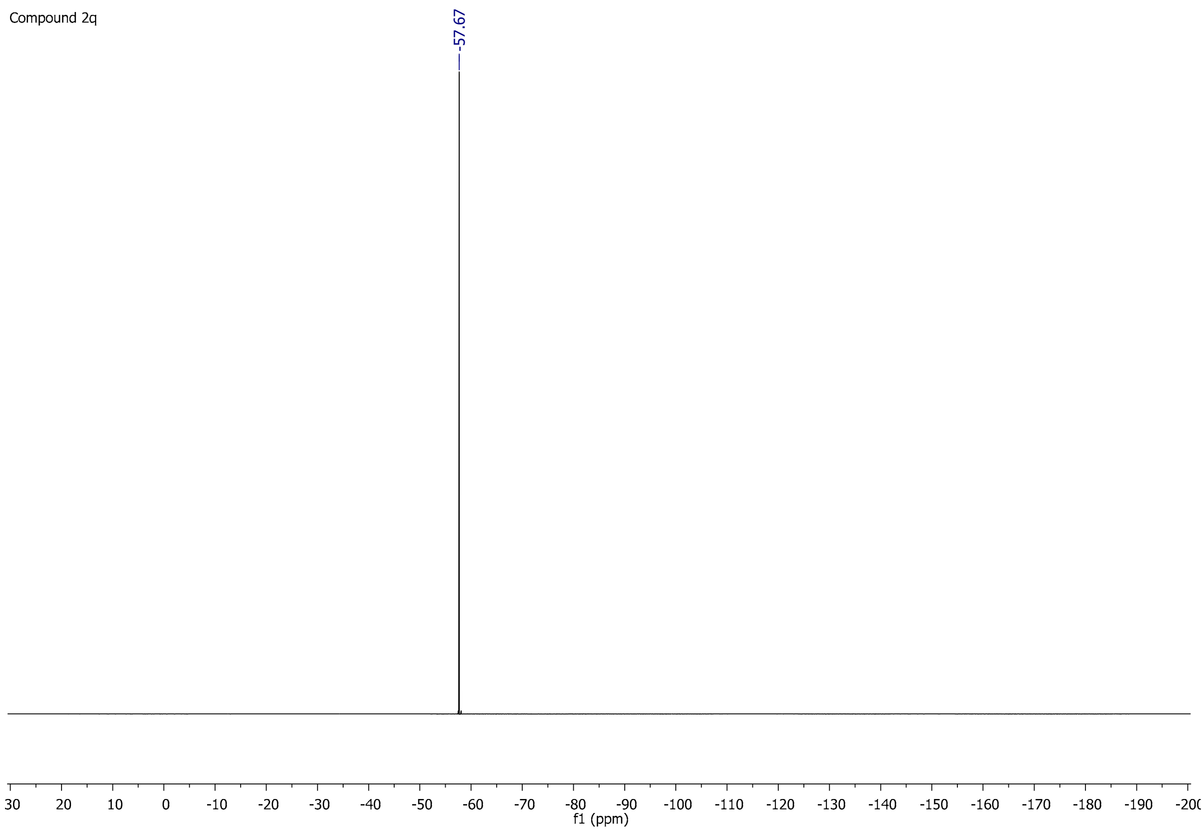

Figure S1.17: ^{1}H-, ^{13}C- and ^{19}F NMR of **2q** in $CDCl_3$ at 500, 125 and 470 MHz, respectively.

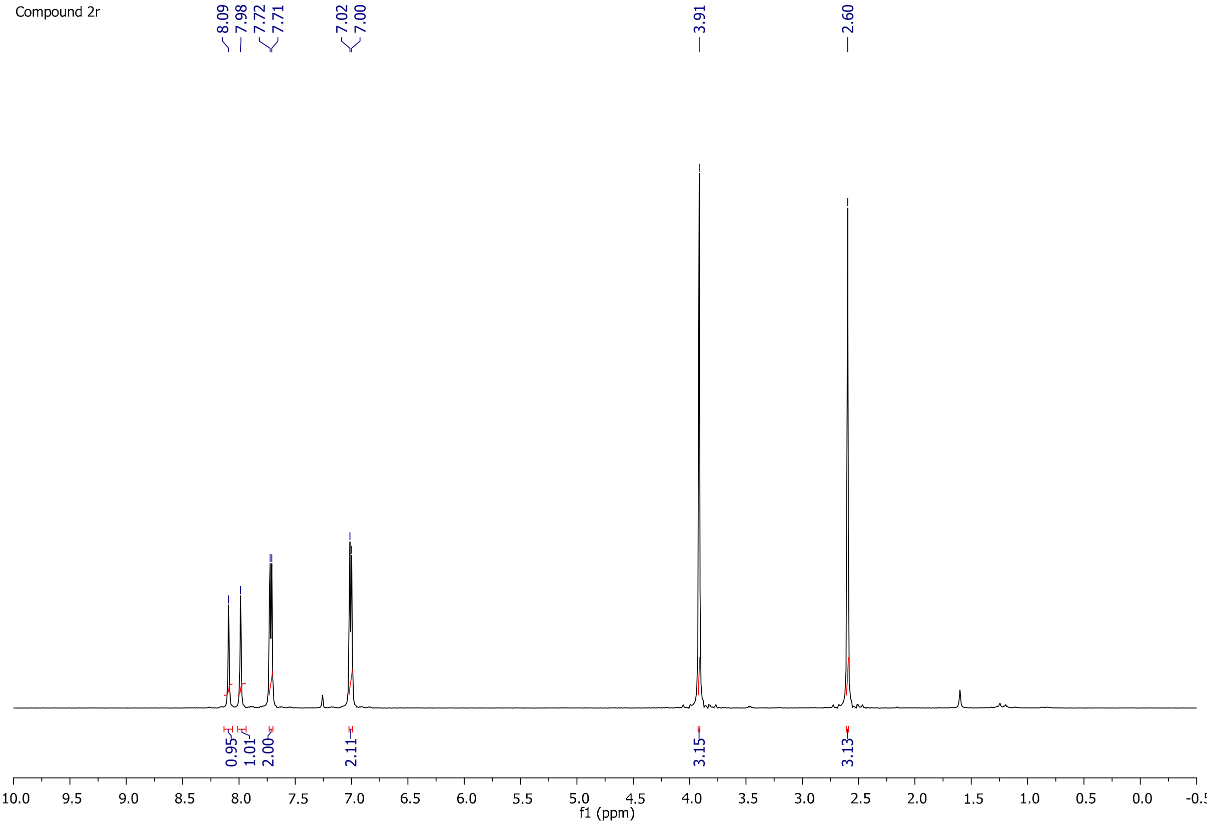

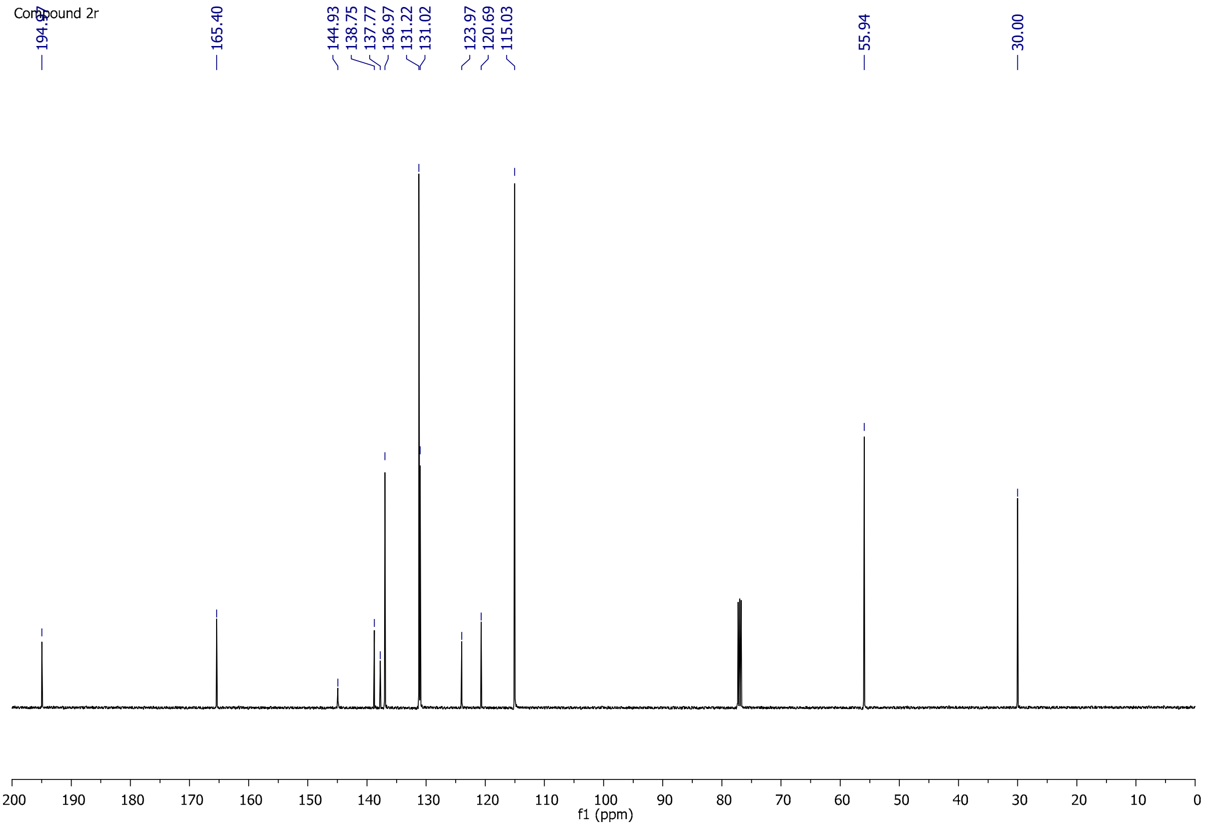

Figure S1.18: ^{1}H- and ^{13}C-NMR of **2r** in $CDCl_3$ at 500 and 125 MHz, respectively.

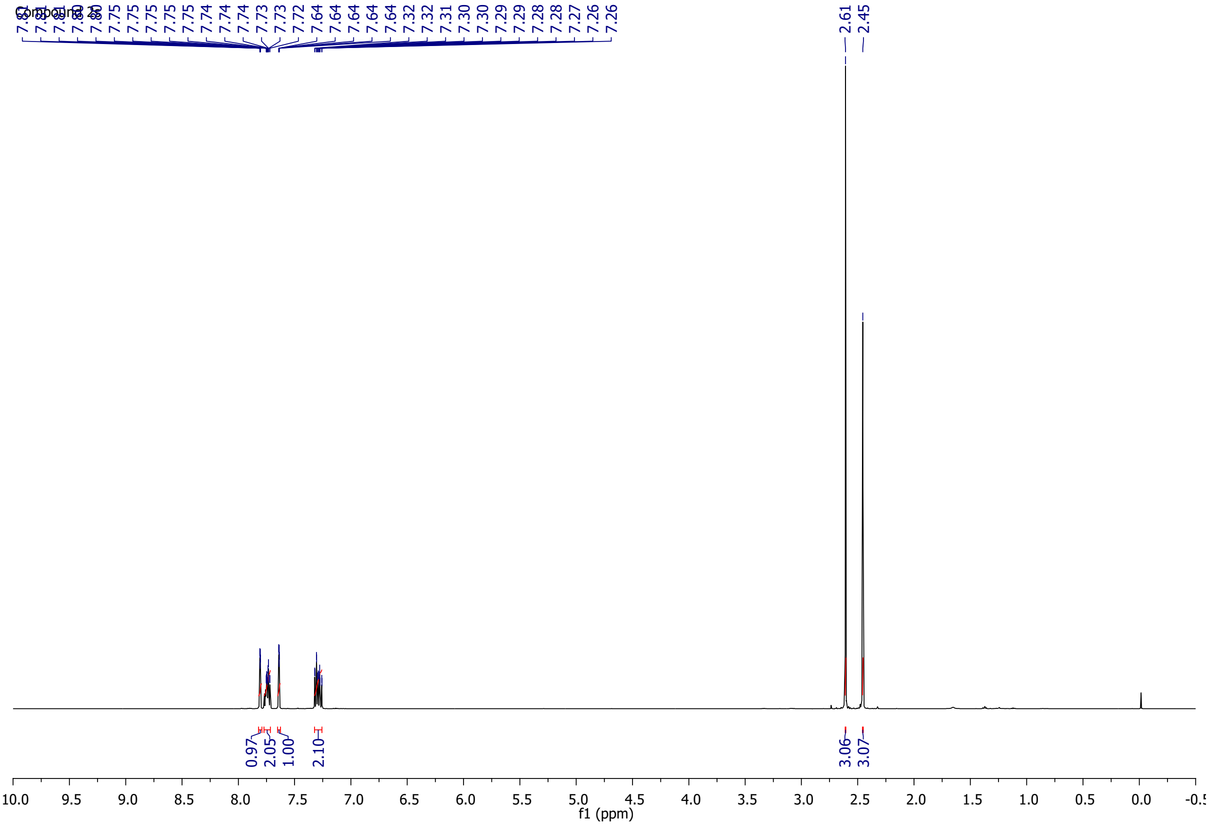

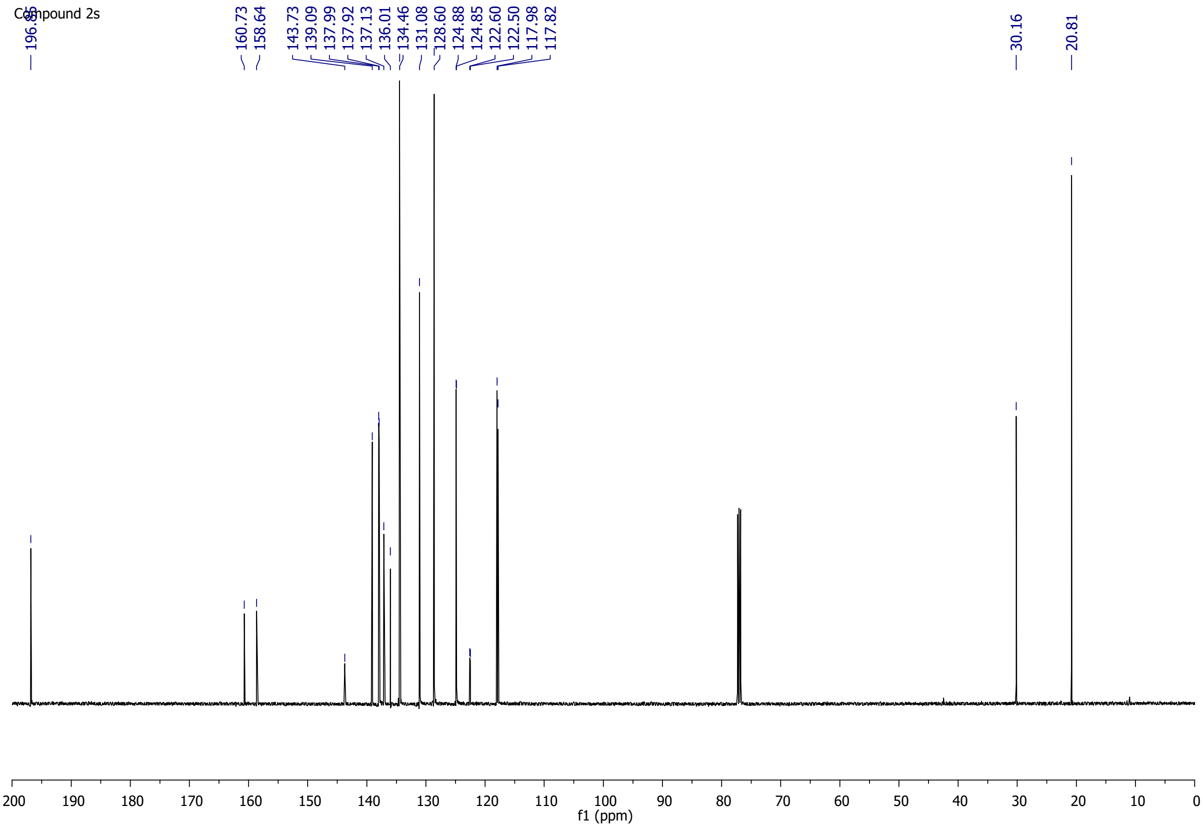

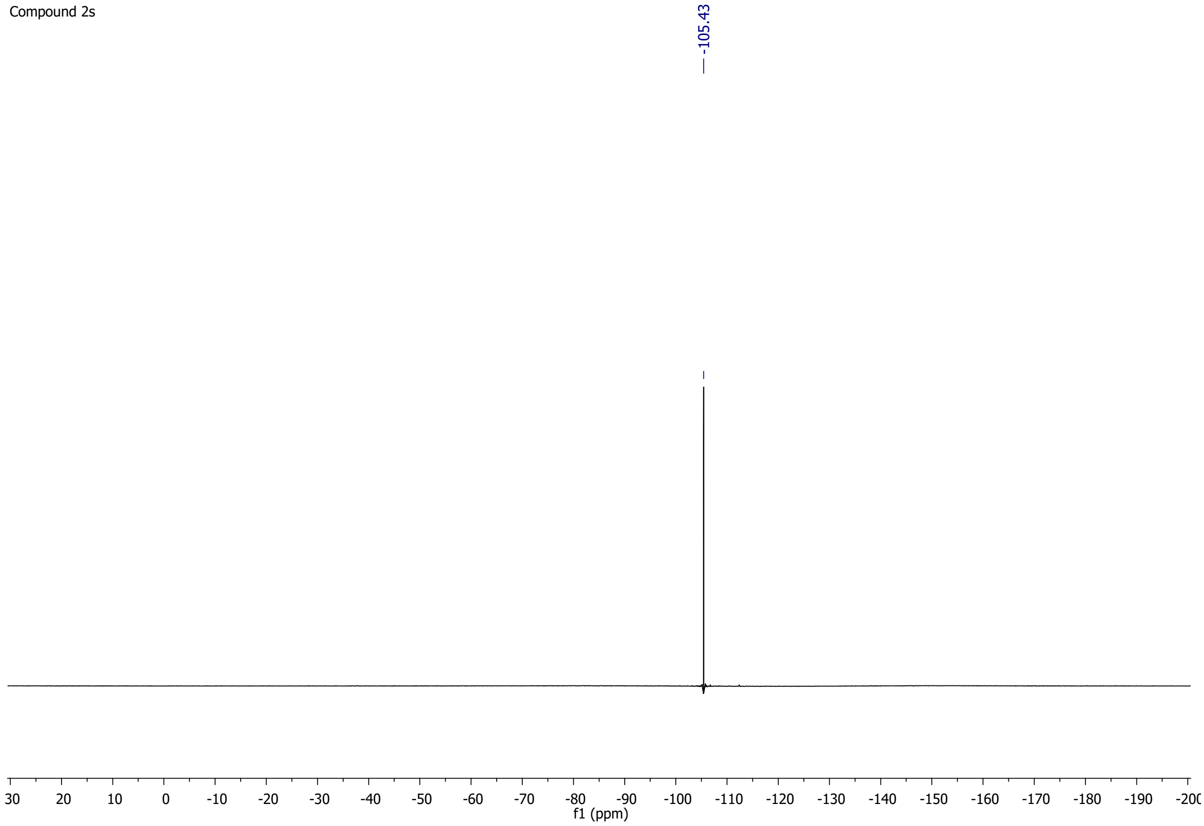

Figure S1.19: ^{1}H-, ^{13}C- and ^{19}F NMR of **2s** in $CDCl_3$ at 500, 125 and 470 MHz, respectively.

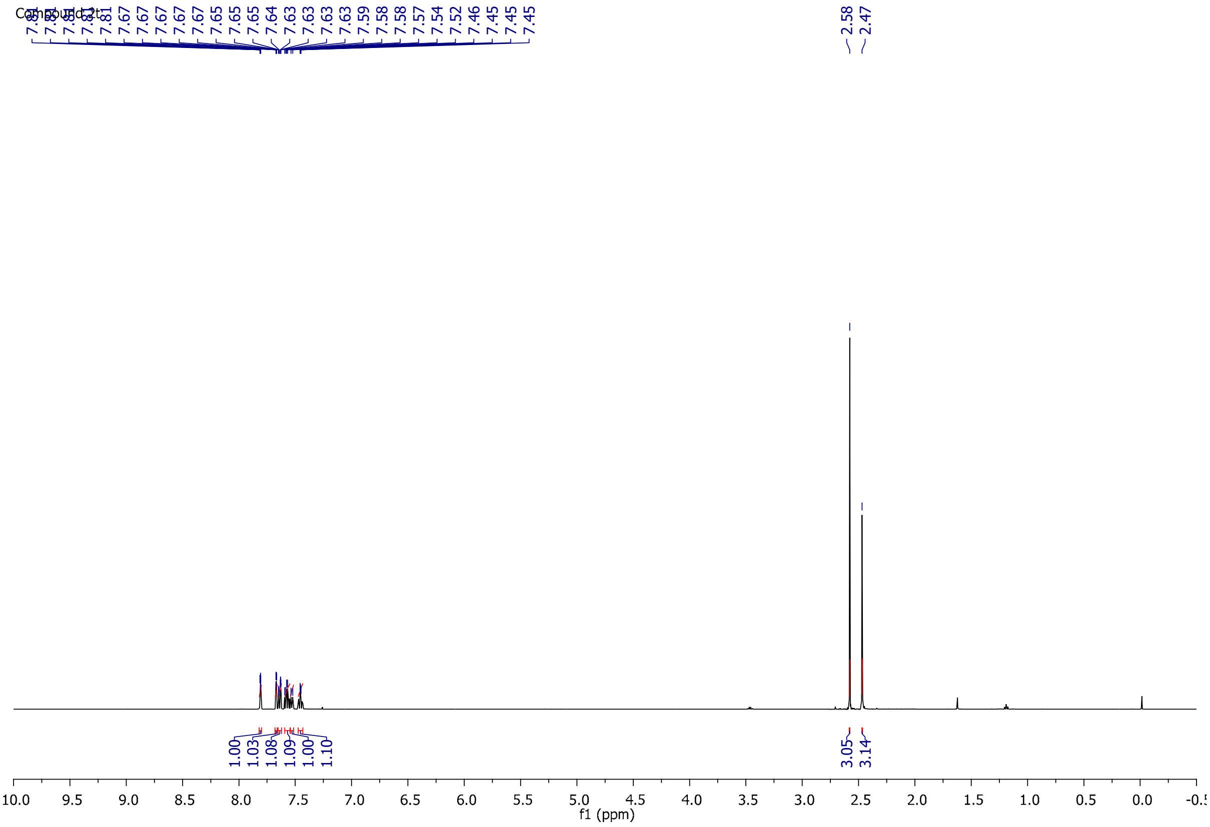
Compound 2t
7.81
7.67 7.67 7.67 7.67 7.65 7.65 7.64 7.63 7.63 7.63 7.63 7.59 7.58 7.58 7.57 7.54 7.52 7.46 7.45 7.45
2.58 2.47
1.00 1.03 1.08 1.09 1.00 1.10
3.05 3.14
10.0 9.5 9.0 8.5 8.0 7.5 7.0 6.5 6.0 5.5 5.0 4.5 4.0 3.5 3.0 2.5 2.0 1.5 1.0 0.5 0.0
f1 (ppm)

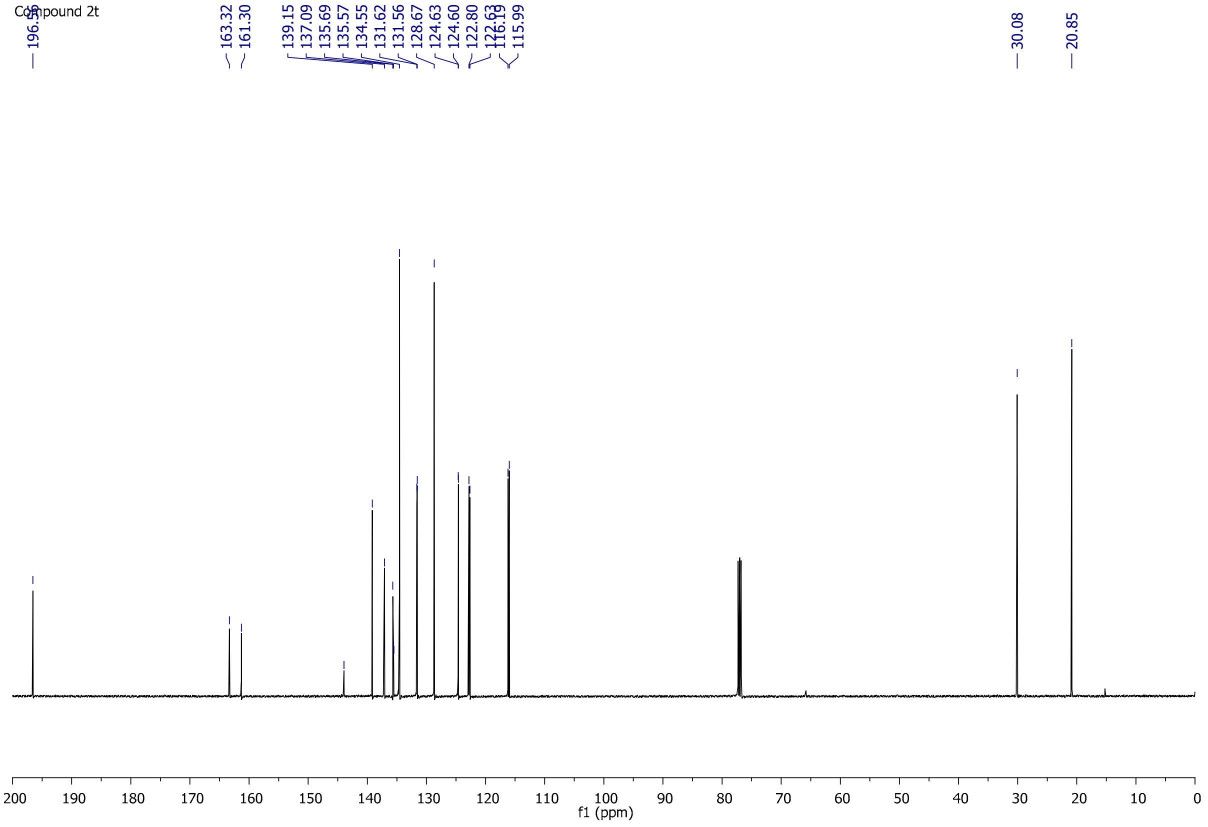
Compound 2t
163.32 161.30
139.15 137.09 135.69 135.57 134.55 131.62 131.56 128.67 124.63 124.60 122.80 122.63 116.19 115.99
30.08 20.85
200 190 180 170 160 150 140 130 120 110 100 90 80 70 60 50 40 30 20 10 0
f1 (ppm)

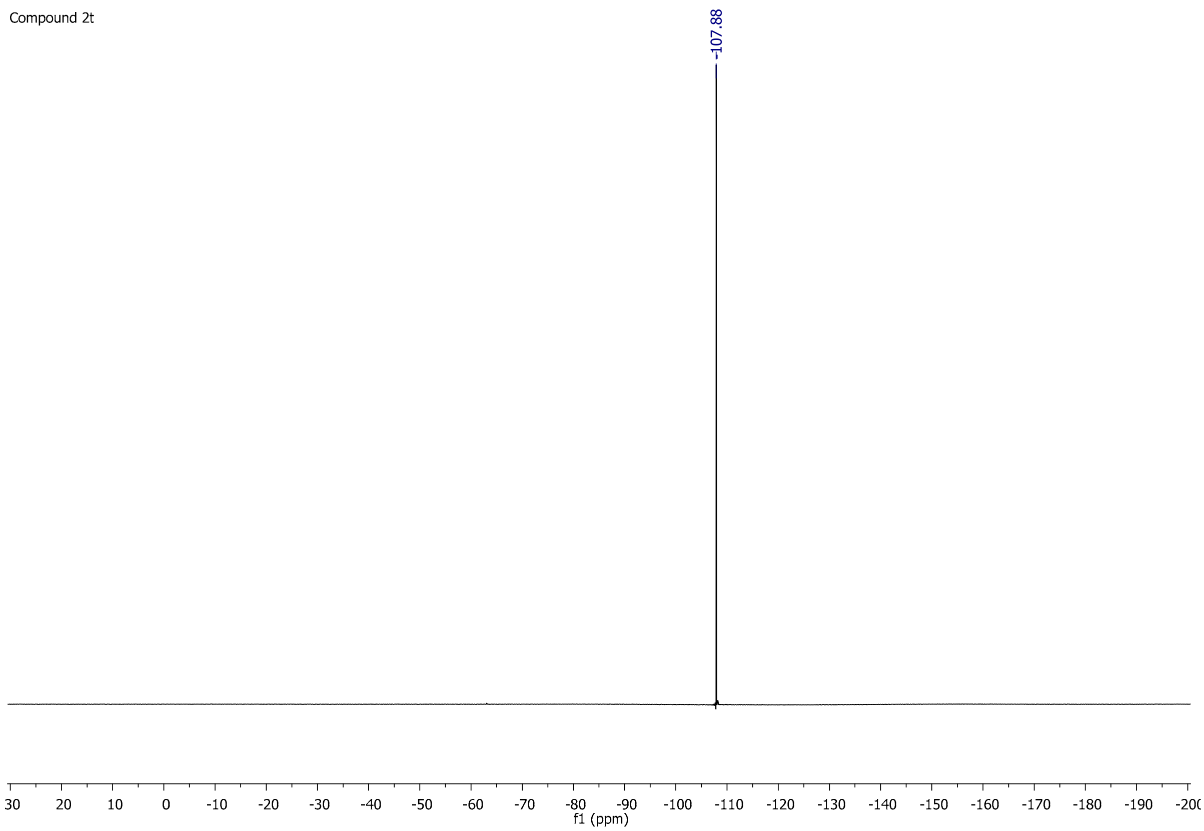

Figure S1.20: ^{1}H-, ^{13}C- and ^{19}F NMR of **2t** in $CDCl_3$ at 500, 125 and 470 MHz, respectively.

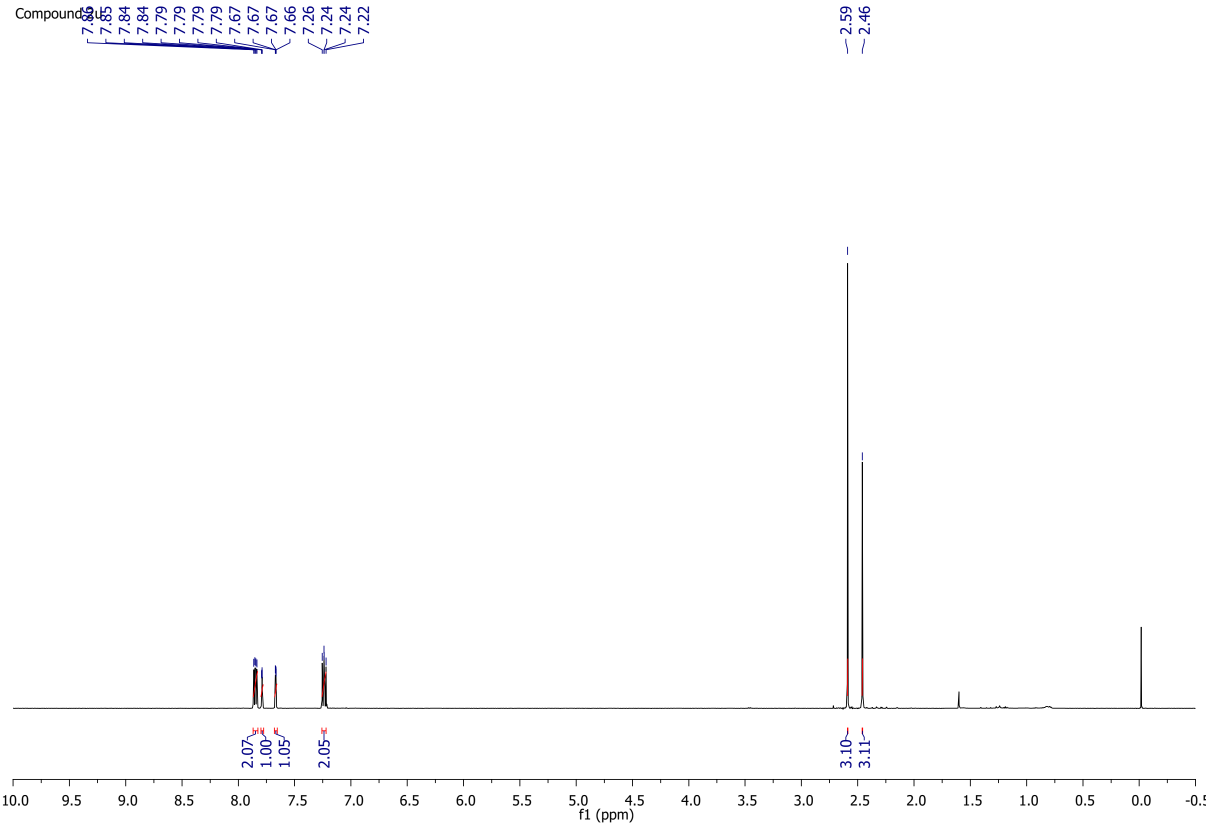
Compound 2u
7.86
7.85
7.84
7.84
7.79
7.79
7.79
7.79
7.67
7.67
7.67
7.66
7.26
7.24
7.24
7.22
2.59
2.46
2.07
1.00
1.05
2.05
3.10
3.11
f1 (ppm)

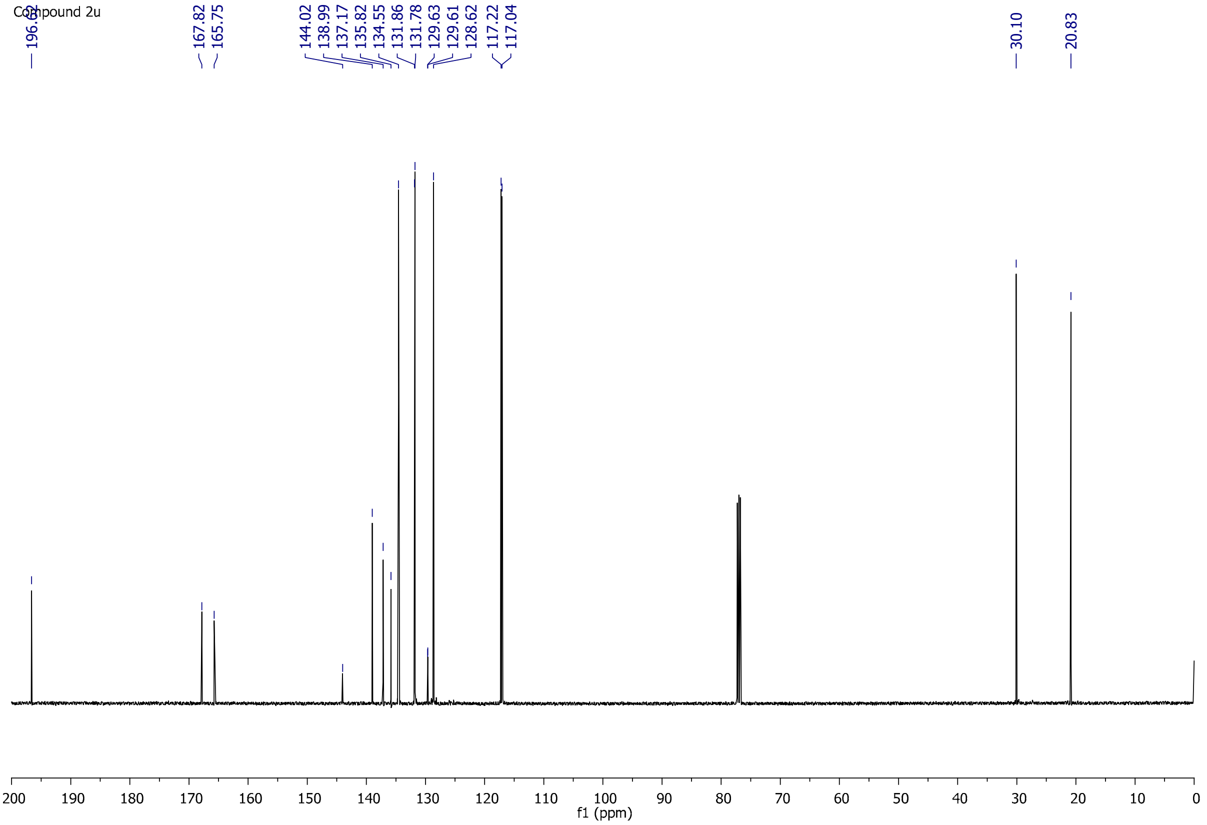
Compound 2u
196.62
167.82
165.75
144.02
138.99
137.17
135.82
134.55
131.86
131.78
129.63
129.61
128.62
117.22
117.04
30.10
20.83
f1 (ppm)

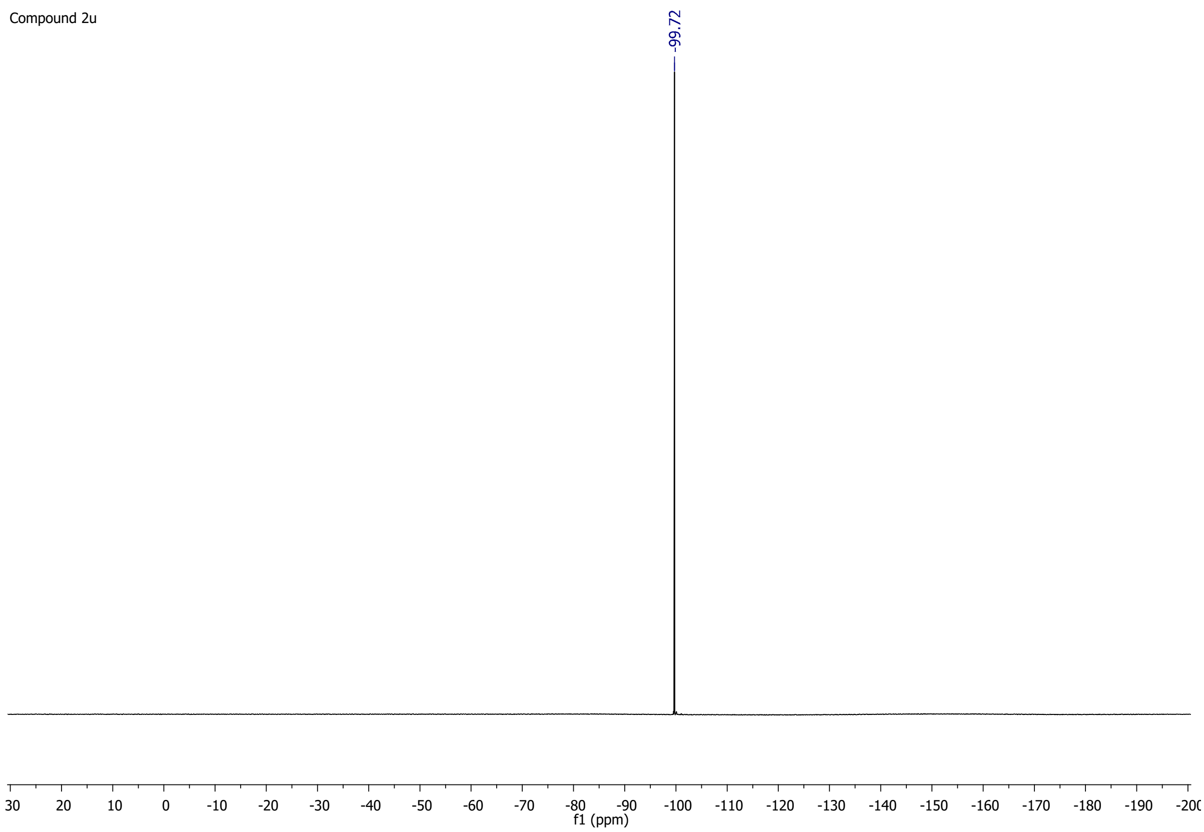

Figure S1.21: ^{1}H-, ^{13}C- and ^{19}F NMR of **2u** in $CDCl_3$ at 500, 125 and 470 MHz, respectively.

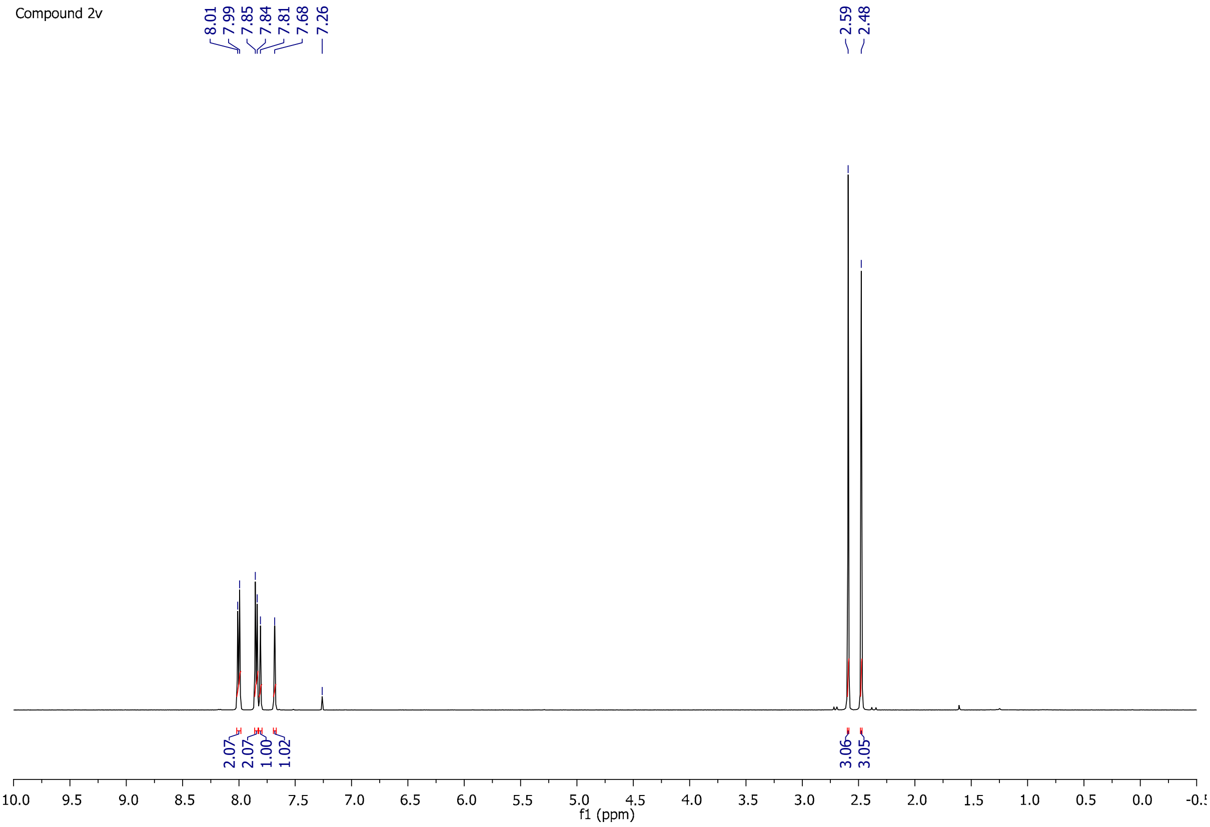

Compound 2v
8.01
7.99
7.85
7.84
7.81
7.68
7.26
2.59
2.48
2.07
2.07
1.00
1.02
3.06
3.05
f1 (ppm)

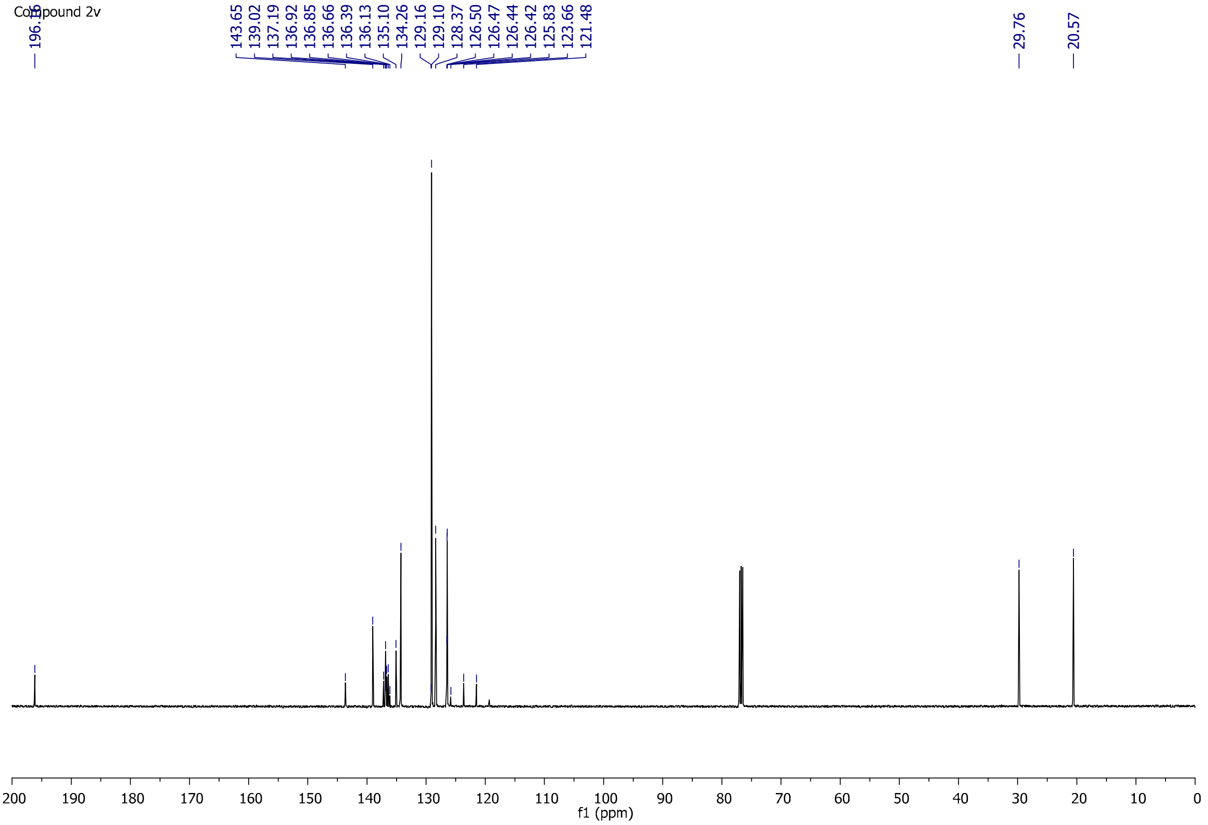

Compound 2v
196.16
143.65
139.02
137.19
136.92
136.85
136.66
136.39
136.13
135.10
134.26
129.16
129.10
128.37
126.50
126.47
126.44
126.42
125.83
123.66
121.48
29.76
20.57
f1 (ppm)

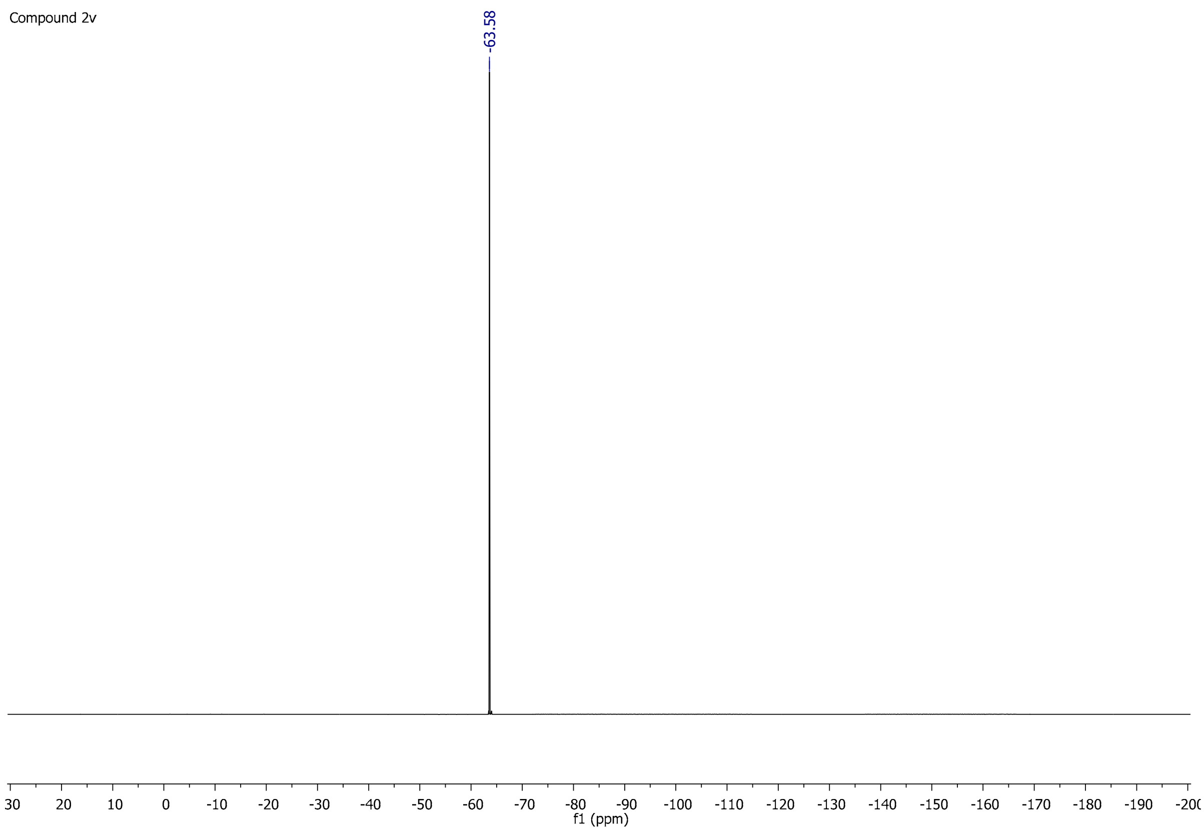

Figure S1.22: ^{1}H-, ^{13}C- and ^{19}F NMR of **2v** in $CDCl_3$ at 500, 125 and 470 MHz, respectively.

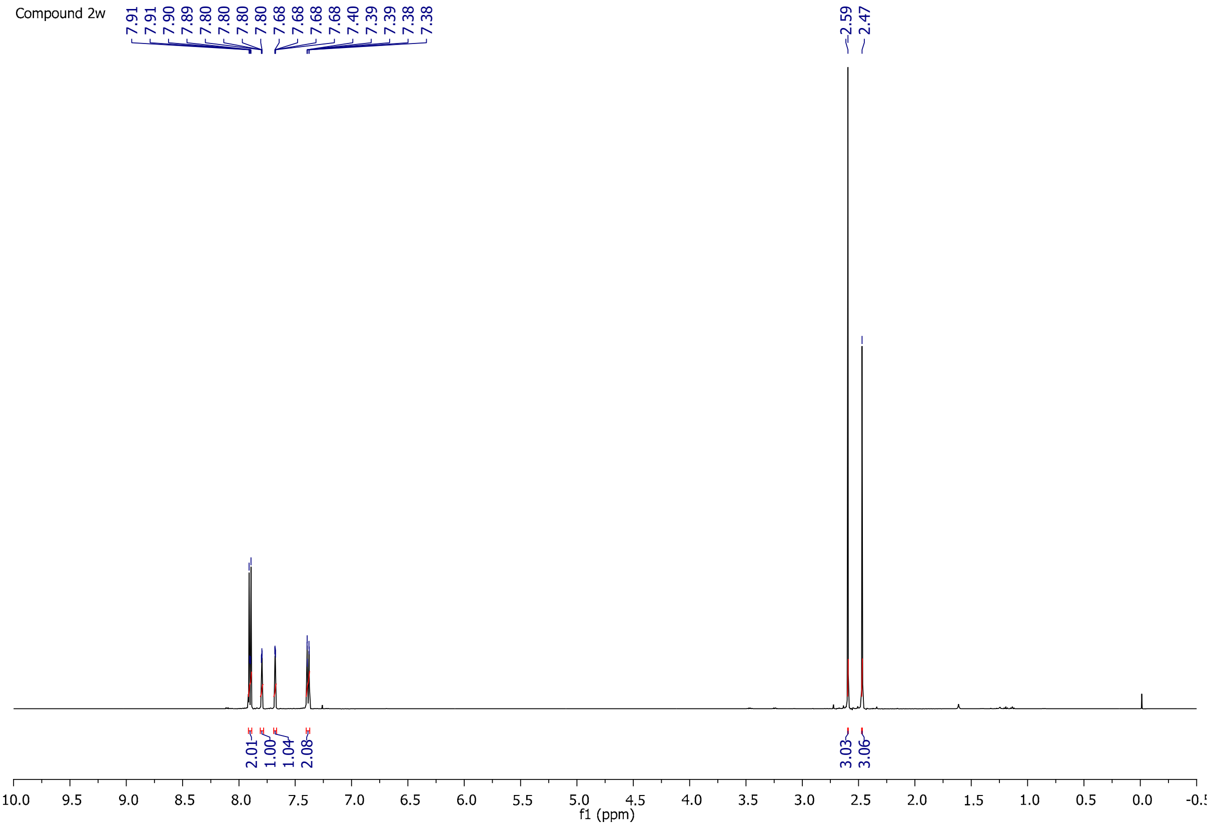

Compound 2w
7.91
7.91
7.90
7.89
7.80
7.80
7.80
7.80
7.68
7.68
7.68
7.68
7.68
7.40
7.39
7.39
7.38
7.38
2.59
2.47
2.01
1.00
1.04
2.08
3.03
3.06
f1 (ppm)

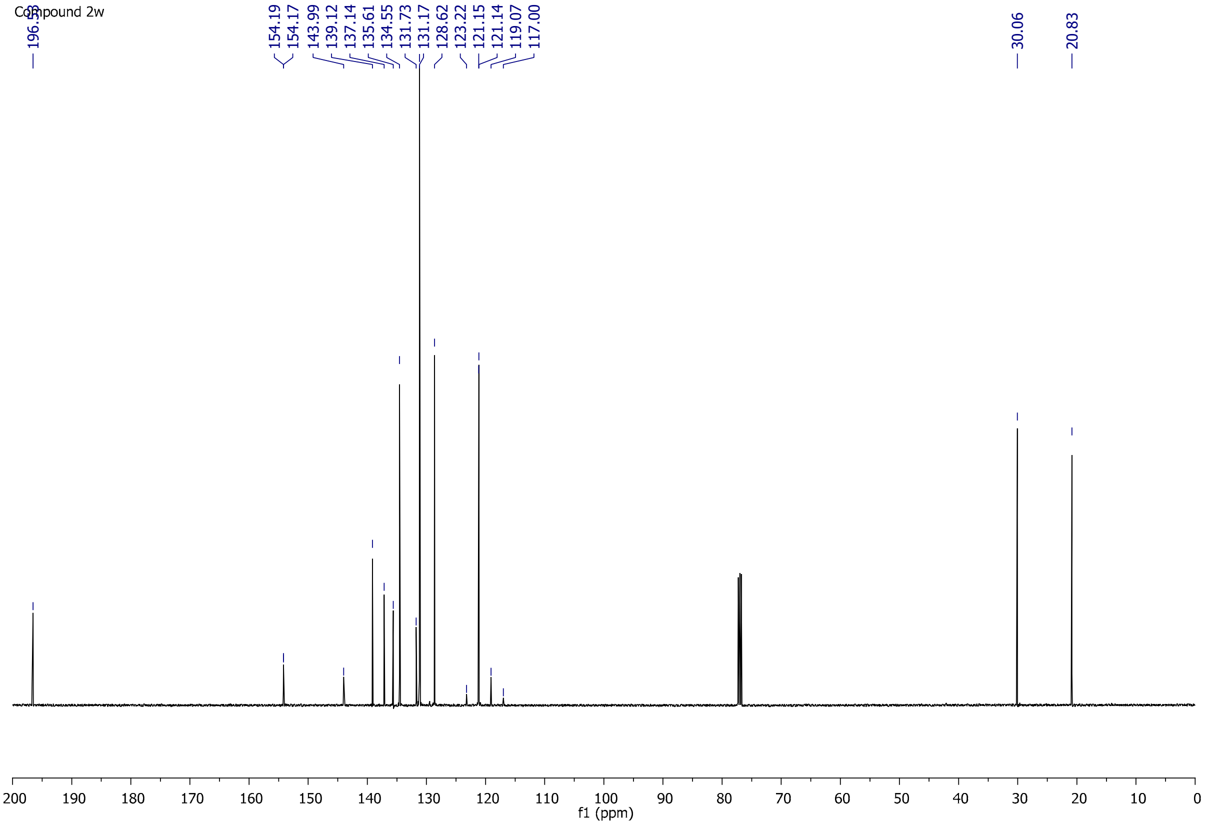

Compound 2w
196.59
154.19
154.17
143.99
139.12
137.14
135.61
134.55
131.73
131.17
128.62
123.22
121.15
121.14
119.07
117.00
30.06
20.83
f1 (ppm)

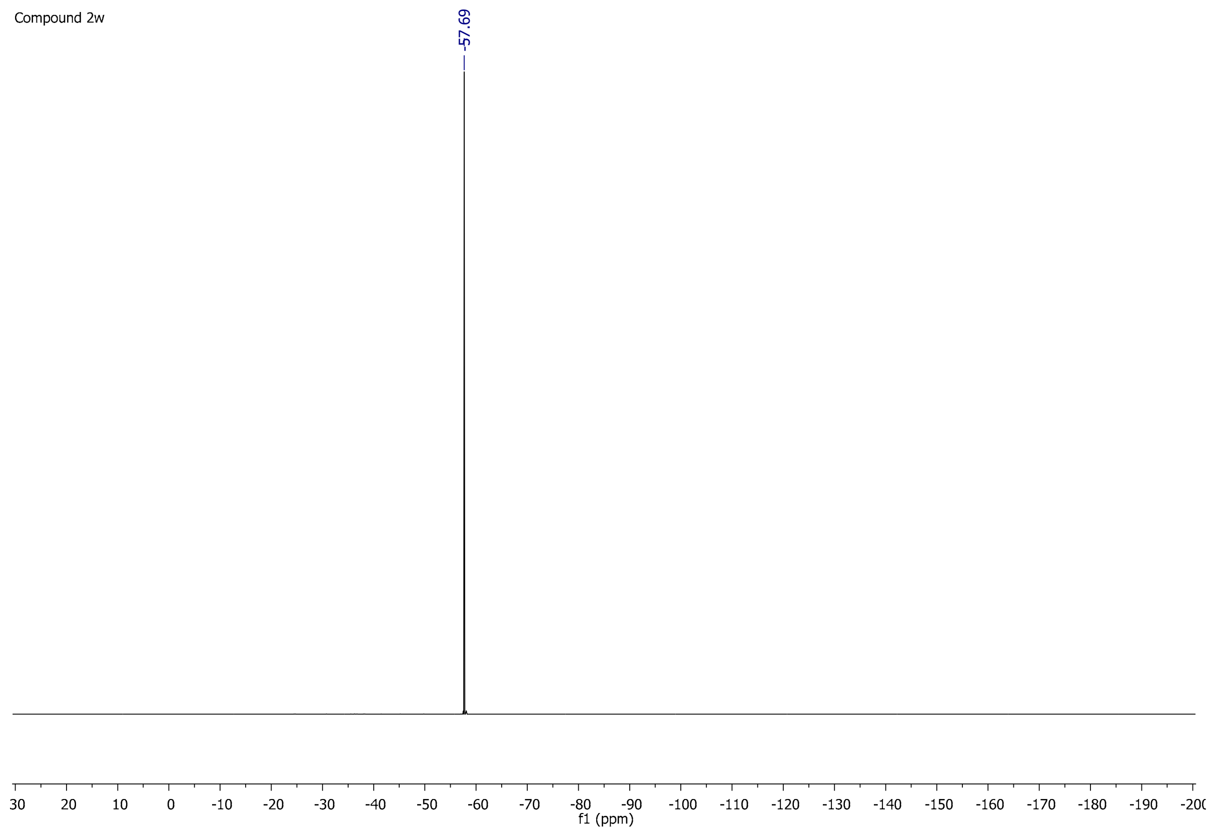

Figure S1.23: ^1H-, ^{13}C- and ^{19}F NMR of **2w** in $CDCl_3$ at 500, 125 and 470 MHz, respectively.

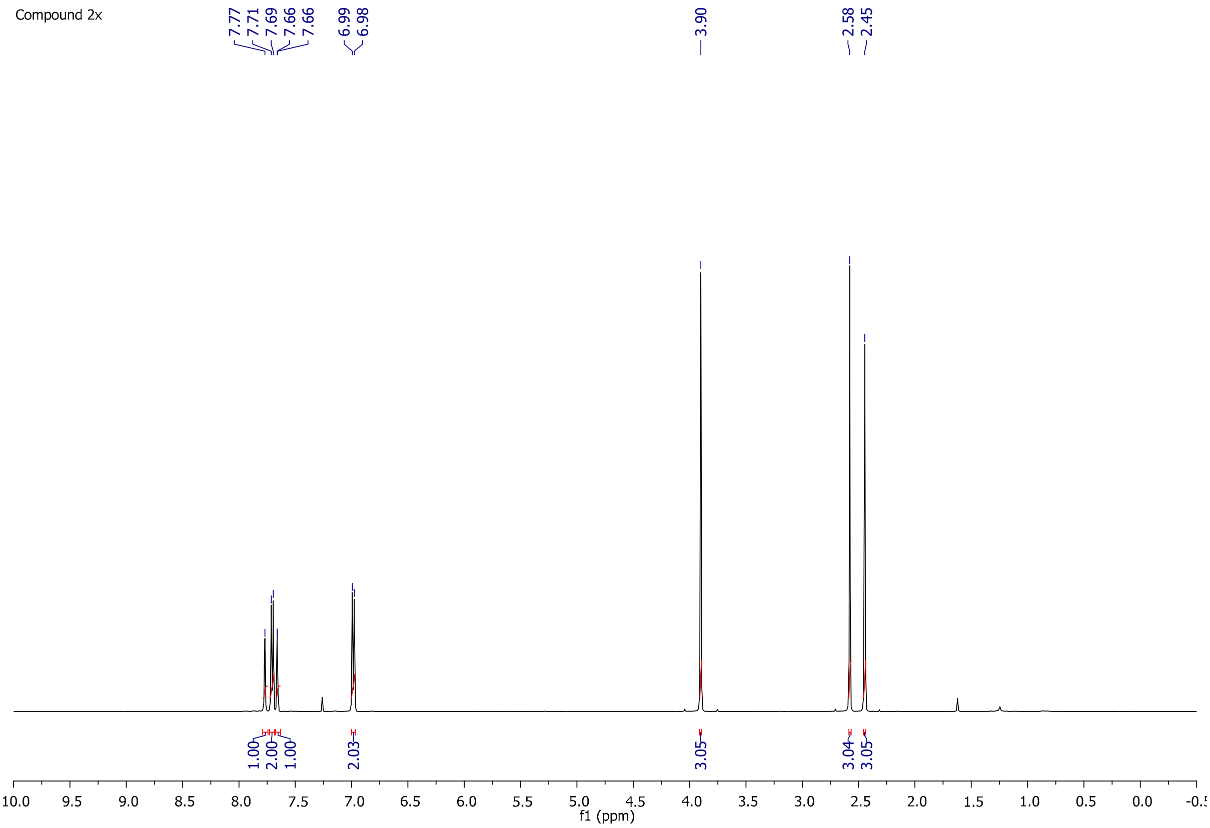

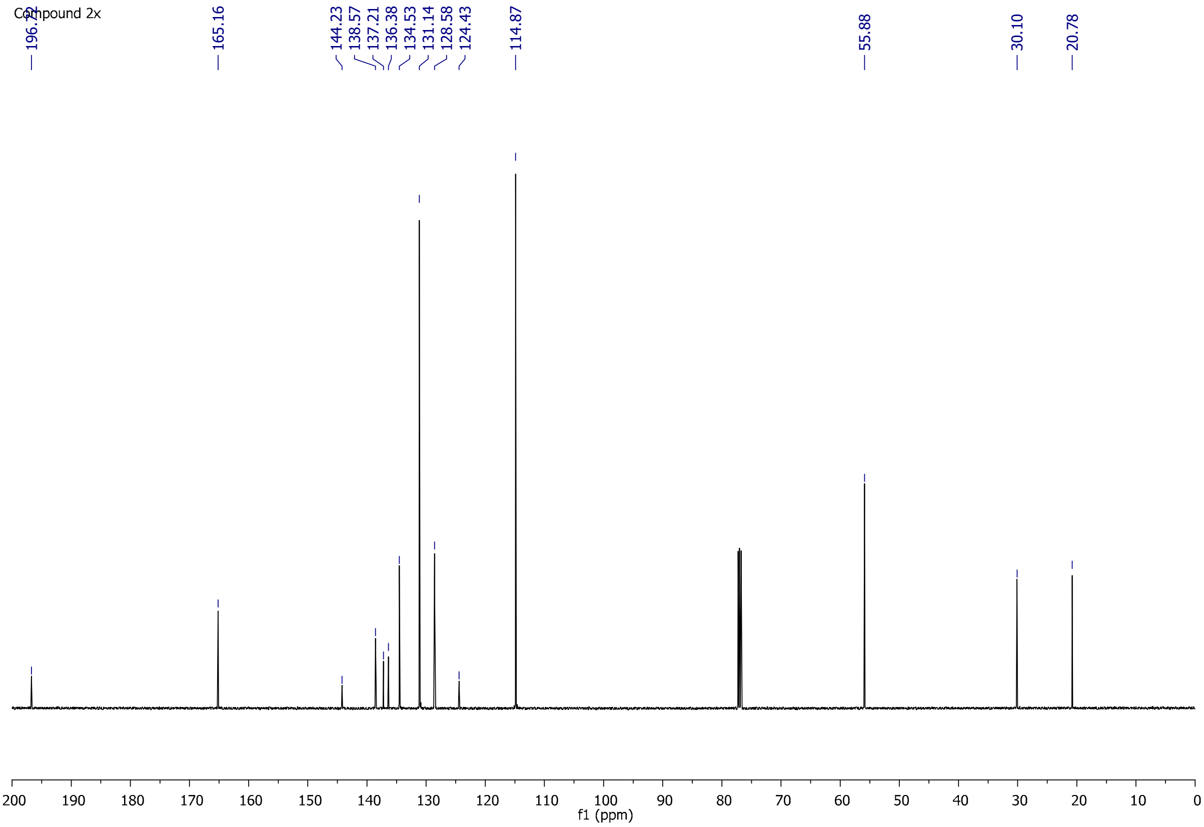

Figure S1.24: ^{1}H- and ^{13}C- NMR of **2x** in $CDCl_3$ at 500 and 125 MHz, respectively.

Table S1. Crystal data collection and structure refinement for **2o**.

**CCDC**	**2380079**
Empirical formula	$C_{14}H_9BrFNO_6S$
Formula weight	418.19
Crystal system	Monoclinic
Space group	*P*21/c
a, b, c (Å)	7.6991(3), 23.8085(8), 8.3551(3)
β (°)	98.1520(10)
Volume (Å^3)	1516.05(10)
Z	4
Density (calc) *g/cm*^{3}	1.832
μ (mm^{-1})	2.893
F(000)	832
Crystal size (mm^3)	0.360 x 0.343 x 0.217
$\theta_{min}/\theta_{max}$ (°)	2.673/27.987
Index ranges	-10 ≤ h ≤ 10, -31 ≤ k≤ 31, -11 ≤ -1 ≤ 11
Reflections collected	75431
Independent reflections	33645 [*R*(int) = 0.0259]
Data/restraints/parameters	3645/ 0/ 218
Goodness-of-fit on F^2	1.044
Final *R* indexes [$I \geq 2\sigma (I)$]	R_1 = 0.0236, wR_2 = 0.0606
Final R indexes [all data]	R_1 = 0.0255, wR_2 = 0.0620
Largest diff. peak/hole (*e.Å*$^{-3}$)	0.816 and -0.657

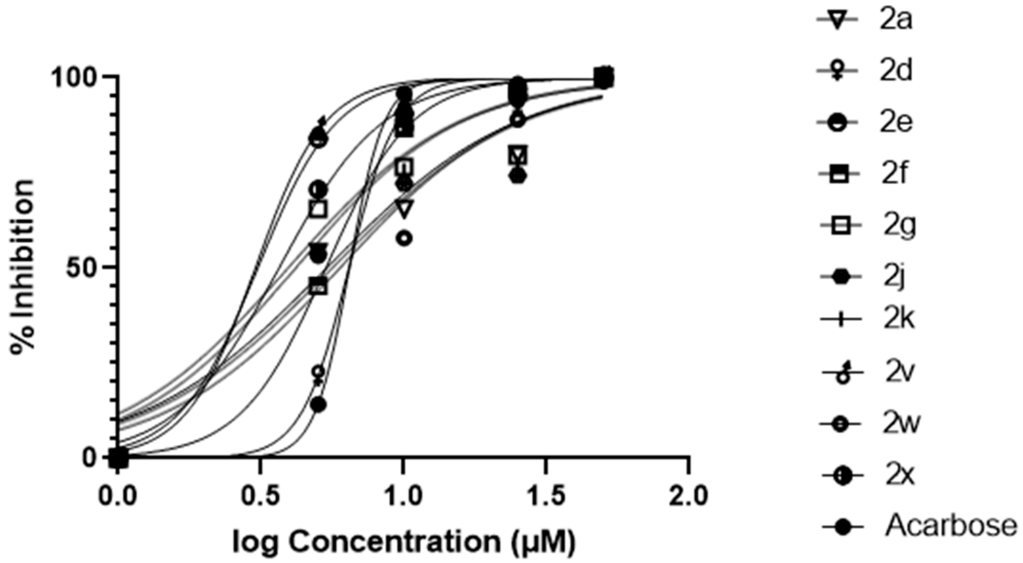

Figure S2: %Inhibition curves of the most active compounds **2** against α-glucosidase.

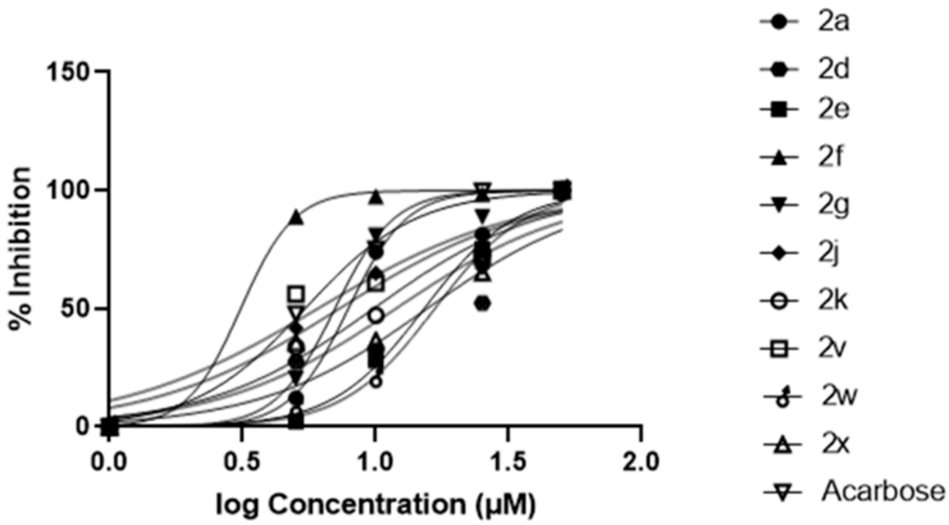

Figure S3: %Inhibition curves of selected compounds **2** against α-amylase.

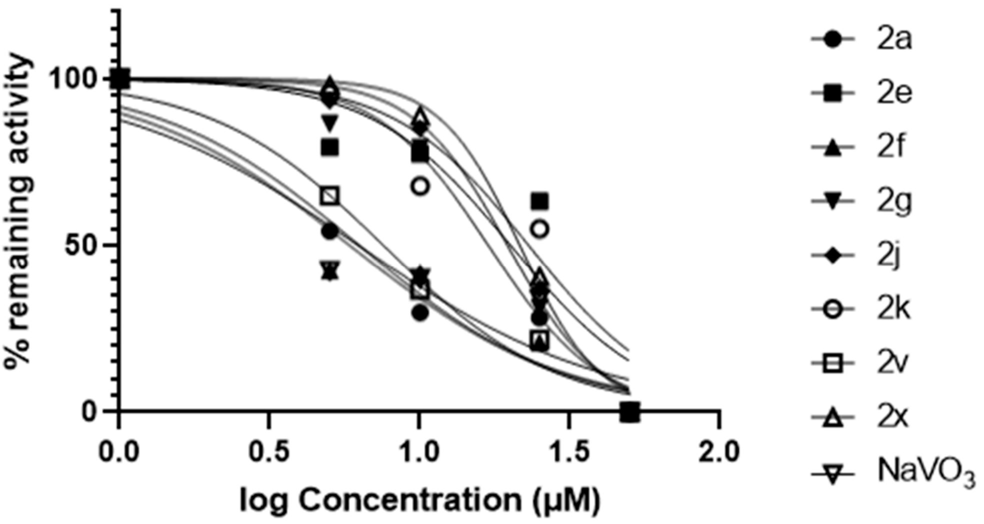

Figure S4: %Inhibition curves of selected compounds **2** against PTP1B.

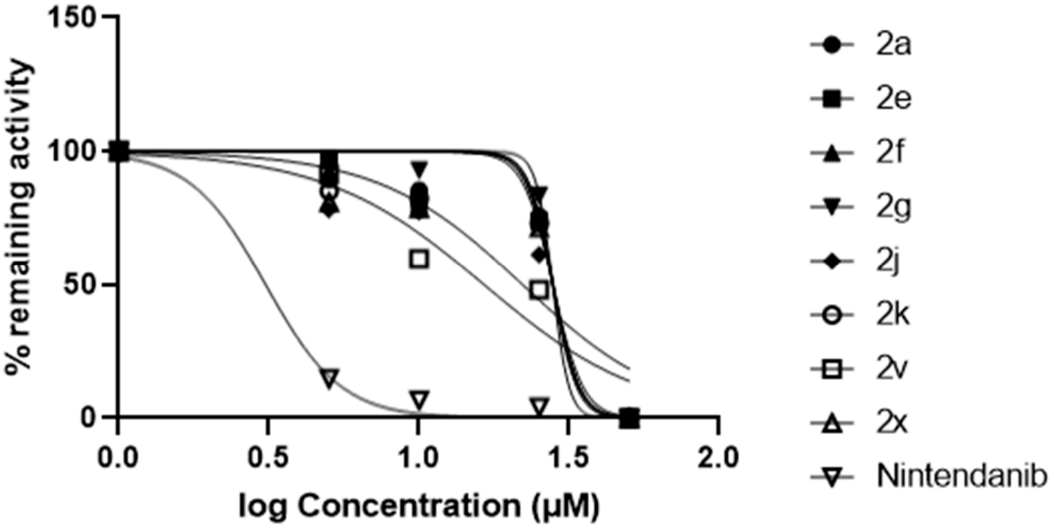

Figure S5: %Inhibition curves of selected compounds **2** against VEGFR-2.

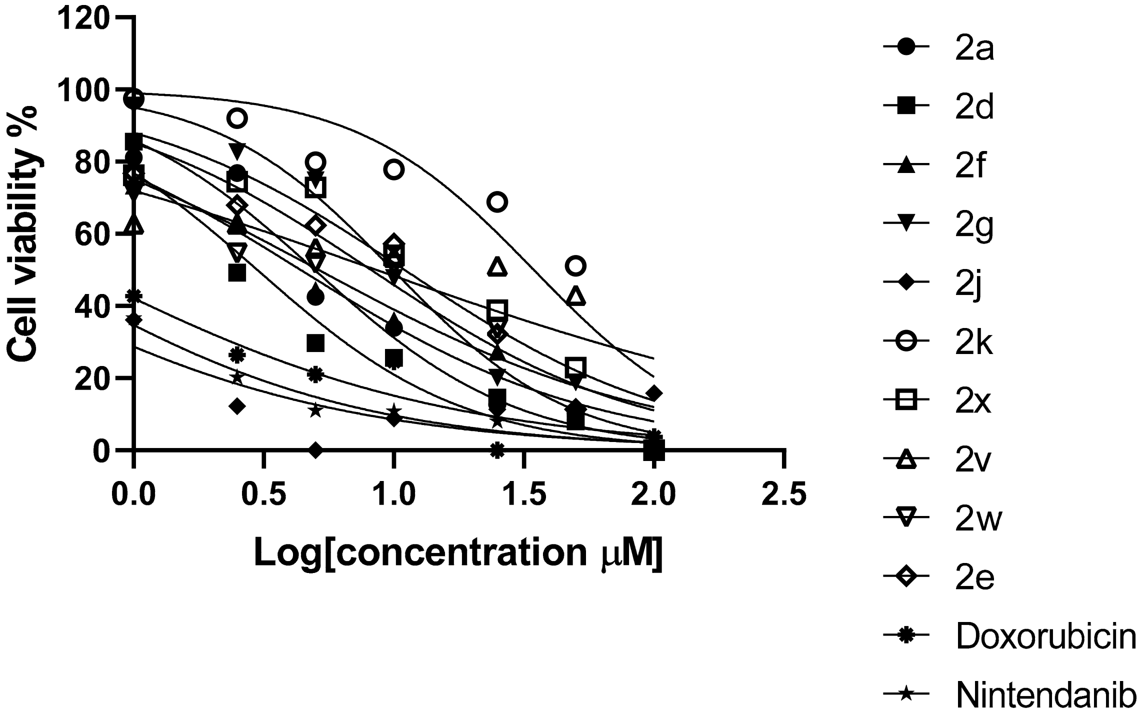

Figure S6: Curves used to calculate the IC_{50} values against the MCF-7 cell line.

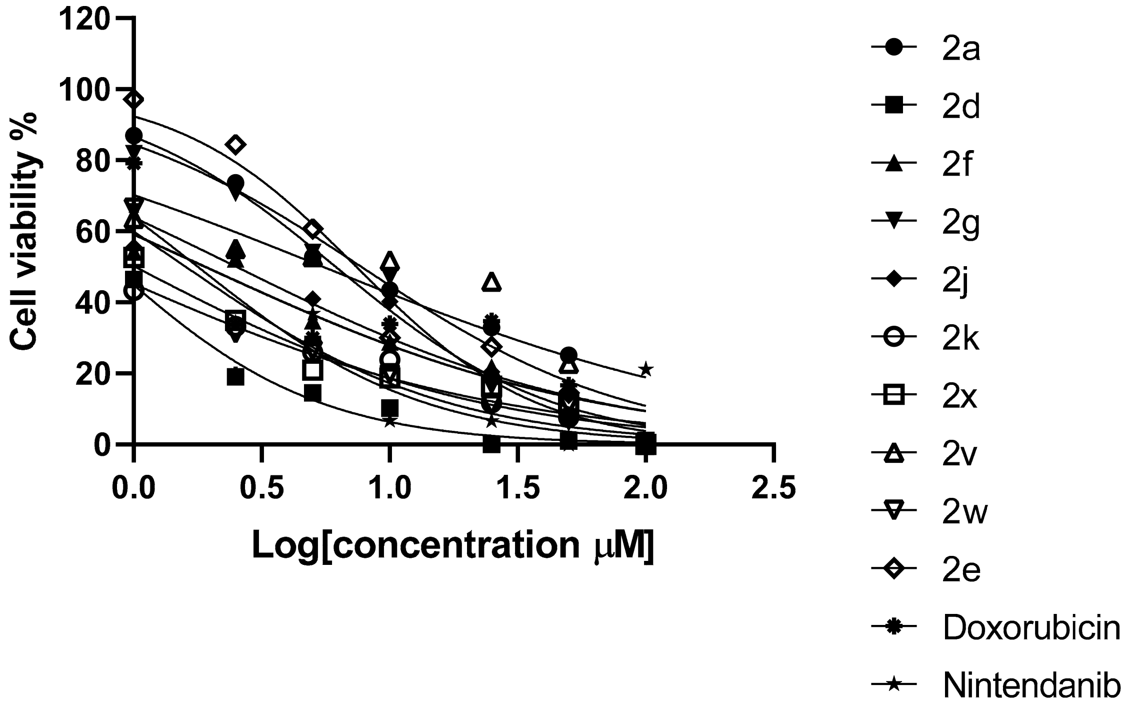

Figure S7: Curves used to calculate the IC_{50} values against the A549 cell line.

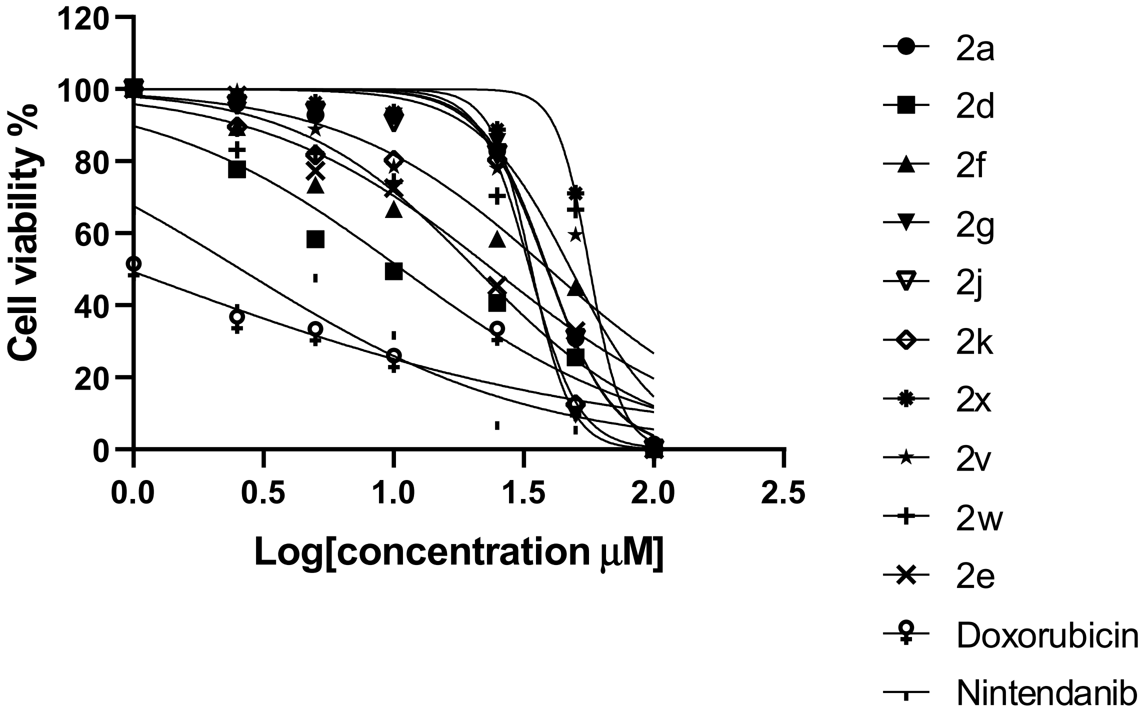

Figure S8: Curves used to calculate the IC_{50} values against the Vero cell line.

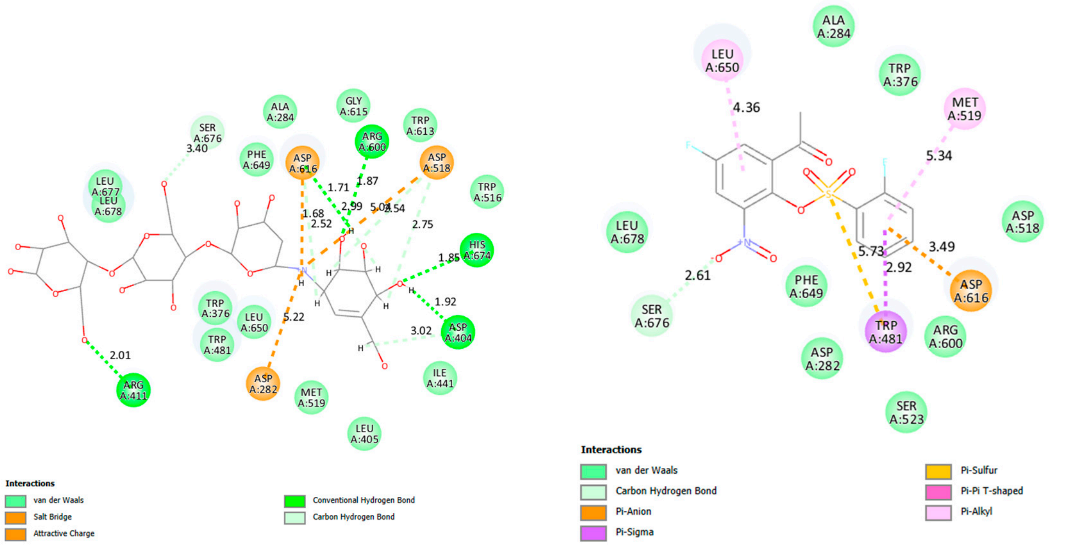

a b

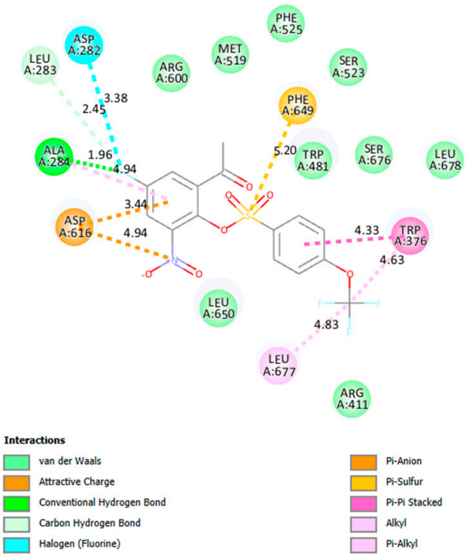

ASP A:282
LEU A:283
ARG A:600
MET A:519
PHE A:525
SER A:523
PHE A:649
ALA A:284
TRP A:481
SER A:676
LEU A:678
ASP A:616
TRP A:376
LEU A:650
LEU A:677
ARG A:411
Interactions
van der Waals
Attractive Charge
Conventional Hydrogen Bond
Carbon Hydrogen Bond
Halogen (Fluorine)
Pi-Anion
Pi-Sulfur
Pi-Pi Stacked
Alkyl
Pi-Alkyl

a

c

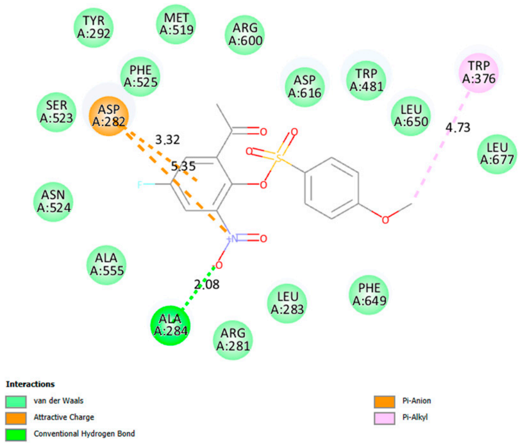

TYR A:292
MET A:519
ARG A:600
PHE A:525
ASP A:616
TRP A:481
TRP A:376
SER A:523
ASP A:282
LEU A:650
LEU A:677
ASN A:524
ALA A:555
ALA A:284
ARG A:281
LEU A:283
PHE A:649
Interactions
van der Waals
Attractive Charge
Conventional Hydrogen Bond
Pi-Anion
Pi-Alkyl

d

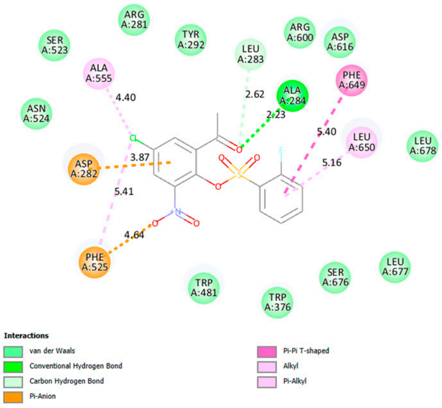

Interactions
van der Waals
Conventional Hydrogen Bond
Carbon Hydrogen Bond
Pi-Anion
Pi-Pi T-shaped
Alkyl
Pi-Alkyl

e

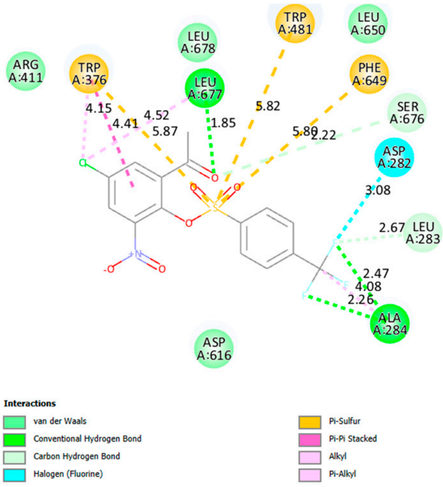

ARG A:411
TRP A:376
LEU A:678
TRP A:481
LEU A:650
LEU A:677
PHE A:649
SER A:676
ASP A:282
LEU A:283
ALA A:284
ASP A:616
Interactions
van der Waals
Conventional Hydrogen Bond
Carbon Hydrogen Bond
Halogen (Fluorine)
Pi-Sulfur
Pi-Pi Stacked
Alkyl
Pi-Alkyl

f

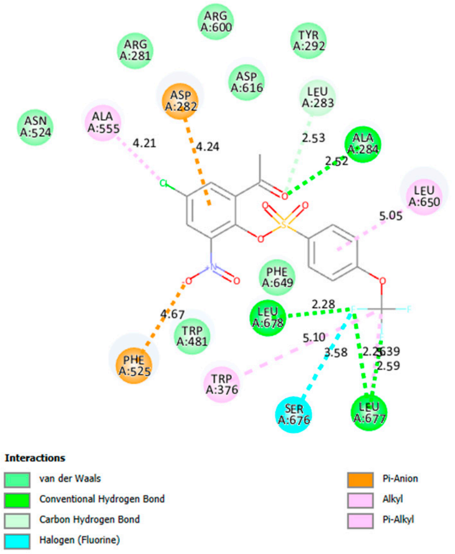

ARG A:600
ARG A:281
TYR A:292
ASP A:616
ASN A:524
ALA A:555
ASP A:282
LEU A:283
ALA A:284
LEU A:650
PHE A:649
LEU A:678
TRP A:481
PHE A:525
TRP A:376
SER A:676
LEU A:677
Interactions
van der Waals
Conventional Hydrogen Bond
Carbon Hydrogen Bond
Halogen (Fluorine)
Pi-Anion
Alkyl
Pi-Alkyl

g

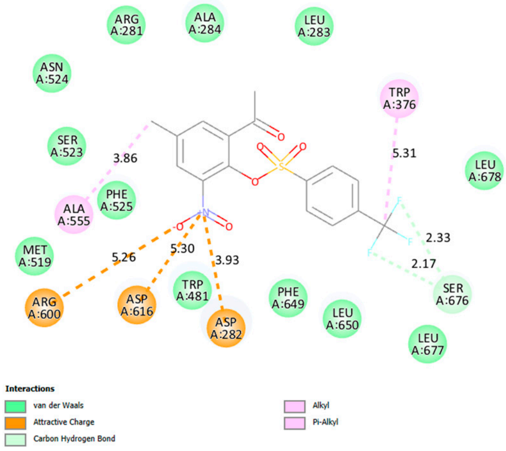

ARG A:281
ALA A:284
LEU A:283
ASN A:524
TRP A:376
SER A:523
LEU A:678
PHE A:525
ALA A:555
MET A:519
TRP A:481
ARG A:600
ASP A:616
ASP A:282
PHE A:649
LEU A:650
SER A:676
LEU A:677
Interactions
van der Waals
Attractive Charge
Carbon Hydrogen Bond
Alkyl
Pi-Alkyl

h

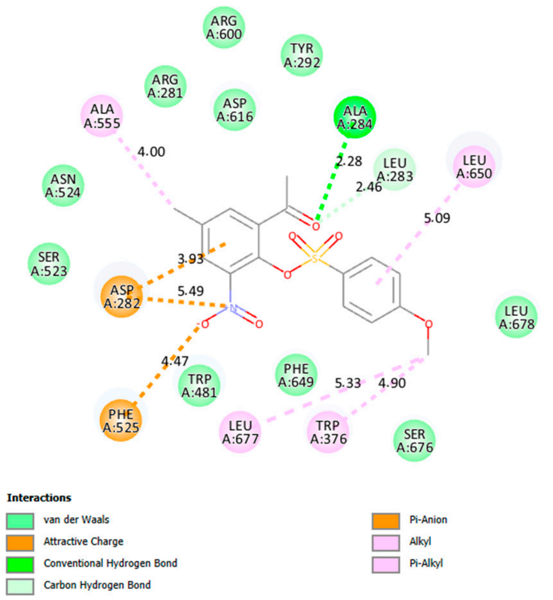

`i

Figure S9: The interactions of acarbose (**a**), **2a** (**b**), **2e** (**c**), **2f** (**d**), **2g** (**e**), **2j** (**f**), **2k** (**g**), **2v** (**h**) and **2x** (**i**) with human lysosomal acid-α-glucosidase (PDB code 5NN8).

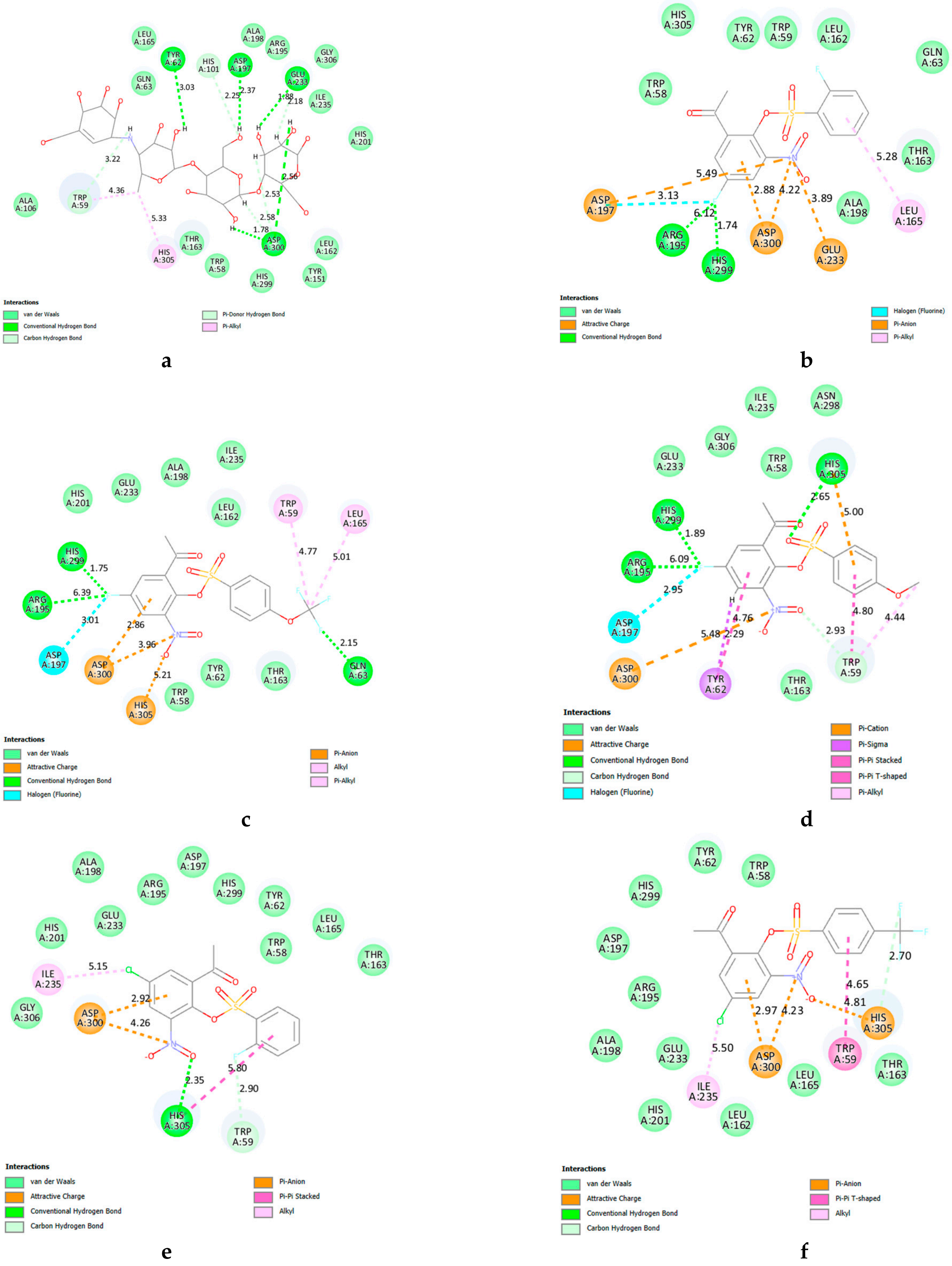
a
Interactions
van der Waals
Conventional Hydrogen Bond
Carbon Hydrogen Bond
Pi-Donor Hydrogen Bond
Pi-Alkyl
b
Interactions
van der Waals
Attractive Charge
Conventional Hydrogen Bond
Halogen (Fluorine)
Pi-Anion
Pi-Alkyl
c
Interactions
van der Waals
Attractive Charge
Conventional Hydrogen Bond
Halogen (Fluorine)
Pi-Anion
Alkyl
Pi-Alkyl
d
Interactions
van der Waals
Attractive Charge
Conventional Hydrogen Bond
Carbon Hydrogen Bond
Halogen (Fluorine)
Pi-Cation
Pi-Sigma
Pi-Pi Stacked
Pi-Pi T-shaped
Pi-Alkyl
e
Interactions
van der Waals
Attractive Charge
Conventional Hydrogen Bond
Carbon Hydrogen Bond
Pi-Anion
Pi-Pi Stacked
Alkyl
f
Interactions
van der Waals
Attractive Charge
Conventional Hydrogen Bond
Carbon Hydrogen Bond
Pi-Anion
Pi-Pi T-shaped
Alkyl

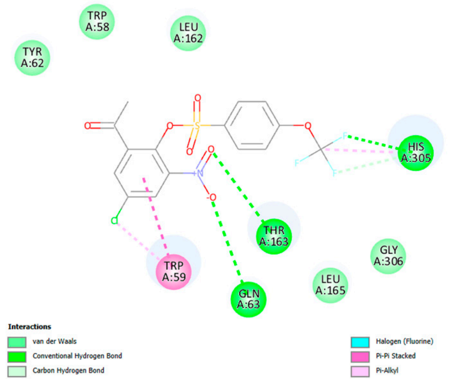

g

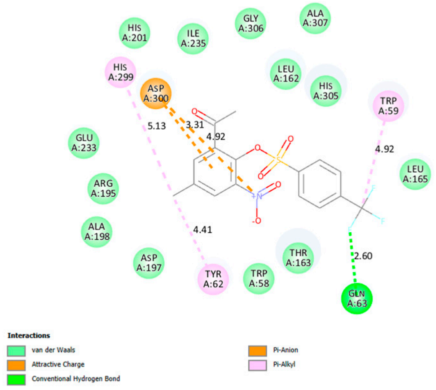

h

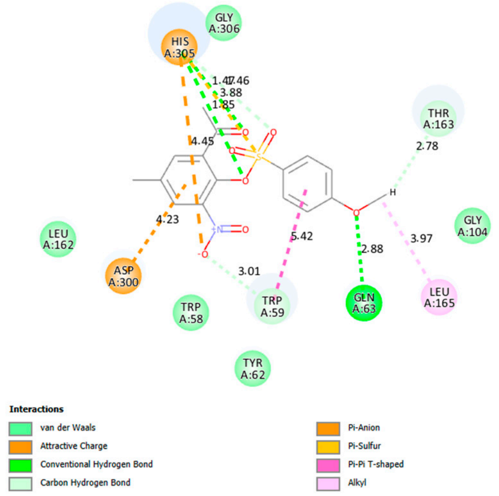

i

Figure S10: The interactions of acarbose (**a**), **2a** (**b**), **2e** (**c**), **2f** (**d**), **2g** (**e**), **2j** (**f**), **2k** (**g**), **2v** (**h**) and **2x** (**i**) with human pancreatic α-amylase (PDB code 3BAJ)

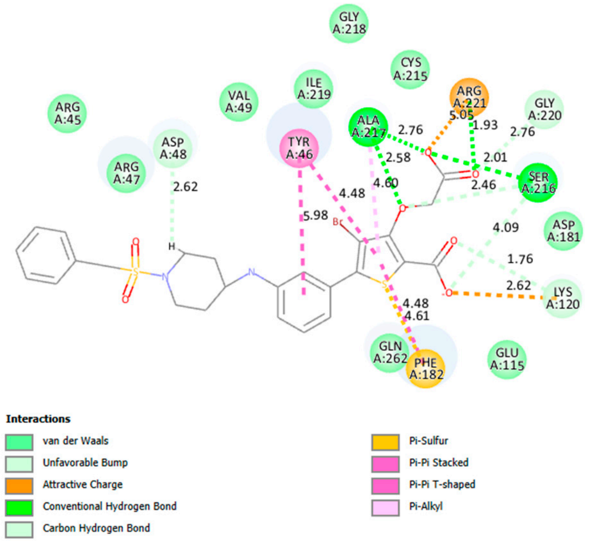

a

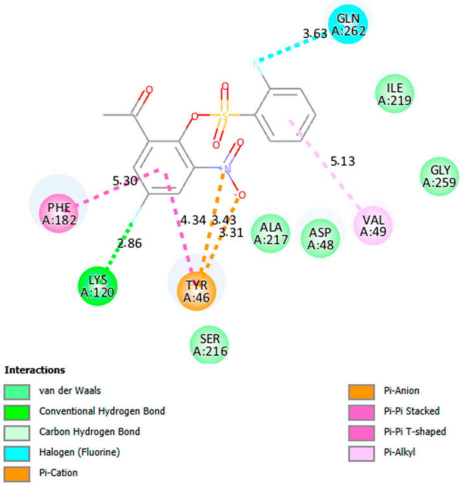

b

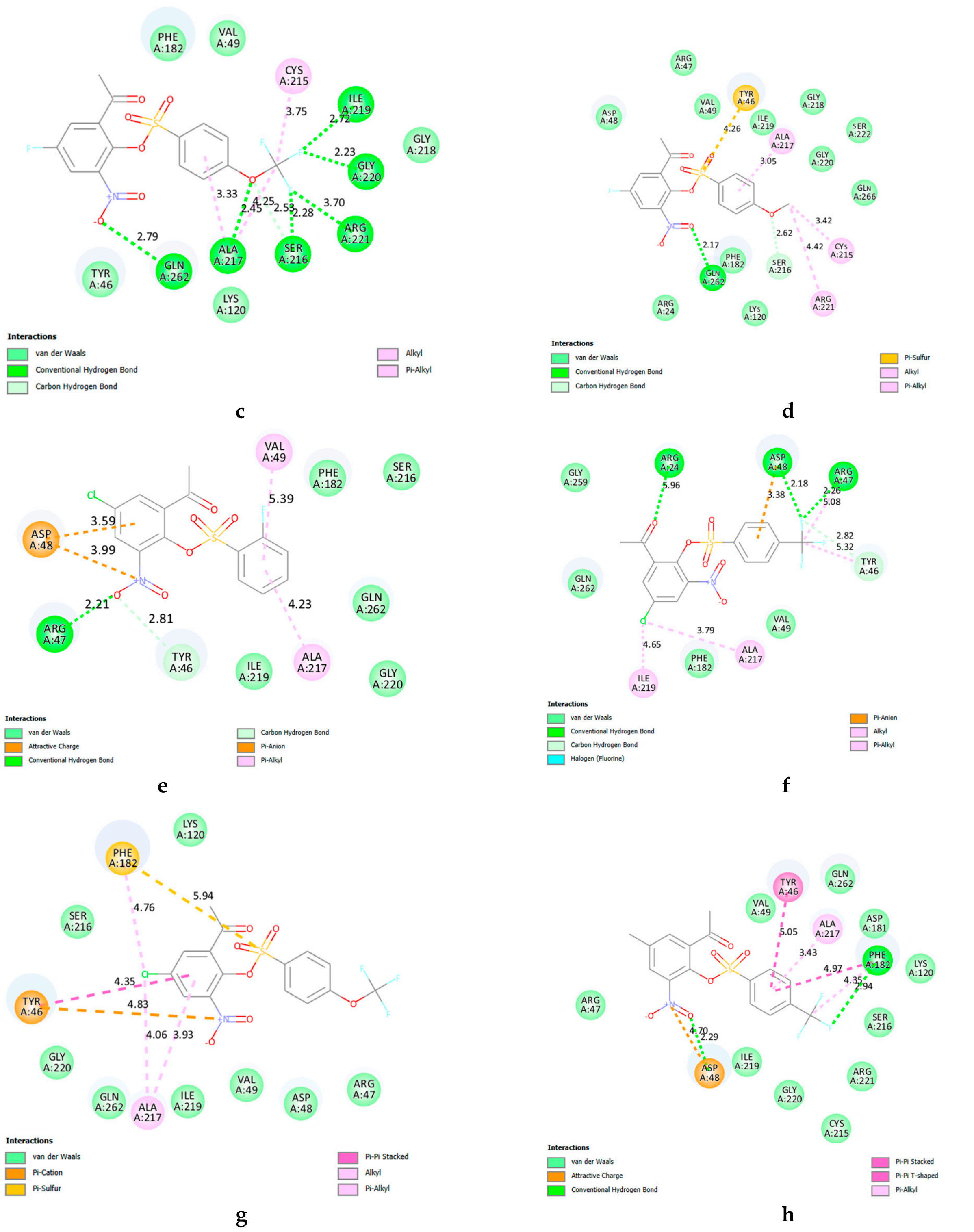

PHE A:182
VAL A:49
CYS A:215
ILE A:219
GLY A:218
GLY A:220
ARG A:221
SER A:216
ALA A:217
LYS A:120
GLN A:262
TYR A:46
Interactions
van der Waals
Conventional Hydrogen Bond
Carbon Hydrogen Bond
Alkyl
Pi-Alkyl
c
Interactions
van der Waals
Conventional Hydrogen Bond
Carbon Hydrogen Bond
Pi-Sulfur
Alkyl
Pi-Alkyl
d
VAL A:49
PHE A:182
SER A:216
ASP A:48
ARG A:47
TYR A:46
ILE A:219
ALA A:217
GLN A:262
GLY A:220
Interactions
van der Waals
Attractive Charge
Conventional Hydrogen Bond
Carbon Hydrogen Bond
Pi-Anion
Pi-Alkyl
e
GLY A:259
ARG A:24
ASP A:48
ARG A:47
TYR A:46
GLN A:262
VAL A:49
PHE A:182
ALA A:217
ILE A:219
Interactions
van der Waals
Conventional Hydrogen Bond
Carbon Hydrogen Bond
Halogen (Fluorine)
Pi-Anion
Alkyl
Pi-Alkyl
f
PHE A:182
LYS A:120
SER A:216
TYR A:46
GLY A:220
GLN A:262
ALA A:217
ILE A:219
VAL A:49
ASP A:48
ARG A:47
Interactions
van der Waals
Pi-Cation
Pi-Sulfur
Pi-Pi Stacked
Alkyl
Pi-Alkyl
g
TYR A:46
GLN A:262
VAL A:49
ALA A:217
ASP A:181
PHE A:182
LYS A:120
ARG A:47
SER A:216
ASP A:48
ILE A:219
GLY A:220
ARG A:221
CYS A:215
Interactions
van der Waals
Attractive Charge
Conventional Hydrogen Bond
Pi-Pi Stacked
Pi-Pi T-shaped
Pi-Alkyl
h

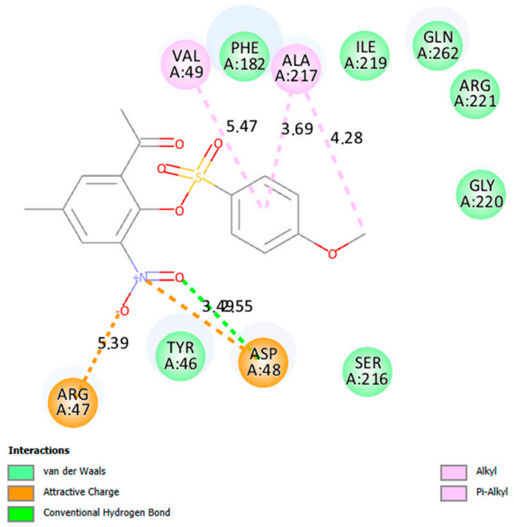

i

Figure S11: The interactions of native ligand (a), **2a** (**b**), **2e** (**c**), **2f** (**d**), **2g** (**e**), **2j** (**f**), **2k** (**g**), **2v** (**h**) and **2x** (**i**) with PTP1B active site (PDB 2QBP)

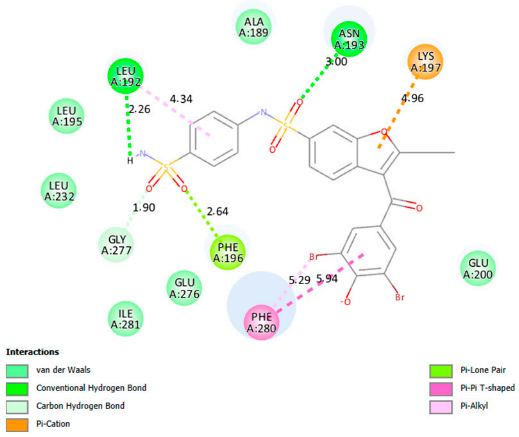

a

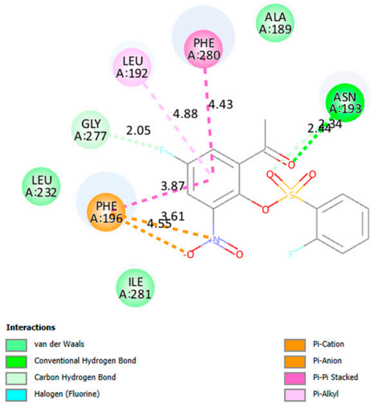

b

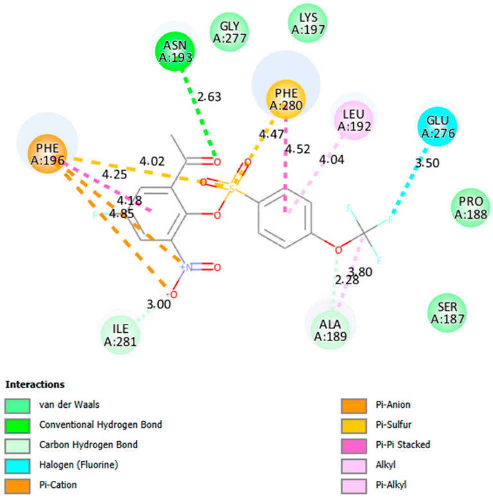

c

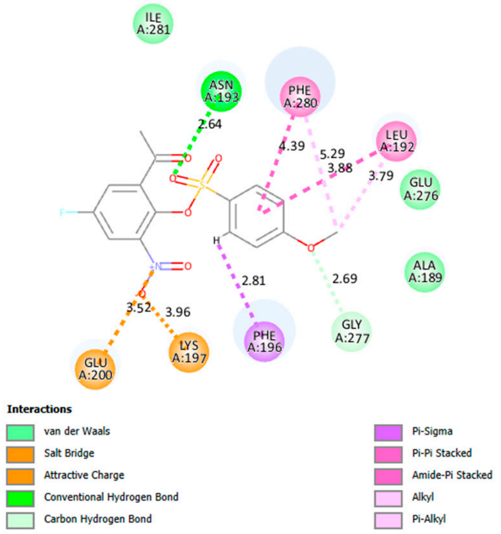

d

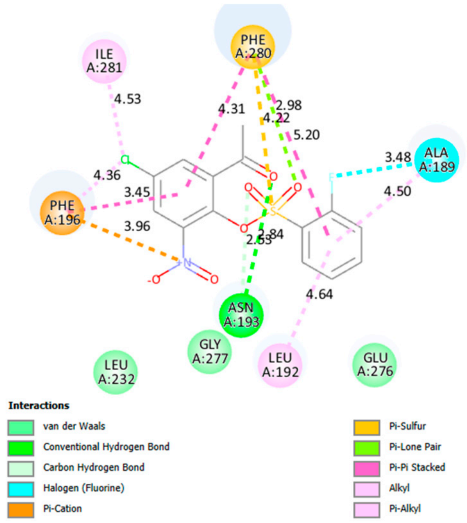

e

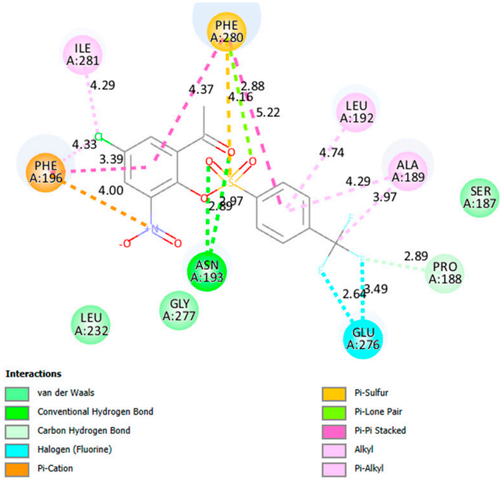

f

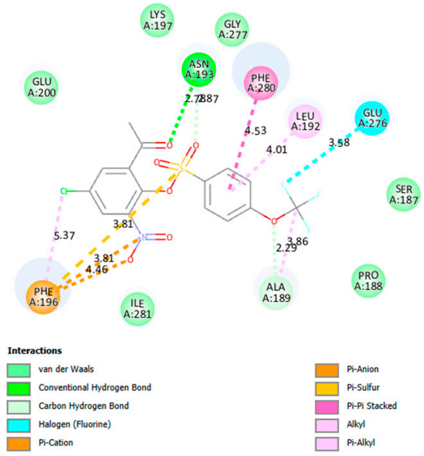

g

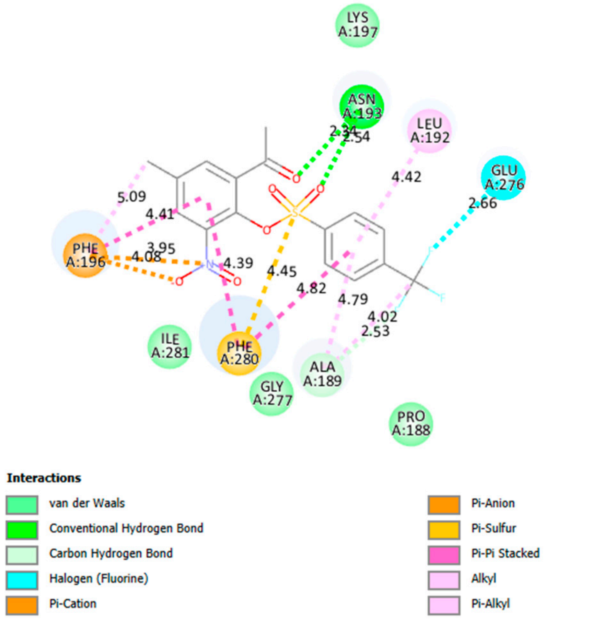

h

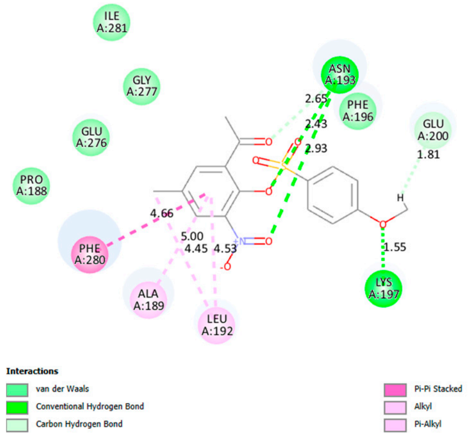

i

Figure S12: The interactions of native ligand (a), **2a** (**b**), **2e** (**c**), **2f** (**d**), **2g** (**e**), **2j** (**f**), **2k** (**g**), **2v** (**h**) and **2x** (**i**) with PTP1B allosteric site (PDB 1T49).

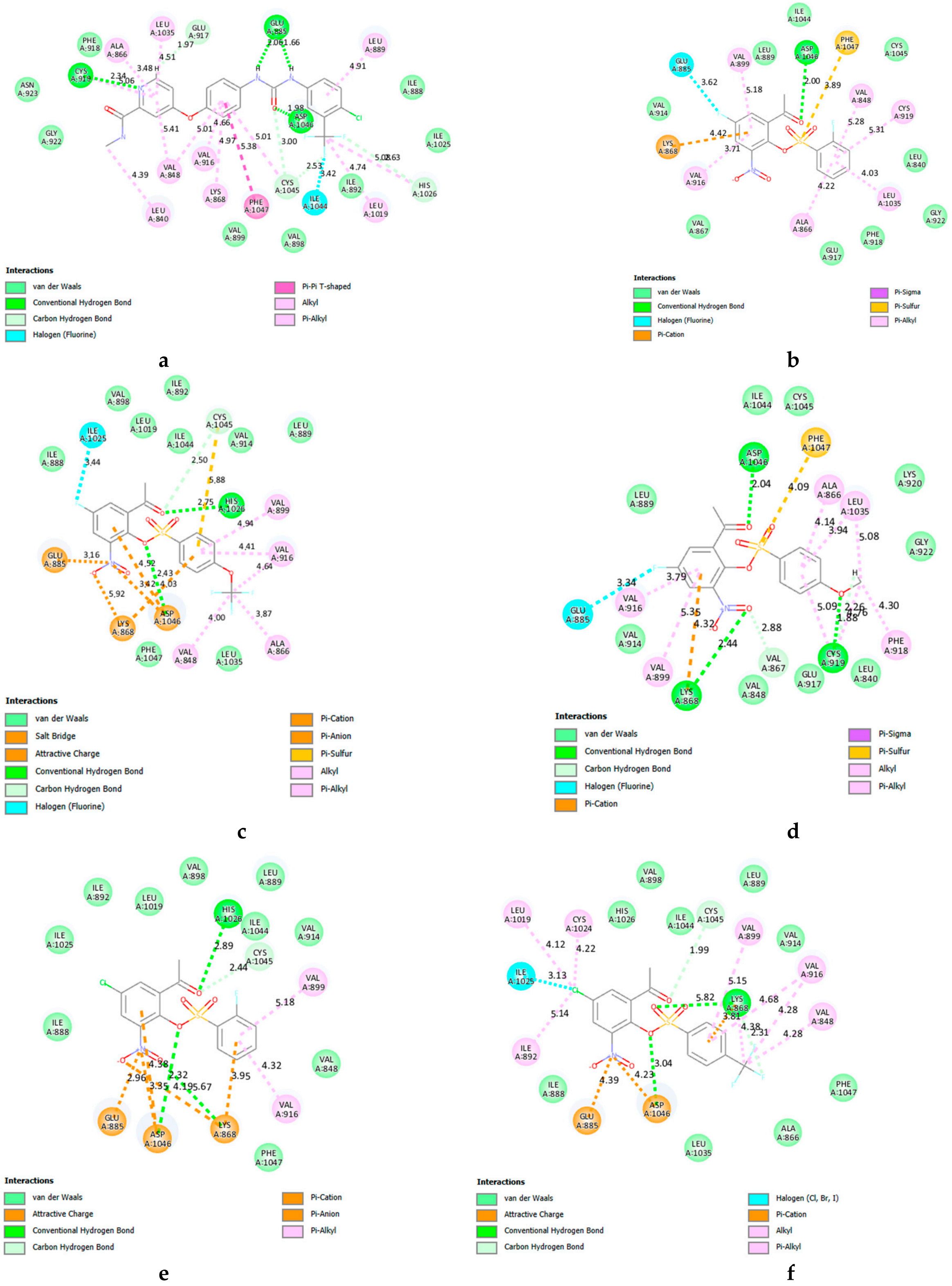
Interactions
van der Waals
Conventional Hydrogen Bond
Carbon Hydrogen Bond
Halogen (Fluorine)
Pi-Pi T-shaped
Alkyl
Pi-Alkyl
a
Interactions
van der Waals
Conventional Hydrogen Bond
Halogen (Fluorine)
Pi-Cation
Pi-Sigma
Pi-Sulfur
Pi-Alkyl
b
Interactions
van der Waals
Salt Bridge
Attractive Charge
Conventional Hydrogen Bond
Carbon Hydrogen Bond
Halogen (Fluorine)
Pi-Cation
Pi-Anion
Pi-Sulfur
Alkyl
Pi-Alkyl
c
Interactions
van der Waals
Conventional Hydrogen Bond
Carbon Hydrogen Bond
Halogen (Fluorine)
Pi-Cation
Pi-Sigma
Pi-Sulfur
Alkyl
Pi-Alkyl
d
Interactions
van der Waals
Attractive Charge
Conventional Hydrogen Bond
Carbon Hydrogen Bond
Pi-Cation
Pi-Anion
Pi-Alkyl
e
Interactions
van der Waals
Attractive Charge
Conventional Hydrogen Bond
Carbon Hydrogen Bond
Halogen (Cl, Br, I)
Pi-Cation
Alkyl
Pi-Alkyl
f

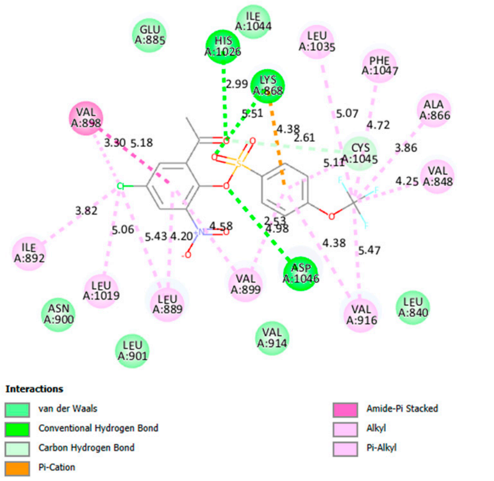

g

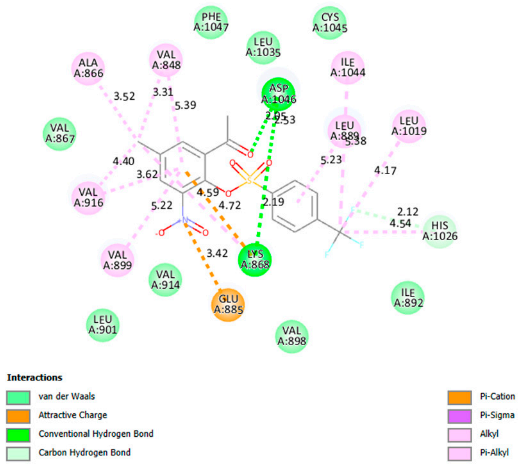

h

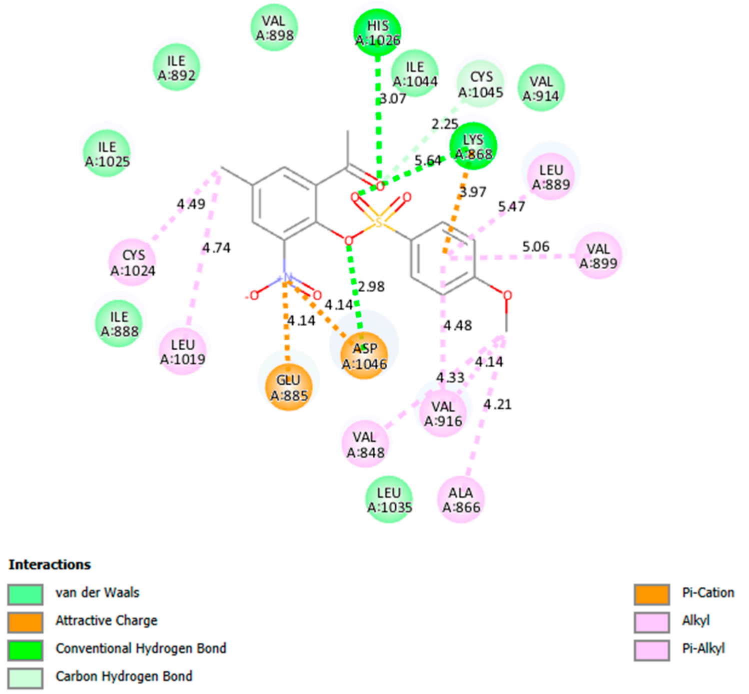

i

Figure S13: The interactions of Sorafenib (a), **2a** (**b**), **2e** (**c**), **2f** (**d**), **2g** (**e**), **2j** (**f**), **2k** (**g**), **2v** (**h**) and **2x** (**i**) with VEGFR-2 active site (PDB 4ASD).
